# Tandem hydrothiocyanation/cyclization of CF_3_-iminopropargyl alcohols with NaSCN in the presence of AcOH

**DOI:** 10.3762/bjoc.21.207

**Published:** 2025-12-16

**Authors:** Ruslan S Shulgin, Ol’ga G Volostnykh, Anton V Stepanov, Igor’ A Ushakov, Alexander V Vashchenko, Olesya A Shemyakina

**Affiliations:** 1 A.E. Favorsky Irkutsk Institute of Chemistry, Siberian Branch, Russian Academy of Science, 1 Favorsky Str., 664033 Irkutsk, Russia

**Keywords:** CF_3_-alkynyl imines, hydrothiocyanation, isothiazolium thiocyanates, propargyl alcohols, sodium thiocyanate

## Abstract

The synthesis of trifluoromethylated isothiazolium thiocyanates and 4-thiocyanato-2,5-dihydrofurans is presented through hydrothiocyanation/cyclization of CF_3_-iminopropargyl alcohols using NaSCN in AcOH/MeCN. The formation of the two products can be explained by different directions of cyclization of the primary adducts of thiocyanic acid at the triple bond – vinyl thiocyanates. This protocol features simple operating, readily prepared starting materials and occurs under relatively mild conditions.

## Introduction

The hydrofunctionalization of alkynes is one of the most effective and convenient ways of synthesizing polysubstituted alkenes [1–5]. Such reactions can also afford more complex products, provided that the vinyl intermediates formed during the reaction undergo further transformations [6–8]. However, this is mainly possible in the case of functionalized alkynes, where these intramolecular reactions usually involve other functional groups that are contained in the same intermediate.

Among the numerous hydrofunctionalization reactions, hydrothiocyanation has attracted much attention from chemists as it is one of the most expedient and direct methods for the formation of a new C–S bond [9–11]. Thiocyanates represent a valuable class of molecules serving as versatile intermediates [12–15] in the synthesis of a broad range of organosulfur compounds, including thiols, thioethers, disulfides, phosphonothioates, and trifluoromethyl sulfides. Additionally, due to the presence of two electrophilic sites (the sulfur atom and the carbon of the nitrile function), thiocyanates can readily undergo domino-type intramolecular cyclization reactions to form heterocycles. So, if a thiocyanate group is introduced into a molecule bearing a nucleophile in a suitable position, cyclization can readily occur.

**Scheme 1:**
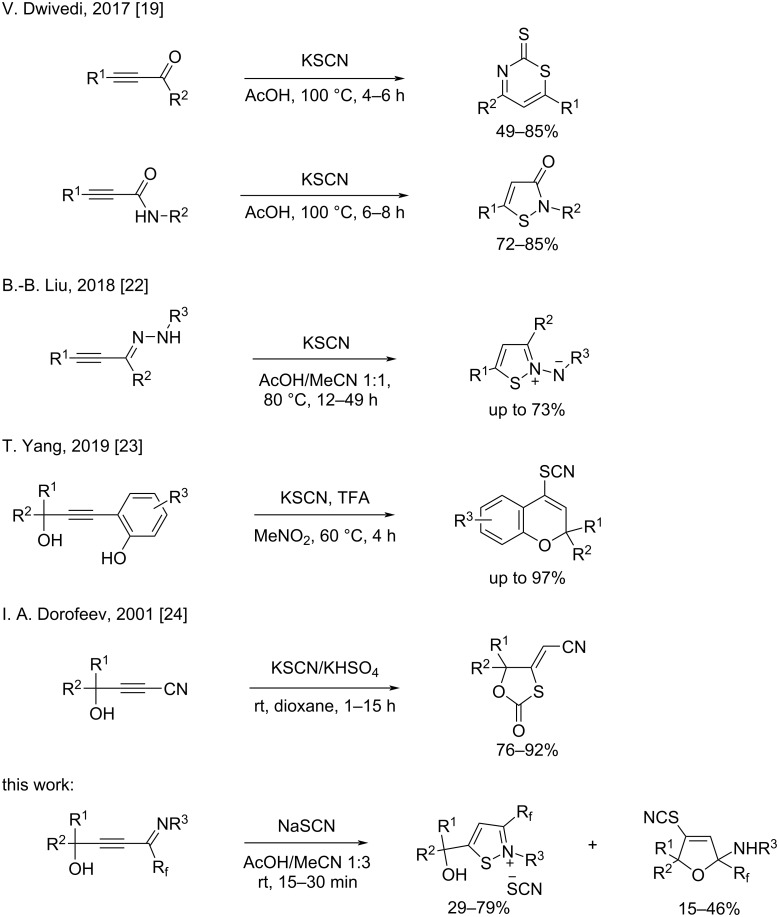
Examples of hydrothiocyanation/cyclization of alkynes.

In recent years, several methods for the hydrothiocyanation of alkynes bearing functional groups, primarily alkynoates, have been developed. Thus, thiocyano enoates were synthesized via ionic liquid- [16] or lactic acid-catalyzed [17] reactions of alkynoates, KSCN and water under ultrasound conditions as well as by the reactions of alkynoates, KSCN and water using deep eutectic solvents [18]. Reddy and co-workers [19] proposed an approach to thiocyano enones through the hydrothiocyanation of ynones using KSCN in AcOH at 70 °C. They also demonstrated that the adducts of ynones are readily cyclized in situ via a second thiocyanation to form thiazine-2-thiones at a slightly higher temperature and for a longer time. Meanwhile the reaction with ynamides led to decyanative amido cyclization to isothiazolones (Scheme 1). Silver- [20] and gold-catalyzed [21] hydrothiocyanations of haloalkynes with thiocyanate salts in acetic acid to give *Z*-vinyl thiocyanates were reported. The reaction between alkynic hydrazones and KSCN in AcOH/MeCN delivered *N*-iminoisothiazolium ylides [22] (Scheme 1). A TFA-mediated cascade cyclization of 2-propynolphenols using KSCN as the thiocyanation source led to the efficient formation of various 4-thiocyanated 2*H*-chromenes has been developed [23]. 1,3-Oxathiolan-2-ones were obtained by treating 4-hydroxy-2-alkynonitriles with the KSCN/KHSO_4_ system [24]. In light of the above examples and as part of our ongoing research into the chemistry of functionalized propargyl alcohols [25–26], we decided to use CF_3_-substituted iminopropargyl alcohols as a starting material for hydrothiocyanation reactions, since the alkynyl imine fragment can undergo a series of different cyclizations due to the conjugated imine and alkyne bonds [27], and the hydroxyalkyl moiety linked to the triple bond can also act as a nucleophilic site. Another attractive feature of these compounds is the presence of the trifluoromethyl group (CF_3_). The latter serves as a valuable structural motif in various biologically active molecules and materials due to its unique physical, chemical, and biological properties [28–30]. SCN and trifluoromethyl groups have proved to be versatile moieties in the design and development of numerous active pharmaceutical ingredients, agrochemicals, drugs, and organic functional materials. Additionally, CF_3_-substituted iminopropargyl alcohols can be easily prepared with good yields from commercially available materials in two steps [31–32]. Here, we report for the first time on the hydrothiocyanation of CF_3_-substituted iminopropargyl alcohols with the MSCN/acid system. In comparison to ynamides [19] and alkynehydrazones [22], whose adducts cyclize with the involvement of nitrogen functional groups at elevated temperatures and extended reaction times, the reaction of CF_3_-substituted iminopropargyl alcohols occurred at room temperature within 15 min.

## Results and Discussion

We commenced our investigation using CF_3_-subsituted iminopropargyl alcohol **1a** and NaSCN as model substrates. The completion of the reaction was monitored using IR spectroscopy to observe the disappearance of the band at 2219 cm^−1^ (–C≡C–). It was found that CF_3_-iminopropargyl alcohol **1a** reacted with the NaSCN/acid system to afford a mixture of major isothiazolium thiocyanate **2a** and minor 4-thiocyanato-2,5-dihydrofuran **3a**. No products were observed in the absence of acid (Et_2_O/H_2_O or Me_2_CO).

Next, an investigation was conducted to determine the most suitable combination of acid and solvent. In a hexane/H_2_O biphasic system upon vigorous stirring, the reaction of CF_3_-iminopropargyl alcohol **1a** and the NaSCN/KHSO_4_ system (ratio of **1a**/NaSCN/acid 1:2:2) gave products **2a** and **3a** in 31% and 10% yields, respectively (Table 1, entry 1). Replacing hexane with THF doubled the yield of isothiazolium thiocyanate (Table 1, entry 2). The reaction with chloroacetic acid slightly decreased the yield of **2a** (Table 1, entry 4). The use of H_2_SO_4_ in THF/H_2_O gave lower yields of products due to side processes (Table 1, entry 5). However, the change of THF for Me_2_CO afforded products **2a** and **3a** in 48% and 13% yields, respectively (Table 1, entry 6). The reaction of **1a** with the NaSCN/H_2_SO_4_ system in MeCN delivered product **2a** in a slightly higher yield (Table 1, entry 8). Acetic acid in a ratio **1a**/NaSCN/acid 1:2:2 decreased the yields, as only minor conversion of **1a** (20%) was observed (Table 1, entry 9). Increasing the quantity of acetic acid in the reaction mixture improved the product yields, especially for 2,5-dihydrofuran **3a** (Table 1, entries 10 and 11). The best yield (76%) of **2a** was obtained using acetic acid as co-solvent in a 3:1 (v/v) mixture of MeCN and AcOH (Table 1, entry 12); in this way a total yield of products **2a** and **3a** of 99% could be obtained. When the reaction was carried out with TFA (ratio of **1a**/NaSCN/acid 1:2:2) products **2a** and **3a** were obtained in 62 and 15%, respectively (Table 1, entry 15). However, a further increase of the TFA amount was detrimental to the yield due to undesirable polymerization side reactions (Table 1, entries 16 and 17). As can be seen from Table 1, the use of strong acids reduces the yields of products due to side processes, whereas more polar solvents increase them. Finally, screening of thiocyanate salts showed that NaSCN was the most suitable source of HSCN for this transformation compared to KSCN and NH_4_SCN (Table 1, entry 12 vs entries 18 and 19). The optimization of the reaction conditions showed that the best yields were obtained when the reaction was carried out in AcOH/MeCN 1:3 using 2 equiv of NaSCN (Table 1, entry 12). Isothiazolium salt **2a** and 2,5-dihydrofuran **3a** are easily separable by column chromatography due to their different polarity.

**Table 1:** Screening of reaction conditions.

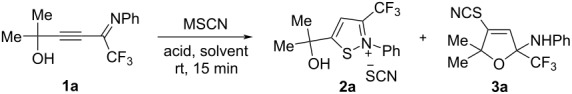						

Entry	MSCN	Acid	Molar ratio **1a**/MSCN/acid	Solvent	Yield of **2a**	Yield of **3a**

1	NaSCN	KHSO_4_	1:2:2	hexane/H_2_O	31%	10%
2	NaSCN	KHSO_4_	1:2:2	THF/H_2_O	61%	6%
3	NaSCN	H_3_PO_4_	1:2:2	THF/H_2_O	24%	6%
4	NaSCN	ClCH_2_CO_2_H	1:2:2	THF/H_2_O	52%	14%
5	NaSCN	H_2_SO_4_	1:2:2	THF/H_2_O	26%	5%
6	NaSCN	H_2_SO_4_	1:2:2	Me_2_CO/H_2_O	48%	13%
7	NaSCN	H_2_SO_4_	1:1:1	Me_2_CO/H_2_O	12%	5%
8	NaSCN	H_2_SO_4_	1:2:2	MeCN	56%	6%
9^a^	NaSCN	AcOH	1:2:2	MeCN	10%	trace
10^b^	NaSCN	AcOH	1:2:4	MeCN	51%	27%
11	NaSCN	AcOH	1:2:–^c^	AcOH/hexane	54%	30%
12	NaSCN	AcOH	1:2:–^d^	AcOH/MeCN	76%	23%
13	NaSCN	AcOH	1:3:–^d^	AcOH/MeCN	73%	25%
14	NaSCN	AcOH	1:2:–^e^	AcOH	54%	13%
15	NaSCN	TFA	1:2:2	MeCN	62%	15%
16	NaSCN	TFA	1:2:4	MeCN	28%	6%
17^f^	NaSCN	TFA	1:2:–^d^	TFA/MeCN	–	–
18	KSCN	AcOH	1:2:–^d^	AcOH/MeCN	71%	25%
19	NH_4_SCN	AcOH	1:2:–^d^	AcOH/MeCN	50%	18%

^a^Conversion of **1a** was 20%; ^b^conversion of **1a** was 90%; ^c^acid was used as a co-solvent in a ratio of 1:6 (AcOH/hexane); ^d^acid was used as a co-solvent in a ratio of 1:3 (AcOH or TFA)/MeCN); ^e^acid was used as a solvent; ^f^strong resinification of reaction mixture and product not detectable.

Next, we investigated the substrate scope and limitations of the reaction using differently substituted iminopropargyl alcohols (Table 2).

**Table 2:** Scope of iminopropargyl alcohols **1**.

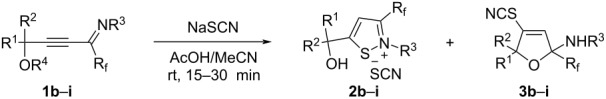					

Entry	Iminopropargyl alcohol, **1**	Product **2**	Yield of **2**	Product **3**	Yield of **3**

1	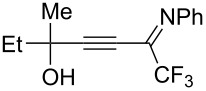 **1b**	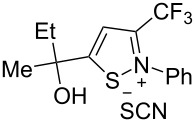 **2b**	79%	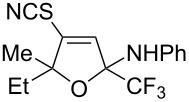 **3b**	21%
2	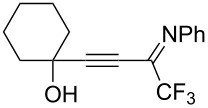 **1c**	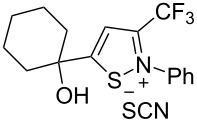 **2c**	71%	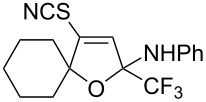 **3c**	15%
3	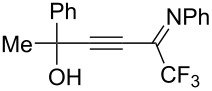 **1d**	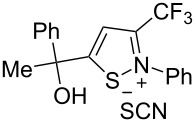 **2d**	29%	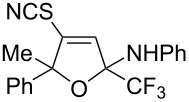 **3d**	25%
4	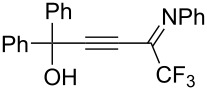 **1e**	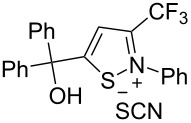 **2e**	31%	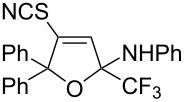 **3e**	17%
5	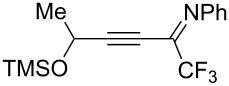 **1f**	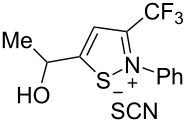 **2f**	–	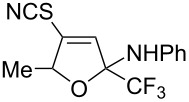 **3f**	46%
6	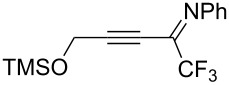 **1g**	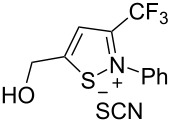 **2g**	–	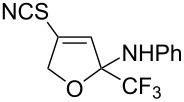 **3g**	44%
7	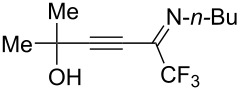 **1h**	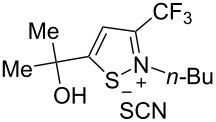 **2h**	59%	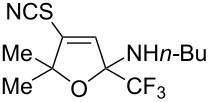 **3h**	19%
8	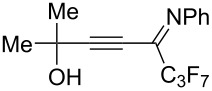 **1i**	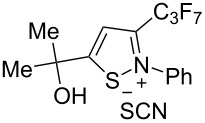 **2i**	75%	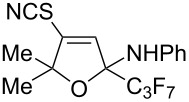 **3i**	18%

The presence of alkyl substituents in the hydroxy fragment was well tolerated, providing products **2b**,**c** and **3b**,**c** with similar yields (Table 2, entries 1 and 2). The slightly decreased yield observed for product **3c** obtained from iminopropargyl alcohol having a cyclohexyl substituent, is probably due to the steric effect (Table 2, entry 2). The more sterically hindered CF_3_-iminopropargyl alcohols with aromatic substituents **1d** (R^1^ = Ph, R^2^ = Me) and **1e** (R^1^ = R^2^ = Ph) were found to be less productive: the reaction required a 2-fold longer time (30 min) and resulted in lower yields of salts **2d**,**e** likely due to side polymerization (Table 2, entries 3 and 4). At the same time, the yields of dihydrofurans **3d**,**e** remained comparable to those of **3a**,**b**. Perhaps the yields of salts **2d**,**e** were affected by steric factors hindering the intramolecular cyclization or their stability under the reaction conditions. Using TMS-protected secondary and primary iminopropargyl alcohols **1f**,**g** (due to their instability) in reaction with the NaSCN/AcOH system failed to afford the corresponding isothiazolium salts **2f**,**g**, and dihydrofurans **3f**,**g** were isolated as the only products (Table 2, entries 5 and 6). In addition, the reaction mixture underwent strong resinification, as primary and secondary propargyl alcohols having electron-withdrawing groups at the triple bond can easily undergo prototropic isomerization to form corresponding unstable allene structures, particularly in acidic conditions. When *N*-*n-*Bu CF_3_-iminopropargyl alcohol **1h** was introduced to the reaction, product **2h** was obtained in a slightly lower yield (59%) compared to **1a** (Table 2, entry 7). Also, C_3_F_7_-iminopropargyl alcohol **1i** was a suitable substrate giving the desired products **2i** and **3i** in 75% and 18% yields, respectively (Table 2, entry 8). Clearly, the results show that the substituents in the hydroxy moiety have a significant impact on formation and ratio of the products in contrast to the substituents in the imine fragment.

**Figure 1:**
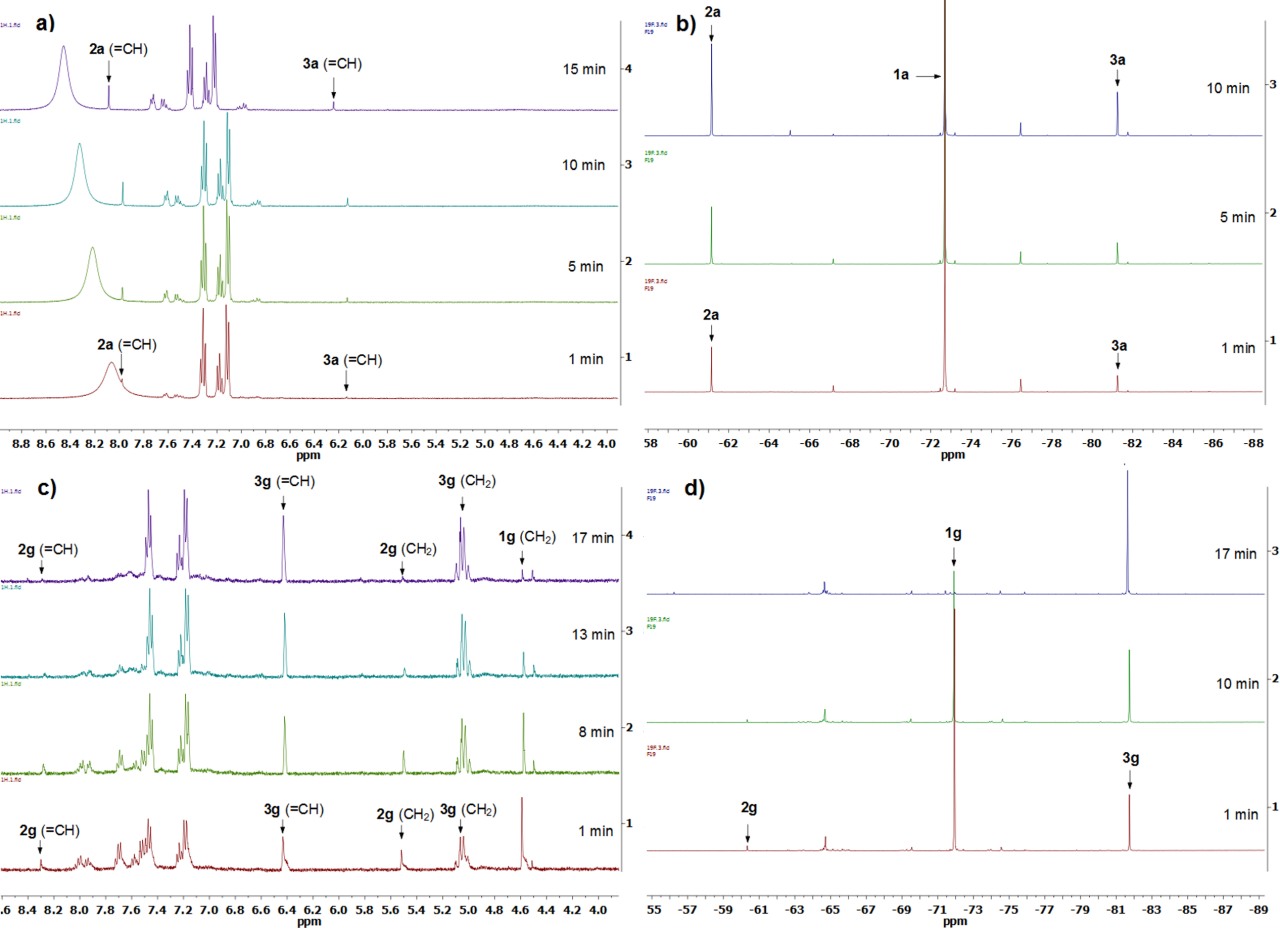
^1^H and ^19^F NMR monitoring of **1a**/NaSCN/AcOH (a, b) and **1g**/NaSCN/AcOH (c, d) reaction mixtures in MeCN.

NMR monitoring of reactions (**1a**/NaSCN/AcOH or **1g**/NaSCN/AcOH in MeCN) did not allow to detect any intermediates even when the temperature was lowered to 0 °C. In the ^1^H and ^19^F NMR spectra the signals of products **2a** and **3a** appeared immediately upon mixing the starting reagents (Figure 1a and b). This indicated that the cyclization occurred almost immediately. Moreover, in the NMR spectra of the **1g**/NaSCN/AcOH reaction mixture in MeCN we detected signals assigned to isothiozolium salt **2g** (^1^H NMR: δ 8.30 s (CH), 5.56 s (CH_2_) ppm, ^19^F NMR: δ 60.3 ppm), that disappeared by the end of the reaction (Figure 1c and d). Probably, the salts **2f**,**g** are unstable and decompose under the reaction conditions.

**Scheme 2:**
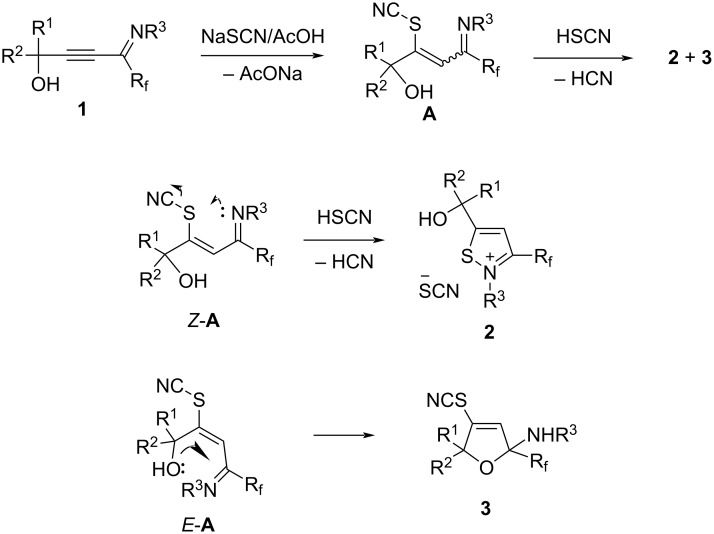
Plausible reaction mechanism.

The formation of products **2** and **3** can be explained by different directions of cyclization of the primary adduct of thiocyanic acid at the triple bond – vinylthiocyanate **A** (Scheme 2). Isothiazolium thiocyanate **2** is formed by the attack of the imino nitrogen atom on the sulfur atom and the elimination of the CN anion in the *Z*-isomer of intermediate **A**. On the other hand, intramolecular addition of the hydroxy group to the imino group in the *E*-isomer of adduct **A** leads to 4-thiocyano-2,5-dihydrofuran **3**. Taking into account the NMR monitoring data, it can be assumed that the yield and ratio of products are influenced by the stereoselectivity of the reaction and the stability of salts **2** under reaction conditions.

**Scheme 3:**
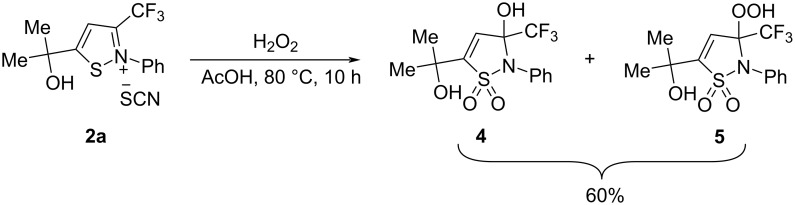
Oxidation of isothiazolium thiocyanate **2a**.

We also carried out a further derivatization experiment. The product **2a** could be oxidized with H_2_O_2_ to produce a mixture of 3-hydroxy- and 3-hydroperoxy-2,3-dihydroisothiazole 1,1-dioxides **4** and **5** in a ratio of 4:1, respectively (Scheme 3).

Such cyclic sulfonamides (sultams) are of interest in the field of medicinal chemistry [33–34] and organic synthesis [35]. For example, they have been found to exhibit inhibitory activity against human leukocyte elastase (HLE) [35] and anti-HIV-1-activity [36]. Hydroperoxy sultams can also act as oxidants [37].

## Conclusion

In summary, we have developed a simple route to trifluoromethylated isothiazolium thiocyanates and 4-thiocyano-2,5-dihydrofurans via the reaction of CF₃-iminopropargyl alcohols with NaSCN/AcOH. The tandem process includes hydrothiocyanation of the triple bond followed by cyclization of the intermediates with the participation of the SCN group and an intramolecular nucleophilic center – imino group or an imino and OH groups. The synthesized compounds are potential drug precursors, synthons, and multipurpose building blocks for fine organic synthesis. We believe that the present method will be of significant interest to synthetic and medicinal chemists due to the operational simplicity, relatively mild conditions and readily prepared starting materials.

## Experimental

**General information.**^1^Н, ^13^С {^1^H}, and ^19^F NMR spectra were recorded on a Bruker DPX-400 spectrometer (400.1, 100.6, and 376 MHz, respectively) in CDCl_3_ and (CD_3_)_2_CO using hexamethyldisiloxane (HMDS) as internal reference at 20–25 °C. IR spectra were measured on a Bruker Vertex-70 instrument in thin layer, films or KВr pellets. Microanalyses were performed on a Flash 2000 elemental analyzer. Melting points were determined using a Kofler micro hot stage. Mass spectra were recorded on a GCMSQP5050A spectrometer made by Shimadzu Company. Chromatographic column parameters were as follows: SPB^ТМ^-5, length 60 m, internal diameter 0.25 mm, thickness of stationary phase film 0.25 μm; injector temperature 250 °C; gas carrier helium; flow rate 0.7 mL/min; detector temperature 250 °C; mass analyzer: quadrupole, electron ionization, electron energy: 70 eV, ion source temperature 200 °C; mass range 34–650 Da. The solvent was chloroform or acetone. Column chromatography was performed on silica gel 60 (70–230 mesh, particle size 0.063–0.200 mm or 230–400 mesh, particle size 0.040–0.063 mm, Merck). Commercially available starting materials were used without further purification. CF_3_/*n*-C_3_F_7_-substituted iminopropargyl alcohols **1** were prepared according to published methods [31–32]. The structures of synthesized products have been proven by ^1^H, ^13^C and 2D (^1^Н,^13^С HMBC) NMR, ^19^F NMR techniques, as well as IR spectra and X-ray diffraction.

**Synthesis of isothiazolium thiocyanates 2 and 4-thiocyanato-2,5-dihydrofuran-2-amines 3. Typical procedure.** A solution of iminopropargyl alcohol **1** (0.5 mmol, 1 equiv) in 3 mL of acetonitrile was quickly added to a mixture of sodium thiocyanate (1 mmol, 2 equiv) and 1 mL of acetic acid. The reaction was carried out at room temperature and with vigorous stirring for 15 minutes. Next, the solvent was removed and the residue was purified using column chromatography (eluting with diethyl ether/hexane 1:8, then acetone/hexane 2:1 to give products **2** and **3**.

**Procedure for oxidation of 5-(2-hydroxypropan-2-yl)-2-phenyl-3-(trifluoromethyl)isothiazol-2-ium thiocyanate (2a).** Hydrogen peroxide (30%, 0.7 mL) was added dropwise to a solution of 5-(2-hydroxypropan-2-yl)-2-phenyl-3-(trifluoromethyl)isothiazol-2-ium thiocyanate (**2a**, 0.09 g, 0.26 mmol) in 0.7 mL acetic acid with vigorous stirring. The reaction mixture was heated in a glycerol bath at 80 °C for 10 h. The solvent was evaporated and the residue was purified by column chromatography (eluting with hexane/acetone 1:1) to obtain the mixture of products **4** and **5**.

## Supporting Information

File 1 Full experimental details, characterization data and copies of NMR spectra for all new compounds.

## Data Availability

All data that supports the findings of this study is available in the published article and/or the supporting information of this article.
