# Supplementary material for: Tandem hydrothiocyanation/cyclization of CF3-iminopropargyl alcohols with NaSCN in the presence of AcOH
Source: Beilstein J Org Chem. 2025 Dec 16;21:2694–702. doi: 10.3762/bjoc.21.207 (PMC12719881; doi:10.3762/bjoc.21.207)

BEILSTEIN JOURNAL OF ORGANIC CHEMISTRY

Supporting Information

for

Tandem hydrothiocyanation/cyclization of CF_3-iminopropargyl alcohols with NaSCN in the presence of AcOH

Ruslan S. Shulgin, Ol'ga G. Volostnykh, Anton V. Stepanov, Igor' A. Ushakov, Alexander V. Vashchenko and Olesya A. Shemyakina

Beilstein J. Org. Chem. **2025,** *21,* 2694–2702. doi:10.3762/bjoc.21.207

Full experimental details, characterization data and copies of NMR spectra for all new compounds

License and Terms: This is a supporting information file under the terms of the Creative Commons Attribution License (https://creativecommons.org/licenses/by/4.0). Please note that the reuse, redistribution and reproduction in particular requires that the author(s) and source are credited and that individual graphics may be subject to special legal provisions.
The license is subject to the *Beilstein Journal of Organic Chemistry* terms and conditions: (https://www.beilstein-journals.org/bjoc/terms)

Table of contents

1. **General information** S3

2. **General procedure for synthesis of isothiazolium thiocyanates 2 and 4-thiocyanato-2,5-dihydrofuran-2-amines 3** S3

3. **Procedure for oxidation of 5-(2-hydroxypropan-2-yl)-2-phenyl-3-(trifluoromethyl)isothiazol-2-ium thiocyanate (2a)** S3

4. **Characterization data for isothiazolium thiocyanates 2** S4

5-(2-Hydroxypropan-2-yl)-2-phenyl-3-(trifluoromethyl)isothiazol-2-ium thiocyanate (**2a**) S4

5-(2-Hydroxybutan-2-yl)-2-phenyl-3-(trifluoromethyl)isothiazol-2-ium thiocyanate (**2b**) S4

5-(1-Hydroxycyclohexyl)-2-phenyl-3-(trifluoromethyl)isothiazol-2-ium thiocyanate (**2c**) S5

5-(1-Hydroxy-1-phenylethyl)-2-phenyl-3-(trifluoromethyl)isothiazol-2-ium thiocyanate (**2d**) S5

5-(Hydroxydiphenylmethyl)-2-phenyl-3-(trifluoromethyl)isothiazol-2-ium thiocyanate (**2e**) S6

2-Butyl-5-(2-hydroxypropan-2-yl)-3-(trifluoromethyl)isothiazol-2-ium thiocyanate (**2h**) S6

5-(2-Hydroxypropan-2-yl)-3-(perfluoropropyl)-2-phenylisothiazol-2-ium thiocyanate (**2i**) S7

5. **Characterization data for 4-thiocyanato-2,5-dihydrofuran-2-amines 3** S7

5,5-Dimethyl-*N*-phenyl-4-thiocyanato-2-(trifluoromethyl)-2,5-dihydrofuran-2-amine (**3a**) S7

5-Ethyl-5-methyl-*N*-phenyl-4-thiocyanato-2-(trifluoromethyl)-2,5-dihydrofuran-2-amine (**3b**) S8

N-Phenyl-4-thiocyanato-2-(trifluoromethyl)-1-oxaspiro[4.5]dec-3-en-2-amine (**3c**) S8

5-Methyl-*N*,5-diphenyl-4-thiocyanato-2-(trifluoromethyl)-2,5-dihydrofuran-2-amine (**3d**) S9

N,5,5-Triphenyl-4-thiocyanato-2-(trifluoromethyl)-2,5-dihydrofuran-2-amine (**3e**) S10

5-Methyl-*N*-phenyl-4-thiocyanato-2-(trifluoromethyl)-2,5-dihydrofuran-2-amine (**3f**) S10

N-Phenyl-4-thiocyanato-2-(trifluoromethyl)-2,5-dihydrofuran-2-amine (**3g**) S11

N-Butyl-5,5-dimethyl-4-thiocyanato-2-(trifluoromethyl)-2,5-dihydrofuran-2-amine (**3h**) S11

5,5-Dimethyl-2-(perfluoropropyl)-*N*-phenyl-4-thiocyanato-2,5-dihydrofuran-2-amine (**3i**) S12

6. **Characterization date for 3-hydroxy-5-(2-hydroxypropan-2-yl)-2-phenyl-3-(trifluoromethyl)-2,3-dihydroisothiazole 1,1-dioxide (4) and 3-hydroperoxy-5-(2-hydroxypropan-2-yl)-2-phenyl-3-(trifluoromethyl)-2,3-dihydroisothiazole 1,1-dioxide (5)** S12

3-Hydroxy-5-(2-hydroxypropan-2-yl)-2-phenyl-3-(trifluoromethyl)-2,3-dihydroisothiazole 1,1-dioxide (**4**) S13

3-Hydroperoxy-5-(2-hydroxypropan-2-yl)-2-phenyl-3-(trifluoromethyl)-2,3-dihydroisothiazole 1,1-dioxide (**5**) S13

7. **X-ray diffraction analysis** S14

8. NMR (^{1}H, ^{13}C, ^{19}F) spectra of the isothiazolium thiocyanates 2 .... S18

5-(2-Hydroxypropan-2-yl)-2-phenyl-3-(trifluoromethyl)isothiazol-2-ium thiocyanate (**2a**) S18

5-(2-Hydroxybutan-2-yl)-2-phenyl-3-(trifluoromethyl)isothiazol-2-ium thiocyanate (**2b**) S19

5-(1-Hydroxycyclohexyl)-2-phenyl-3-(trifluoromethyl)isothiazol-2-ium thiocyanate (**2c**) S21

5-(1-Hydroxy-1-phenylethyl)-2-phenyl-3-(trifluoromethyl)isothiazol-2-ium thiocyanate (**2d**) S22

5-(Hydroxydiphenylmethyl)-2-phenyl-3-(trifluoromethyl)isothiazol-2-ium thiocyanate (**2e**) S24

2-Butyl-5-(2-hydroxypropan-2-yl)-3-(trifluoromethyl)isothiazol-2-ium thiocyanate (**2h**) S25

5-(2-Hydroxypropan-2-yl)-3-(perfluoropropyl)-2-phenylisothiazol-2-ium thiocyanate (**2i**) S27

9. NMR spectra (^{1}H, ^{13}C, ^{19}F) of 4-thiocyanato-2,5-dihydrofuran-2-amines 3 .... S28

5,5-Dimethyl-*N*-phenyl-4-thiocyanato-2-(trifluoromethyl)-2,5-dihydrofuran-2-amine (**3a**) S28

5-Ethyl-5-methyl-*N*-phenyl-4-thiocyanato-2-(trifluoromethyl)-2,5-dihydrofuran-2-amine (**3b**) S30

N-Phenyl-4-thiocyanato-2-(trifluoromethyl)-1-oxaspiro[4.5]dec-3-en-2-amine (**3c**) S31

5-Methyl-*N*,5-diphenyl-4-thiocyanato-2-(trifluoromethyl)-2,5-dihydrofuran-2-amine (**3d**) S33

N,5,5-Triphenyl-4-thiocyanato-2-(trifluoromethyl)-2,5-dihydrofuran-2-amine (**3e**) S34

5-Methyl-*N*-phenyl-4-thiocyanato-2-(trifluoromethyl)-2,5-dihydrofuran-2-amine (**3f**) S36

N-Phenyl-4-thiocyanato-2-(trifluoromethyl)-2,5-dihydrofuran-2-amine (**3g**) S37

N-Butyl-5,5-dimethyl-4-thiocyanato-2-(trifluoromethyl)-2,5-dihydrofuran-2-amine (**3h**) S39

5,5-Dimethyl-2-(perfluoropropyl)-*N*-phenyl-4-thiocyanato-2,5-dihydrofuran-2-amine (**3i**) S40

10. NMR (^{1}H, ^{13}C) spectra of the 3-hydroxy-5-(2-hydroxypropan-2-yl)-2-phenyl-3-(trifluoromethyl)-2,3-dihydroisothiazole 1,1-dioxide (4) S42

1. General information

^1H, ^{13}C {^1H} and ^{19}F NMR spectra were recorded on a Bruker DPX-400 spectrometer (400.1, 100.6 and 376 MHz, respectively) in $CDCl_3$ and $(CD_3)_2CO$ using hexamethyldisiloxane (HMDS) as internal references at 20–25 °C.

IR spectra were measured on a Bruker Vertex-70 instrument in thin layer, films or KBr pellets. Microanalyses were performed on a Flash 2000 elemental analyzer. Melting points were determined using a Kofler micro hot stage. Mass spectra were recorded on a GCMSQP5050A spectrometer made by Shimadzu Company. Chromatographic column parameters were as follows: SPB^{TM}-5, length 60 m, internal diameter 0.25 mm, thickness of stationary phase film 0.25 µm; injector temperature 250 °C, gas carrier helium; flow rate 0.7 mL/min; detector temperature 250 °C; mass analyzer: quadrupole, electron ionization, electron energy: 70 eV, ion source temperature 200 °C; mass range 34–650 Da. The solvent was chloroform or acetone. Column chromatography was performed on silica gel 60 (70–230 mesh, particle size 0.063–0.200 mm or 230–400 mesh, particle size 0.040–0.063 mm, Merck). Commercially available starting materials were used without further purification. CF_3/*n*-C_3F_7-iminopropargylic alcohols 1[1,2] were prepared according to published methods. The structures of synthesized products have been proven by ^1H, ^{13}C and 2D (^1H,^{13}C HMBC) NMR, ^{19}F NMR techniques, as well as by IR spectroscopy and X-ray diffraction.

2. General procedure for synthesis of isothiazolium thiocyanates 2 and 4-thiocyanato-2,5-dihydrofuran-2-amines 3

A solution of iminopropargylic alcohol **1** (0.5 mmol, 1 equiv) in 3 mL of acetonitrile was quickly added to a mixture of sodium thiocyanate (1 mmol, 2 equiv) and 1 mL of acetic acid. The reaction was carried out at room temperature and with vigorous stirring for 15 minutes. Next, the solvent was removed and the residue was purified using column chromatography (eluting with diethyl ether/hexane 1:8, then acetone/hexane 2:1 to give products **2** and **3**.

3. Procedure for oxidation of 5-(2-hydroxypropan-2-yl)-2-phenyl-3-(trifluoromethyl)isothiazol-2-ium thiocyanate (2a)

Hydrogen peroxide (30%, 0.7 mL) was added dropwise to a solution of 5-(2-hydroxypropan-2-yl)-2-phenyl-3-(trifluoromethyl)isothiazol-2-ium thiocyanate (**2a**, 0.09.g, 0.26 mmol) in 0.7 mL acetic acid with vigorous stirring. The reaction mixture was heated in a glycerol bath at 80 °C for 10 h. The solvent was evaporated and the residue was purified by column chromatography (eluting with hexane/acetone 1:1) to obtain the mixture of products **4** and **5**.

---

[1] S. Li, J. Zhu, H. Xie, Z. Chen, Y. Wu, *J. Fluorine. Chem.* **2011**, *132*, 196-201;

[2] T. Schneider, B. Seitz, M. Schiwek, G. Maas, *J. Fluorine. Chem.* **2020**, *235*, 109567.

4. Characterization data for isothiazolium thiocyanates 2

5-(2-Hydroxypropan-2-yl)-2-phenyl-3-(trifluoromethyl)isothiazol-2-ium thiocyanate (2a)

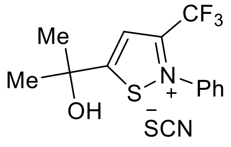

$C_{14}H_{13}F_3N_2OS_2$
346,39

Yield 0.131 g (76%); colorless crystals, m.p. 178-180 ^oC;

IR (KBr): 3106, 2060, 1490, 1460, 1412, 1380, 1266, 1215, 1197, 1142, 969, 767, 721, 690 cm^{-1};

^1H NMR (400.1 MHz, $(CD_3)_2CO$): δ 8.49 (s, 1H, CH), 7.99-7.97 (m, 2H, Ph), 7.84-7.80 (m, 1H, Ph), 7.75-7.72 (m, 2H, Ph), 2.83 (s, 1H, OH), 1.81 (s, 6H, CH_3);

^{13}C {^1H} NMR (100.6 MHz, $(CD_3)_2CO$): δ 196.7 (C-S), 154.5 (q, J_{CF} = 39.6 Hz, CH), 135.3 (Ph), 133.8 (Ph), 133.2 (SCN), 130.9 [Ph (2)], 128.4 [Ph (2)], 119.7 (q, J_{CF} = 2.8 Hz, CF_3), 118.1 (q, J_{CF} = 275.4 Hz, $\underline{C}$-CF_3), 74.3 [$(CH_3)_2\underline{C}$], 30.3 [$(\underline{C}H_3)_2C$];

^{19}F NMR (376 MHz, $(CD_3)_2CO$): δ -60.4 (s, 3F, CF_3);

EIMS, 70 eV, m/z (relative intensity): 289 (4) [M – SCN^{+*}], 272 (44), 229 (15), 209 (17), 172 (13), 115 (35), 77 (71), 59 (38), 51 (100), 43 (44), 39 (23);

Anal. Calcd for $C_{14}H_{13}F_3N_2OS_2$ (346.39): C, 48.55; H, 3.78; F, 16.45; N, 8.09; S, 18.51; found C, 48.42; H, 3.67; F, 16.33; N, 8.13; S, 18.63.

5-(2-Hydroxybutan-2-yl)-2-phenyl-3-(trifluoromethyl)isothiazol-2-ium thiocyanate (2b)

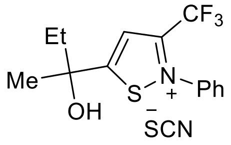

$C_{15}H_{15}F_3N_2OS_2$
360,41

Yield 0.142 g (79%); white crystals, m.p. 130-132 ^oC;

IR (KBr): 3053, 2980, 2064, 1489, 1459, 1410, 1377, 1264, 1215, 1180, 1141, 1029, 928, 851, 765, 720, 689 cm^{-1};

^1H NMR (400.1 MHz, $(CD_3)_2CO$): δ 8.47 (s, 1H, CH), 8.01-7.99 (m, 2H, Ph), 7.83-7.79 (m, 1H, Ph), 7.75-7.71 (m, 2H, Ph), 2.19-2.07 (m, 2H, CH_3-$C\underline{H_2}$), 1.81 (s, 3H, CH_3), 1.00 (t, 3H, $C\underline{H_3}$-CH_2, J = 7.4 Hz);

^{13}C {^1H} NMR (100.6 MHz, $(CD_3)_2CO$): δ 195.6 (C-S), 154.5 (q, CH, J_{CF} = 39.4 Hz), 135.3 (Ph), 133.7 (Ph), 132.9 (SCN), 130.9 [Ph (2)], 128.3 [Ph (2)], 119.7 (q, $\underline{C}$-CF_3, J_{CF} = 2.9 Hz), 118.0 (q, CF_3, J_{CF} = 275 Hz), 77.0 (C-OH), 35.9 (CH_3-$\underline{C}H_2$), 28.1 (CH_3), 8.1 ($\underline{C}H_3$-CH_2);

^{19}F NMR (376 MHz, $(CD_3)_2CO$): δ -60.4 (s, 3F, CF_3);

EIMS, 70 eV, m/z (relative intensity): 302 (3) [M – SCN^{+*}], 288 (13), 272 (44), 252 (14), 238 (14), 229 (48), 184 (18), 159 (69), 134 (58), 116 (37), 96 (17), 77 (100), 59 (36);

Anal. Calcd for $C_{15}H_{15}F_3N_2OS_2$ (360.41): C, 49.99; H, 4.20; F, 15.81; N, 7.77; S, 17.79; found, C, 49.78; H, 4.28; F, 15.90; N, 7.82; S, 17.66.

5-(1-Hydroxycyclohexyl)-2-phenyl-3-(trifluoromethyl)isothiazol-2-ium thiocyanate (2c)

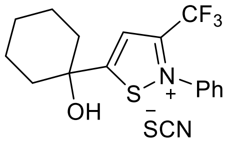

$C_{17}H_{17}F_3N_2OS_2$
386,45

Yield 0.137 g (71%); colorless crystals, m.p. 168-171 ^oC;

IR (KBr): 3078, 3040, 2938, 2873, 2056, 1720, 1489, 1439, 1417, 1356, 1269, 1221, 1196, 1172, 1156, 1030, 1004, 985, 866, 761, 720, 689, 653 cm^{-1};

^{1}H NMR (400.1 MHz, $(CD_3)_2CO$): δ 8.48 (s, 1H, CH), 7.99-7.97 (m, 2H, Ph), 7.83-7.79 (m, 1H, Ph), 7.75-7.72 (m, 2H, Ph), 2.15-1.39 (m, 10H, cyclohexyl);

^{13}C {^{1}H} NMR (100.6 MHz, $(CD_3)_2CO$): δ 198.0 (C-S), 154.4 (q, CH, J_{CF} = 39.4 Hz), 135.2 (Ph), 133.6 (Ph), 133.0 (SCN), 130.8 [Ph (2)], 128.2 [Ph (2)], 119.1 (q, C-CF_3, J_{CF} = 2.7 Hz), 117.8 (q, CF_3, J_{CF} = 275.5 Hz), 75.8 (C-OH), 37.9 [cyclohexyl (2)], 25.1 (cyclohexyl), 21.7 [cyclohexyl (2)];

^{19}F NMR (376 MHz, $(CD_3)_2CO$): δ -60.5 (s, 3F, CF_3);

EIMS, 70 eV, m/z (relative intensity): 328 (1) [M – SCN^{+*}], 299 (13), 284 (26), 256 (35), 229 (21), 160 (34), 77 (100), 69 (19), 59 (39), 55 (41), 51 (42), 41 (32);

Anal. Calcd for $C_{17}H_{17}F_3N_2OS_2$ (386.45): C, 52.84; H, 4.43; F, 14.75; N, 7.25; S, 16.59; found C, 52.72; H, 4.35; F, 14.62; N, 7.38; S, 16.69.

5-(1-Hydroxy-1-phenylethyl)-2-phenyl-3-(trifluoromethyl)isothiazol-2-ium thiocyanate (2d)

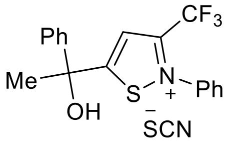

$C_{19}H_{15}F_3N_2OS_2$
408,46

Yield 0.060 g (29%); brown viscous oil;

IR (film): 3064, 2929, 2855, 2058, 1680, 1596, 1489, 1450, 1398, 1377, 1263, 1217, 1158, 1073, 912, 761, 736, 699 cm^{-1};

^{1}H NMR (400.1 MHz, $(CD_3)_2CO$): δ 8.54 (s, 1H, CH), 8.00-7.98 (m, 2H, Ph), 7.83-7.79 (m, 3H, Ph), 7.75-7.71 (m, 2H, Ph), 7.46-7.42 (m, 2H, Ph), 7.39-7.37 (m, 1H, Ph), 2.26 (s, 3H, CH_3);

^{13}C {^{1}H} NMR (100.6 MHz, $(CD_3)_2CO$): δ 194.6 (C-S), 154.8 (q, CH, J_{CF} = 39.6 Hz), 143.5 (Ph), 135.1 (Ph), 133.8 (SCN), 130.9 [Ph (2)], 130.7 (Ph), 129.7 [Ph (2)], 129.3 (Ph), 128.3 [Ph (2)], 126.3 [Ph (2)], 120.3 (q, C-CF_3, J_{CF} = 3 Hz), 117.9 (q, CF_3, J_{CF} = 275.4 Hz), 77.1 (C-OH), 30.1 (CH_3);

^{19}F NMR (376 MHz, $(CD_3)_2CO$): δ -60.4 (s, 3F, CF_3);

Anal. Calcd for $C_{19}H_{15}F_3N_2OS_2$ (408.46): C, 55.87; H, 3.70; F, 13.95; N, 6.86; S, 15.70; found C, 56.02; H, 3.91; F, 14.02; N, 6.96; S, 15.82.

5-(Hydroxydiphenylmethyl)-2-phenyl-3-(trifluoromethyl)isothiazol-2-ium thiocyanate (2e)

2e was prepared from the reaction of **1e** (0.175 g, 0.4 mmol) with NaSCN (0.065 g, 0.8 mmol) and 0.8 mL AcOH according to the general procedure for 30 min.

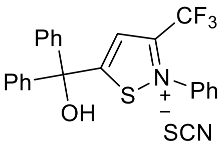

$C_{24}H_{17}F_3N_2OS_2$
470,53

Yield 0.058 g (31%); white crystals, m.p. 120-123 °C;

IR (film): 3360, 3063, 3032, 2923, 2058, 1680, 1601, 1493, 1444, 1395, 1258, 1144, 1025, 909, 754, 733, 701, 648 cm^{-1};

^{1}H NMR (400.1 MHz, $(CD_3)_2CO$): δ 8.55 (s, 1H, CH), 8.05-8.03 (m, 2H, Ph), 7.84-7.80 (m, 1H, Ph), 7.76-7.72 (m, 2H, Ph), 7.63-7.60 (m, 4H, Ph), 7.44-7.42 (m, 6H, Ph);

^{13}C {^{1}H} NMR (100.6 MHz, $(CD_3)_2CO$): δ 192.4 (C-S), 154.7 (q, CH, J_{CF} = 39.1 Hz), 143.1 [Ph (2)], 135.0 (Ph), 133.7 (Ph), 133.5 (SCN), 130.8 [Ph (2)], 129.6 [Ph (2)], 129.4 [Ph (4)], 128.34 [Ph (4)], 128.30 [Ph (2)], 121.7 (q, C-CF_3, J_{CF} = 2.9 Hz), 117.7 (q, CF_3, J_{CF} = 275.7 Hz), 82.1 (C-OH);

^{19}F NMR (376 MHz, $(CD_3)_2CO$): δ -60.2 (s, 3F, CF_3);

Anal. Calcd for $C_{24}H_{17}F_3N_2OS_2$ (470.53): C, 61.26; H, 3.64; F, 12.11; N, 5.95; S, 13.63; found C, 61.37; H, 3.72; F, 12.19; N, 6.03; S, 13.72.

2-Butyl-5-(2-hydroxypropan-2-yl)-3-(trifluoromethyl)isothiazol-2-ium thiocyanate (2h)

2h was prepared from the reaction of **1h** (0.111 g, 0.47 mmol) with NaSCN (0.076 g, 0.94 mmol) and 1 mL AcOH according to the general procedure for 25 min.

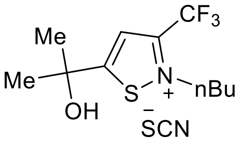

$C_{12}H_{17}F_3N_2OS_2$
326,40

Yield 0.091 g (59%); colorless crystals, m.p. 111-113 °C;

IR (film): 3217, 3093, 2967, 2935, 2876, 2071, 1466, 1414, 1382, 1260, 1204, 1163, 1099 cm^{-1};

^{1}H NMR (400.1 MHz, $(CD_3)_2CO$): δ 8.30 (s, 1H, CH), 4.86 (t, 2H, CH_3-CH_2-CH_2-C<u>H</u>$_2$, J = 7.7 Hz), 2.19 (p, 2H, CH_3-CH_2-C$\underline{H_2}$-CH_2, J = 7.7 Hz), 1.76 (s, 6H, $(CH_3)_2C$), 1.61-1.52 (m, 2H, CH_3-C$\underline{H_2}$-CH_2-CH_2), 1.00 (t, 3H, C$\underline{H_3}$-CH_2-CH_2-CH_2 J = 7.6 Hz);

^{13}C {^{1}H} NMR (100.6 MHz, $(CD_3)_2CO$): δ 193.3 (C-S), 153.5 (q, CH, J_{CF} = 39.7 Hz), 133.2 (SCN), 119.3 (q, $\underline{C}$-CF_3, J_{CF} = 3.2 Hz), 118.1 (q, CF_3, J_{CF} = 275.4 Hz), 74.0 [$(CH_3)_2\underline{C}$], 55.2 (q, CH_3-CH_2-CH_2-$\underline{C}H_2$, J_{CF} = 2.1 Hz), 33.8 (CH_3-CH_2-$\underline{C}H_2$-CH_2), 30.4 [$(\underline{C}H_3)_2C$], 20.1 (CH_3-$\underline{C}H_2$-CH_2-CH_2), 13.6 ($\underline{C}H_3$-CH_2-CH_2-CH_2);

^{19}F NMR (376 MHz, $(CD_3)_2CO$): δ -61.1 (s, 3F, CF_3);

EIMS, 70 eV, m/z (relative intensity): 268 (3) [M – SCN^{+*}], 251 (11), 196 (97), 166 (12), 146 (7), 89 (31), 58 (33), 43 (100);

Anal. Calcd for $C_{12}H_{17}F_3N_2OS_2$ (326.40): C, 44.16; H, 5.25; F, 17.46; N, 8.58; S, 19.64; found, C, 44.30; H, 5.34; F, 17.55; N, 8.66; S, 19.77.

5-(2-Hydroxypropan-2-yl)-3-(perfluoropropyl)-2-phenylisothiazol-2-ium thiocyanate (2i)

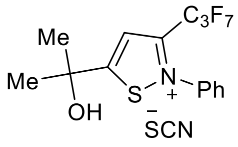

$C_{16}H_{13}F_7N_2OS_2$
446,40

Yield 0.168 g (75%); brown viscous oil;

IR (thin layer): 3071, 2985, 2934, 2060, 1711, 1638, 1488, 1452, 1379, 1344, 1237, 1140, 1120, 1030, 950, 921, 818, 766, 735, 692 cm^{-1};

^{1}H NMR (400.1 MHz, $(CD_3)_2CO$): δ 8.54 (s, 1H, CH), 8.05-8.03 (m, 2H, Ph), 7.82-7.79 (m, 1H, Ph), 7.74-7.70 (m, 2H, Ph), 1.83 (s, 6H, $(CH_3)_2C$);

^{13}C {^{1}H} NMR (100.6 MHz, $(CD_3)_2CO$): δ 196.0 (C-S), 153.0 (t, CH, J_{CF} = 28.7 Hz), 135.3 (Ph), 133.6 (Ph), 133.1 (SCN), 130.4 [Ph (2)], 128.7 [Ph (2)], 121.3 (t, $\underline{C}$-CF_2-CF_2-CF_3, J_{CF} = 4.4 Hz), 105.3-119.7 (m, C_3F_7), 70.3 [$(CH_3)_2\underline{C}$], 30.2 [$(\underline{C}H_3)_2C$];

^{19}F NMR (376 MHz, $(CD_3)_2CO$): δ -80.6 (t, 3F, CF_2-CF_2-$C\underline{F_3}$, J = 10.3 Hz), -106.7 (m, 2F, CF_2-$C\underline{F_2}$-CF_3), -124.3 (t, 2F, $C\underline{F_2}$-CF_2-CF_3, J = 10.6 Hz);

EIMS, 70 eV, m/z (relative intensity): 388 [M-SCN^{+*}], 372 (56), 330 (34), 210 (46), 160 (37), 77 (100), 59 (64), 51 (42), 43 (57);

Anal. Calcd for $C_{16}H_{13}F_7N_2OS_2$ (446.40): C, 43.05; H, 2.94; F, 29.79; N, 6.28; S, 14.36; found, C. 43.19; H, 3.03; F, 29.88; N, 6.20; S, 14.48.

5. Characterization data for 4-thiocyanato-2,5-dihydrofuran-2-amines 3

5,5-Dimethyl-*N*-phenyl-4-thiocyanato-2-(trifluoromethyl)-2,5-dihydrofuran-2-amine (3a)

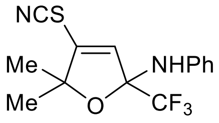

$C_{14}H_{13}F_3N_2OS$
314,33

Yield 0.037 g (23%); yellow oil;

IR (thin layer): 3340, 3094, 3058, 2982, 2934, 2168, 1631, 1601, 1497, 1321, 1286, 1239, 1164, 1070, 1029, 985, 932, 886, 845, 762, 694 cm^{-1};

^{1}H NMR (400.1 MHz, $CDCl_3$): δ 7.31-7.27 (m, 2H, Ph), 7.14-7.10 (m, 1H, Ph), 6.97-6.95 (m, 2H, Ph), 6.11 (s, 1H, CH), 4.16 (s, 1H, NH), 1.39 (s, 3H, CH_3), 0.80 (s, 3H, CH_3);

^{13}C {^{1}H} NMR (100.6 MHz, CDCl$_3$): δ 140.9 (Ph), 139.8 ($\underline{C}$-SCN), 128.9 [Ph (2)], 125.2 [Ph (2)], 124.8 (Ph), 122.5 (CH), 122.4 (q, CF$_3$, J_{CF} = 285.9 Hz), 106.9 (SCN), 99.3 (q, $\underline{C}$-CF$_3$, J_{CF} = 31.2 Hz), 89.8 [(CH$_3$)$_2\underline{C}$], 26.52 (CH$_3$), 26.46 (CH$_3$);

^{19}F NMR (376 MHz, CDCl$_3$): δ -80.9 (s, 3F, CF$_3$);

EIMS, 70 eV, m/z (relative intensity): 314 (11) [M$^+$], 245 (24), 222 (100), 195 (47), 164 (31), 135 (11), 113 (14), 93 (20), 77 (30), 65 (33), 51 (18), 43 (25), 39 (28);

Anal. Calcd for $C_{14}H_{13}F_3N_2OS$ (314.33): C, 53.50; H, 4.17; F, 18.13; N, 8.91; S, 10.20; found C, 53.47; H, 4.20; F, 18.15; N, 8.88; S, 10.22.

5-Ethyl-5-methyl-*N*-phenyl-4-thiocyanato-2-(trifluoromethyl)-2,5-dihydrofuran-2-amine (3b)

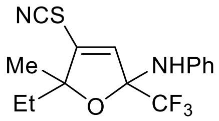

$C_{15}H_{15}F_3N_2OS$
328,35

Yield 0.034 g (21%); yellow oil. The doubling of all the NMR signals resulted from of the two diastereomers (ratio 1:1);

IR (film): 3348, 3093, 3058, 2978, 2937, 2884, 2854, 2168, 1629, 1601, 1497, 1456, 1379, 1278, 1241, 1180, 1070, 1025, 954, 883, 755, 696 cm^{-1};

^{1}H NMR (400.1 MHz, CDCl$_3$): δ 7.28-7.22 (m, 4H, Ph), 7.10-7.05 (m, 2H, Ph), 6.95-6.92 (m, 4H, Ph), 6.07 [6.05] (s, 1H, CH), 4.13 (s, 1H, NH), 1.66-1.49 [1.25-1.10] (m, 2H, CH$_3$-C$\underline{H_2}$), 1.32 [0.75] (s, 3H, CH$_3$), 0.94 [0.70] (t, 3H, C$\underline{H_3}$-CH$_2$, J = 7.4 Hz);

^{13}C {^{1}H} NMR (100.6 MHz, CDCl$_3$): δ 141.1 [141.2] (Ph), 140.0 [140.3] ($\underline{C}$-SCN), 128.93 [128.97] [Ph (2)], 124.36 [124.7] [Ph (2)], 124.34 [124.5] (Ph), 122.6 [122.7] (q, CF$_3$, J_{CF} = 286.1 Hz), 121.8 [122.0] (CH), 107.02 [107.04] (SCN), 99.0 [99.1] (q, $\underline{C}$-CF$_3$, J_{CF} = 31.4 Hz), 92.1 [92.4] (C-Et), 32.1 [32.3] (CH$_3$), 23.1 [23.2] (CH$_3$-$\underline{C}$H$_2$), 7.6 [7.9] ($\underline{C}$H$_3$-CH$_2$);

^{19}F NMR (376 MHz, CDCl$_3$): δ -80.3 [-80.4] (s, 3F, CF$_3$);

EIMS, 70 eV, m/z (relative intensity): 328 (9) [M$^+$], 259 (10), 236 (69), 209 (36), 177 (19), 163 (10), 93 (19), 77 (26), 65 (22), 51 (13), 43 (100);

Anal. Calcd for $C_{15}H_{15}F_3N_2OS$ (328.35): C, 54.87; H, 4.60; F, 17.36; N, 8.53; S, 9.76; found C, 54.81; H, 4.63; F, 17.31; N, 8.55; S, 9.72.

***N*-Phenyl-4-thiocyanato-2-(trifluoromethyl)-1-oxaspiro[4.5]dec-3-en-2-amine (3c)**

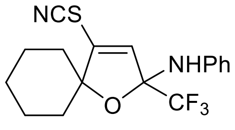

$C_{17}H_{17}F_3N_2OS$
354,39

Yield 0.026 g (15%); white crystals, m.p. 73-75 °C;

IR (KBr): 3342, 3093, 3057, 2935, 2860, 2168, 1628, 1600, 1497, 1447, 1283, 1239, 1163, 1063, 1019, 969, 907, 864, 843, 763, 694 cm^{-1};

^{1}H NMR (400.1 MHz, $CDCl_3$): δ 7.26-7.23 (m, 2H, Ph), 7.10-7.07 (m, 1H, Ph), 6.94-6.92 (m, 2H, Ph), 6.04 (s, 1H, CH), 4.13 (s, 1H, NH), 1.70-0.98 (m, 10H, cyclohexyl);

^{13}C {^{1}H} NMR (100.6 MHz, $CDCl_3$): δ 141.1 (Ph), 140.0 (C-SCN), 128.9 [Ph (2)], 125.4 [Ph (2)], 124.8 (Ph), 122.7 (q, CF_3, J_{CF} = 286 Hz), 121.6 (CH), 107.2 (SCN), 99.3 (q, C-CF_3, J_{CF} = 31.1 Hz), 90.9 [$(CH_2)_5$C], 34.9 (cyclohexyl), 34.7 (cyclohexyl), 24.6 (cyclohexyl), 21.8 (cyclohexyl), 21.4 (cyclohexyl);

^{19}F NMR (376 MHz, $CDCl_3$): δ -80.9 (s, 3F, CF_3);

EIMS, 70 eV, m/z (relative intensity): 354 (10) [M^+], 285 (9), 262 (69), 235 (100), 203 (16), 93 (26), 77 (32), 65 (10).

Anal. Calcd for $C_{17}H_{17}F_3N_2OS$ (354.39): C, 57.62; H, 4.84; F, 16.08; N, 7.90; S, 9.05; found C, 57.55; H, 4.79; F, 16.10; N, 7.86; S, 9.11.

5-Methyl-*N*,5-diphenyl-4-thiocyanato-2-(trifluoromethyl)-2,5-dihydrofuran-2-amine (3d)

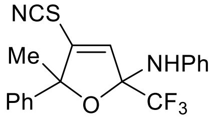

$C_{19}H_{15}F_3N_2OS$
376,40

Yield 0.047 g (25%); yellow oil. The doubling of all the NMR signals resulted from of the two diastereomers (ratio 1:1);

IR (thin layer): 3369, 3092, 3061, 3030, 2984, 2928, 2854, 2169, 1727, 1628, 1599, 1497, 1448, 1377, 1273, 1241, 1273, 1241, 1170, 1062, 1025, 956, 895, 861, 763, 698 cm^{-1};

^{1}H NMR (400.1 MHz, $CDCl_3$): δ 7.35-7.28 (m, 6H, Ph), 7.21-7.08 (m, 7H, Ph), 7.03-7.01 (m, 3H, Ph), 6.93-6.91 (m, 2H, Ph), 6.84-6.82 (m, 2H, Ph), 6.23 [6.28] (s, 1H, CH), 4.27 [4.24] (s, 1H, NH), 1.85 [1.23] (s, 3H, CH_3);

^{13}C {^{1}H} NMR (100.6 MHz, $CDCl_3$): δ 141.2 [141.1] (Ph), 140.6 [140.0] (C-SCN), 139.26 [139.22] (Ph), 129.2 [129.1] [Ph (2)], 128.9 [128.8] [Ph (2)], 128.72 [128.68] [Ph (2)], 125.8 [125.7] [Ph (2)], 124.4 [124.1] (Ph), 123.8 [123.5] (Ph), 122.8 [122.7] (q, CF_3, J_{CF} = 287.2 Hz), 123.2 [122.3] (CH), 107.2 [107.0] (SCN), 99.4 [99.3] (q, C-CF_3, J_{CF} = 31.6 Hz), 92.6 [92.5] (C-Ph), 26.2 [25.4] (CH_3);

^{19}F NMR (376 MHz, $CDCl_3$): δ -79.5 [-78.8] (s, 3F, CF_3);

EIMS, 70 eV, m/z (relative intensity): 376 (9) [M^+], 307 (17), 284 (56), 257 (22), 226 (71), 213 (42), 197 (11), 160 (32), 128 (85), 93 (46), 77 (100), 65 (68), 51 (58), 39 (36);

Anal. Calcd for $C_{19}H_{15}F_3N_2OS$ (376.40): C, 60.63; H, 4.02; F, 15.14; N, 7.44; S, 8.52; found C, 60.59; H, 4.07; F, 15.10; N, 7.39; S, 8.53.

_N_,5,5-Triphenyl-4-thiocyanato-2-(trifluoromethyl)-2,5-dihydrofuran-2-amine (3e)

3e was prepared from the reaction of **1e** (0.175 g, 0.4 mmol) with NaSCN (0.065 g, 0.8 mmol) and 0.8 mL AcOH according to the general procedure for 30 min.

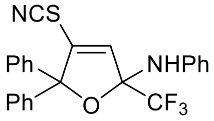

$C_{24}H_{17}F_3N_2OS$
438,47

Yield 0.030 g (17%); white crystals, m.p. 93-95 ^oC;

IR (KBr): 3349, 3096, 3057, 3031, 2168, 1625, 1597, 1494, 1446, 1277, 1236, 1179, 1154, 1049, 999, 923, 893, 763, 729, 696 cm^{-1};

^{1}H NMR (400.1 MHz, $CDCl_3$): δ 7.34-7.32 (m, 3H, Ph), 7.26-7.23 (m, 2H, Ph), 7.19-7.16 (m, 1H, Ph), 7.13-7.09 (m, 2H, Ph), 7.07-7.04 (m, 2H, Ph), 7.01-6.97 (m, 1H, Ph), 6.81-6.79 (m, 2H, Ph), 6.75-6.73 (m, 2H, Ph), 6.43 (s, 1H, CH), 4.26 (s, 1H, NH);

^{13}C {^{1}H} NMR (100.6 MHz, $CDCl_3$): δ 141.2 (Ph), 140.8 (Ph), 140.2 (Ph), 139.3 (C-SCN), 129.2 [Ph (2)], 128.8 (Ph), 128.5 [Ph (2)], 128.4 [Ph (2)], 128.3 [Ph (2)], 127.3 [Ph (2)], 127.2 [Ph (2)], 123.8 (Ph), 123.5 (Ph), 122.9 (CH), 122.7 (q, CF_3, J_{CF} = 287.4 Hz), 107.4 (SCN), 99.4 (q, C-CF_3, J_{CF} = 31.8 Hz), 96.4 [$(Ph)_2C$];

^{19}F NMR (376 MHz, $CDCl_3$): δ -78.4 (s, 3F, CF_3);

EIMS, 70 eV, m/z (relative intensity): 438 (25) [M^+], 346 (46), 319 (52), 299 (26), 288 (72), 271 (25), 250 (51), 233 (22), 221 (42), 191 (100), 165 (28), 105 (13), 92 (14), 77 (52), 65 (28), 51 (25), 39 (18);

Anal. Calcd for $C_{24}H_{17}F_3N_2OS$ (438.47): C, 65.74; H, 3.91; F, 13.00; N, 6.39; S, 7.31; found C, 65.79; H, 3.96; F, 12.93; N, 6.45; S, 7.38.

5-Methyl-_N_-phenyl-4-thiocyanato-2-(trifluoromethyl)-2,5-dihydrofuran-2-amine (3f)

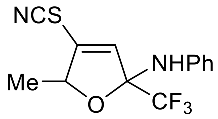

$C_{13}H_{11}F_3N_2OS$
300,30

Yield 0.069 g (46%); yellow oil. The doubling of all the NMR signals resulted from of the two diastereomers (ratio 1:1.25);

IR (film): 3339, 3093, 3059, 2984, 2932, 2885, 2167, 1626, 1602, 1497, 1274, 1163, 1055, 1025, 948, 915, 755, 724, 697 cm^{-1};

^{1}H NMR (400.1 MHz, $CDCl_3$): δ 7.27-7.22 (m, 4H, Ph), 7.10-7.04 (m, 2H, Ph), 6.92-6.89 (m, 4H, Ph), 6.09 [6.03] (d, 1H, CH, J = 1.9 Hz), 4.91 [4.80] (qd, 1H, H-C-CH_3, J = 6.5, 1.9 Hz), 4.21 [4.27] (s, 1H, NH), 1.00 [1.40] (d, 3H, CH_3, J = 6.5 Hz);

^{13}C {^{1}H} NMR (100.6 MHz, $CDCl_3$): δ 141.1 [140.9] (Ph), 136.1 [136.0] (C̲-SCN), 129.2 [129.0] [Ph (2)], 124.8 [124.2] (Ph), 124.5 [124.4] [Ph (2)], 123.1 (CH), 122.8 [122.3] (q, CF_3, J_{CF} = 285.5 Hz), 106.5 [106.3] (SCN), 100.2 [100.0] (q, C̲-CF_3, J_{CF} = 31.1 Hz), 83.0 [83.6] (H-C̲-CH_3), 19.4 [19.7] (CH_3);

^{19}F NMR (376 MHz, $CDCl_3$): δ -81.7 [-81.0] (s, 3F, CF_3);

EIMS, 70 eV, m/z (relative intensity): 300 (5) [M^+], 231 (8), 208 (13), 160 (8), 150 (13), 93 (100), 77 (13), 66 (11), 51 (9);

Anal. Calcd for $C_{13}H_{11}F_3N_2OS$ (300.30): C, 52.00; H, 3.69; F, 18.98; N, 9.33; S, 10.68; found C, 51.94; H, 3.59; F, 18.92; N, 9.36; S, 10.62.

***N*-Phenyl-4-thiocyanato-2-(trifluoromethyl)-2,5-dihydrofuran-2-amine (3g)**

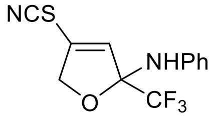

$C_{12}H_9F_3N_2OS$
286,27

Yield 0.063 g (44%); yellow oil;

IR (film): 3386, 3095, 3060, 2925, 2873, 2167, 1624, 1603, 1499, 1276, 1179, 1086, 1049, 1031, 970, 755, 723 cm^{-1};

^{1}H NMR (400.1 MHz, $CDCl_3$): δ 7.26-7.22 (m, 2H, Ph), 7.06-7.03 (m, 1H, Ph), 6.88-6.86 (m, 2H, Ph), 6.00 (s, 1H, CH), 4.79 (d, 1H, CH_2, *J* = 12.7 Hz), 4.70 (d, 1H, CH_2, *J* = 12.7 Hz), 4.37 (s, 1H, NH);

^{13}C {^{1}H} NMR (100.6 MHz, $CDCl_3$): δ 141.0 (Ph), 131.7 (C̲-SCN), 129.3 [Ph (2)], 124.7 [Ph (2)], 123.9 (Ph), 122.9 (q, CF_3, J_{CF} = 287 Hz), 122.2 (CH), 106.0 (SCN), 101.6 (q, C̲-CF_3, J_{CF} = 31.5 Hz), 76.4 (CH_2);

^{19}F NMR (376 MHz, $CDCl_3$): δ -81.7 (s, 3F, CF_3);

EIMS, 70 eV, m/z (relative intensity): 286 (3) [M^+], 243 (5), 217 (5), 136 (5), 93 (100), 77 (15), 65 (14), 51 (10), 39 (12);

Anal. Calcd for $C_{12}H_9F_3N_2OS$ (286.27): C, 50.35; H, 3.17; F, 19.91; N, 9.79; S, 11.20; found C, 50.30; H, 3.12; F, 19.85; N, 9.83; S, 11.26.

***N*-Butyl-5,5-dimethyl-4-thiocyanato-2-(trifluoromethyl)-2,5-dihydrofuran-2-amine (3h)**

3h was prepared from the reaction of **1h** (0.111 g, 0.47 mmol) with NaSCN (0.076 g, 0.94 mmol) and 1 mL AcOH according to the general procedure for 25 min.

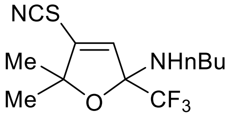

$C_{12}H_{17}F_3N_2OS$
294,34

Yield 0.027 g (19%); yellow oil;

IR (film): 3351, 2962, 2934, 2871, 2168, 1630, 1463, 1296, 1172, 1059, 1059, 982, 920, 888 cm^{-1};

^{1}H NMR (400.1 MHz, CDCl$_3$): δ 5.84 (s, 1H, CH), 2.73-2.67 (m, 1H, CH_3-CH_2-CH_2-$C\underline{H_2}$), 2.49-2.42 (m, 1H, CH_3-CH_2-CH_2-$C\underline{H_2}$), 1.88 (s, 1H, NH), 1.49 (s, 3H, CH_3), 1.46 (s, 3H, CH_3), 1.52-1.42 (m, 2H, CH_3-CH_2-$C\underline{H_2}$-CH_2), 1.40-1.31 (m, 2H, CH_3-$C\underline{H_2}$-CH_2-CH_2), 0.91 (t, 3H, $C\underline{H_3}$-CH_2-CH_2-CH_2, J = 7.2 Hz);

^{13}C {^{1}H} NMR (100.6 MHz, CDCl$_3$): δ 139.3 ($\underline{C}$-SCN), 122.9 (CH), 122.5 (q, CF_3, J_{CF} = 285.6 Hz), 107.2 (SCN), 99.8 (q, $\underline{C}$-CF_3, J_{CF} = 30.8 Hz), 89.1 [$(CH_3)_2\underline{C}$], 41.7 (CH_3-CH_2-CH_2-$\underline{C}H_2$), 32.4 (CH_3-CH_2-$\underline{C}H_2$-CH_2), 27.5 (CH_3), 26.7 (CH_3), 20.4 (CH_3-$\underline{C}H_2$-CH_2-CH_2), 14.0 ($\underline{C}H_3$-CH_2-CH_2-CH_2);

^{19}F NMR (376 MHz, CDCl$_3$): δ -80.8 (s, 3F, CF_3);

EIMS, 70 eV, m/z (relative intensity): 279 (3) [M – CH_3^{+*}], 225 (100), 222 (76), 195 (29), 178 (10), 164 (22), 142 (9), 113 (10), 95 (9), 69 (16), 57 (36), 41 (51);

5,5-Dimethyl-2-(perfluoropropyl)-*N*-phenyl-4-thiocyanato-2,5-dihydrofuran-2-amine (3i)

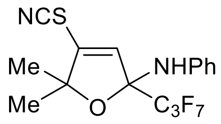

$C_{16}H_{13}F_7N_2OS$
414,34

Yield 0.037 g (18%); yellow oil;

IR (thin layer): 3336, 3100, 3059, 2981, 2932, 2855, 2168, 1631, 1600, 1497, 1463, 1435, 1345, 1284, 1228, 1153, 1120, 1066, 985, 943, 901, 844, 761, 734, 695 cm^{-1};

^{1}H NMR (400.1 MHz, CDCl$_3$): δ 7.29-7.25 (m, 2H, Ph), 7.13-7.09 (m, 1H, Ph), 6.94-6.93 (m, 2H, Ph), 6.09 (s, 1H, CH), 4.07 (s, 1H, NH), 1.36 (s, 3H, CH_3), 0.77 (s, 3H, CH_3);

^{13}C {^{1}H} NMR (100.6 MHz, CDCl$_3$): δ 140.6 (Ph), 139.7 ($\underline{C}$-SCN), 129.0 [Ph (2)], 125.7 [Ph (2)], 125.1 (Ph), 122.9 (CH), 106.6-119.7 (m, C_3F_7), 106.9 (SCN), 100.9 (t, $\underline{C}$-CF_2-CF_2-CF_3, J_{CF} = 24.8 Hz), 89.2 [$(CH_3)_2\underline{C}$], 26.9 (CH_3), 26.4 (CH_3);

^{19}F NMR (376 MHz, CDCl$_3$): δ -80.5 (t, 3F, J_{CF} = 10.5 Hz), -117.7 (d, 1F, J = 280.7 Hz), -121.4 (d, 1F, J = 290.8 Hz), -123.2 (d, 1F, J = 280.7Hz), -125.4 (d, 1F, J = 290.8 Hz);

EIMS, 70 eV, m/z (relative intensity): 414 (5) [M^+], 322 (100), 295 (27), 263 (15), 245 (49), 153 (28), 145 (33), 93 (13), 85 (11), 77 (31), 69 (15), 65 (30), 39 (24).

Anal. Calcd for $C_{16}H_{13}F_7N_2OS$ (414.34): C, 46.38; H, 3.16; F, 32.10; N, 6.76; S, 7.74; found C, 46.43; H, 3.20; F, 32.05; N, 6.71; S, 7.69.

6. Characterization date for 3-hydroxy-5-(2-hydroxypropan-2-yl)-2-phenyl-3-(trifluoromethyl)-2,3-dihydroisothiazole 1,1-dioxide (4) and 3-hydroperoxy-5-(2-hydroxypropan-2-yl)-2-phenyl-3-(trifluoromethyl)-2,3-dihydroisothiazole 1,1-dioxide (5)

3-Hydroxy-5-(2-hydroxypropan-2-yl)-2-phenyl-3-(trifluoromethyl)-2,3-dihydroisothiazole 1,1-dioxide (4)

A mixture of products **4** and **5** in a ratio of 4:1, respectively.

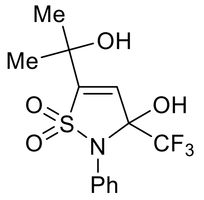

$C_{13}H_{14}F_3NO_4S$
337,31

Yield 0.060 g (67%), white crystals, m.p. 145-147 °C.

IR (film): 3396, 3075, 2987, 2935, 1493, 1299, 1262, 1187, 1114, 1046, 966, 697 cm^{-1};

^{1}H NMR (400.1 MHz, $(CD_3)_2CO$): δ 7.52-7.46 (m, 5H, Ph), 6.87 (s, 1H, CH), 4.94 (s, 1H, CF_3-C-OH), 3.20 (s, 1H, C-OH), 1.63 (s, 3H, CH_3), 1.60 (s, 3H, CH_3);

^{13}C {^{1}H} NMR (100.6 MHz, $(CD_3)_2CO$): δ 154.8 (C-S), 133.2 [Ph (2)], 131.8 (Ph), 130.2 (Ph), 129.8 [Ph (2)], 126.2 (CH), 123.4 (q, CF_3, J_{CF} = 287 Hz), 85.4 (q, C-CF_3, J_{CF} = 33.6 Hz), 70.8 [$(CH_3)_2$C], 30.1 [(CH_3)$_2$C];

EIMS, 70 eV, m/z (relative intensity): 337 (28) [M$^+$], 304 (11), 268 (28), 250 (58), 210 (24), 144 (34), 120 (29), 92 (38), 77 (100), 69 (19), 59 (35), 43 (91).

3-Hydroperoxy-5-(2-hydroxypropan-2-yl)-2-phenyl-3-(trifluoromethyl)-2,3-dihydroisothiazole 1,1-dioxide (5)

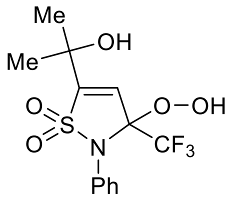

$C_{13}H_{14}F_3NO_5S$
353,31

^{1}H NMR (400.1 MHz, $(CD_3)_2CO$): δ 12.32 (s, 1H, O-OH), 7.52-7.46 (m, 5H, Ph), 6.86 (s, 1H, CH), 5.01 (s, 1H, C-OH), 1.65 (s, 3H, CH_3), 1.63 (s, 3H, CH_3);

^{13}C {^{1}H} NMR (100.6 MHz, $(CD_3)_2CO$): δ 157.0 (C-S), 132.8 [Ph (2)], 131.1 (Ph), 130.5 (Ph), 130.1 [Ph (2)], 123.7 (CH), 122.1 (q, CF_3, J_{CF} = 288.1 Hz), 93.3 (q, C-CF_3, J_{CF} = 31.8 Hz), 71.1 [$(CH_3)_2$C], 30.3 [(CH_3)$_2$C].

7. X-ray diffraction analysis

The determination of the unit cell and the data collection for 5-(2-hydroxypropan-2-yl)-2-phenyl-3-(trifluoromethyl)isothiazol-2-ium thiocyanate was performed on a Bruker D8 VENTURE PHOTON 100 CMOS diffractometer with Mo K_{α} radiation (λ = 0.71073) at 293.2(2) K using the ω–φ scan technique. A specimen of $C_{28}H_{26}N_4F_6O_2S_4$, approximate dimensions 0.60 mm × 0.60 mm × 0.45 mm, was used for the X-ray crystallographic analysis. The X-ray intensity data were measured. The integration of the data using an orthorhombic unit cell with *Pca*2_1 space group yielded a total of 66222 reflections to a maximum 2θ angle of 53.0°, of which 6761 were independent (completeness = 99.9%, R_{int} = 7.43%, R_{sig} = 4.16%) and 4698 were greater than 2σ(F2). The final cell constants of *a* = 11.513(2) Å, *b* = 11.513(2) Å, *c* = 24.721(5) Å, *Z* = 4, volume = 3276.9(10) Å^3. The calculated minimum and maximum transmission coefficients (based on crystal size) are 0.808 and 0.852. All H atoms were treated by mixed method.

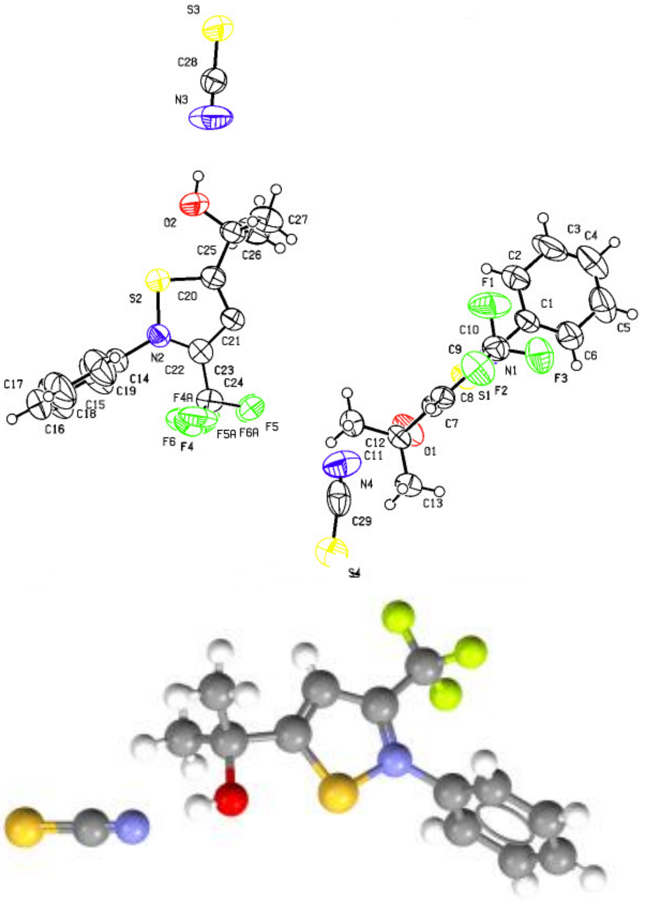

Figure 1: X-ray structure of 5-(2-hydroxypropan-2-yl)-2-phenyl-3-(trifluoromethyl)isothiazol-2-ium thiocyanate **2a**. Thermal ellipsoids are set at 50% probability.

The final anisotropic full-matrix least-squares refinement on F2 with 450 variables converged at R1 = 4.49%, for the observed data and wR2 = 12.19% for all data. The goodness-of-fit was 1.02. The largest peak in the final difference electron density synthesis was 0.26 e−/Å^3 and the largest hole was −0.28 e−/Å^3. On the basis of the final model, the calculated density was 1.402 g/cm^3 and F(000), 1420 e−.

Data were corrected for absorption effects using the multi-scan method (SADABS)[3]. The structure was solved using the Bruker SHELXTL Software Package[4] and refined using Olex2[5] package.

Atomic coordinates, bond lengths, bond angles and thermal parameters have been deposited at the Cambridge Crystallographic Data Centre (CCDC) and allocated the deposition numbers CCDC **2474219**. These data can be obtained free of charge from the Cambridge Crystallographic Data Centre via www.ccdc.cam.ac.uk/data_request/cif.

Table 1. Bond lengths for 5-(2-hydroxypropan-2-yl)-2-phenyl-3-(trifluoromethyl)isothiazol-2-ium thiocyanate **2a**.

Atom	Atom	Length/Å	Atom	Atom	Length/Å
S1	N1	1.662(4)	C5	C6	1.519(7)
S1	C15	1.673(5)	C5	C7	1.512(7)
S2	C14	1.674(5)	C5	O1	1.425(6)
S2	N15	1.664(4)	C8	C9	1.501(7)
S3	C4	1.606(6)	C10	C11	1.398(9)
S4	C28	1.638(9)	O12	C13	1.425(7)
F1	C9	1.308(7)	C13	C14	1.503(7)
F2	C9	1.309(7)	C13	C20	1.508(8)
F3	C9	1.328(7)	C13	C26	1.503(8)
F4	C25	1.35(2)	C14	C24	1.364(7)
F5	C25	1.319(19)	N15	C16	1.461(6)
F6	C25	1.28(2)	N15	C23	1.332(6)
N1	C12	1.457(6)	C16	C17	1.325(9)
N1	C8	1.340(6)	C16	C21	1.343(9)
C15	C27	1.360(7)	C17	C18	1.399(11)
C15	C5	1.518(6)	C18	C19	1.347(13)
C27	C8	1.381(6)	C19	C22	1.308(12)
C4	N27	1.125(7)	C21	C22	1.385(10)
N5	C28	1.023(9)	C23	C24	1.379(7)
C1	C2	1.345(11)	C23	C25	1.503(7)
C1	C10	1.346(11)	C25	F5A	1.29(2)
C2	C3	1.397(9)	C25	F6A	1.296(19)
C3	C12	1.342(8)	C25	F4A	1.34(2)
C12	C11	1.355(8)			

[3] G.M. Sheldrick. **2016**, SADABS, Version 2016/2. Bruker AXS Inc., Germany.
[4] G.M. Sheldrick, *Acta Crystallogr*. **2008**, *D64*, 112 – 122.
[5] O.V. Dolomanov, L.J. Bourhis, R.J. Gildea, J.A.K. Howard, H. Puschmann, *J. Appl. Cryst.* **2009**, *42*, 339–341.

Table 2. Bond angles for 5-(2-hydroxypropan-2-yl)-2-phenyl-3-(trifluoromethyl)isothiazol-2-ium thiocyanate **2a**.

**Atom**	**Atom**	**Atom**	**Angle/˚**	**Atom**	**Atom**	**Atom**	**Angle/˚**
N1	S1	C15	91.6(2)	O12	C13	C26	111.4(5)
N15	S2	C14	92.1(2)	C14	C13	C20	111.5(5)
C12	N1	S1	118.7(4)	C14	C13	C26	109.7(4)
C8	N1	S1	112.1(3)	C26	C13	C20	111.7(5)
C8	N1	C12	129.2(4)	C13	C14	S2	116.6(4)
C27	C15	S1	112.4(4)	C24	C14	S2	111.4(4)
C27	C15	C5	132.7(4)	C24	C14	C13	131.9(4)
C5	C15	S1	114.9(4)	C16	N15	S2	119.8(3)
C15	C27	C8	110.5(5)	C23	N15	S2	111.8(3)
N27	C4	S3	179.8(7)	C23	N15	C16	128.4(4)
C2	C1	C10	120.8(6)	C17	C16	N15	119.6(5)
C1	C2	C3	119.8(7)	C17	C16	C21	121.1(6)
C12	C3	C2	119.2(7)	C21	C16	N15	119.3(5)
C3	C12	N1	119.8(5)	C16	C17	C18	119.1(8)
C3	C12	C11	121.6(5)	C19	C18	C17	118.9(8)
C11	C12	N1	118.6(5)	C22	C19	C18	121.7(7)
C15	C5	C6	110.0(4)	C16	C21	C22	119.6(8)
C7	C5	C15	110.2(4)	C19	C22	C21	119.6(8)
C7	C5	C6	111.8(4)	N15	C23	C24	113.6(4)
O1	C5	C15	101.8(4)	N15	C23	C25	121.9(4)
O1	C5	C6	111.8(4)	C24	C23	C25	124.5(5)
O1	C5	C7	110.9(4)	C14	C24	C23	111.1(4)
N1	C8	C27	113.4(4)	F4	C25	C23	116.1(13)
N1	C8	C9	121.5(4)	F5	C25	F4	100.7(15)
C27	C8	C9	125.1(5)	F5	C25	C23	110.2(10)
F1	C9	F2	109.2(5)	F6	C25	F4	102.8(19)
F1	C9	F3	107.3(5)	F6	C25	F5	111(2)
F1	C9	C8	112.0(4)	F6	C25	C23	114.5(14)
F2	C9	F3	106.2(5)	F5A	C25	C23	109.2(13)
F2	C9	C8	110.5(4)	F5A	C25	F6A	116(2)
F3	C9	C8	111.4(5)	F5A	C25	F4A	106.9(18)
C1	C10	C11	120.0(7)	F6A	C25	C23	110.3(11)
C12	C11	C10	118.6(7)	F6A	C25	F4A	105(2)
O12	C13	C14	101.8(4)	F4A	C25	C23	108.3(14)
O12	C13	C20	110.3(5)	N5	C28	S4	177.8(8)

Table 3. Torsion Angles for 5-(2-hydroxypropan-2-yl)-2-phenyl-3-(trifluoromethyl)isothiazol-2-ium thiocyanate **2a**.

A	B	C	D	Angle/˚	A	B	C	D	Angle/˚
S1	N1	C12	C3	-92.0(6)	C8	N1	C12	C11	-94.8(7)
S1	N1	C12	C11	85.4(6)	C10	C1	C2	C3	2.2(14)
S1	N1	C8	C27	0.0(5)	O12	C13	C14	S2	3.4(5)
S1	N1	C8	C9	-177.9(4)	O12	C13	C14	C24	179.2(5)
S1	C15	C27	C8	-0.4(5)	C13	C14	C24	C23	-174.0(5)
S1	C15	C5	C6	-117.5(4)	C14	S2	N15	C16	-177.7(4)
S1	C15	C5	C7	118.8(4)	C14	S2	N15	C23	1.0(4)
S1	C15	C5	O1	1.1(5)	N15	S2	C14	C13	174.9(4)
S2	C14	C24	C23	2.0(5)	N15	S2	C14	C24	-1.7(4)
S2	N15	C16	C17	92.6(7)	N15	C16	C17	C18	-179.4(7)
S2	N15	C16	C21	-87.0(7)	N15	C16	C21	C22	179.4(7)
S2	N15	C23	C24	0.0(5)	N15	C23	C24	C14	-1.3(6)
S2	N15	C23	C25	-178.8(4)	N15	C23	C25	F4	-45.1(18)
N1	S1	C15	C27	0.4(4)	N15	C23	C25	F5	-158.8(13)
N1	S1	C15	C5	-178.8(4)	N15	C23	C25	F6	74(3)
N1	C12	C11	C10	-177.9(7)	N15	C23	C25	F5A	-61.9(18)
N1	C8	C9	F1	61.3(7)	N15	C23	C25	F6A	169(3)
N1	C8	C9	F2	-176.8(4)	N15	C23	C25	F4A	54.2(19)
N1	C8	C9	F3	-59.0(6)	C16	N15	C23	C24	178.5(5)
C15	S1	N1	C12	179.6(4)	C16	N15	C23	C25	-0.3(8)
C15	S1	N1	C8	-0.2(3)	C16	C17	C18	C19	1.2(15)
C15	C27	C8	N1	0.3(6)	C16	C21	C22	C19	-1.2(14)
C15	C27	C8	C9	178.1(5)	C17	C16	C21	C22	-0.2(12)
C27	C15	C5	C6	63.5(7)	C17	C18	C19	C22	-2.7(16)
C27	C15	C5	C7	-60.2(7)	C18	C19	C22	C21	2.7(15)
C27	C15	C5	O1	-177.9(5)	C20	C13	C14	S2	121.0(5)
C27	C8	C9	F1	-116.4(6)	C20	C13	C14	C24	-63.2(7)
C27	C8	C9	F2	5.5(7)	C21	C16	C17	C18	0.2(13)
C27	C8	C9	F3	123.3(6)	C23	N15	C16	C17	-85.9(8)
C1	C2	C3	C12	-1.5(14)	C23	N15	C16	C21	94.5(7)
C1	C10	C11	C12	1.1(13)	C24	C23	C25	F4	136.2(18)
C2	C1	C10	C11	-2.0(14)	C24	C23	C25	F5	22.5(14)
C2	C3	C12	N1	178.0(7)	C24	C23	C25	F6	-104(3)
C2	C3	C12	C11	0.7(12)	C24	C23	C25	F5A	119.4(17)
C3	C12	C11	C10	-0.5(11)	C24	C23	C25	F6A	-10(3)
C12	N1	C8	C27	-179.8(5)	C24	C23	C25	F4A	-124.4(18)
C12	N1	C8	C9	2.3(7)	C25	C23	C24	C14	177.5(5)
C5	C15	C27	C8	178.6(5)	C26	C13	C14	S2	-114.8(5)
C8	N1	C12	C3	87.8(7)	C26	C13	C14	C24	61.0(7)

8. NMR (^{1}H, ^{13}C, ^{19}F) spectra of the isothiazolium thiocyanates 2

5-(2-Hydroxypropan-2-yl)-2-phenyl-3-(trifluoromethyl)isothiazol-2-ium thiocyanate (2a)

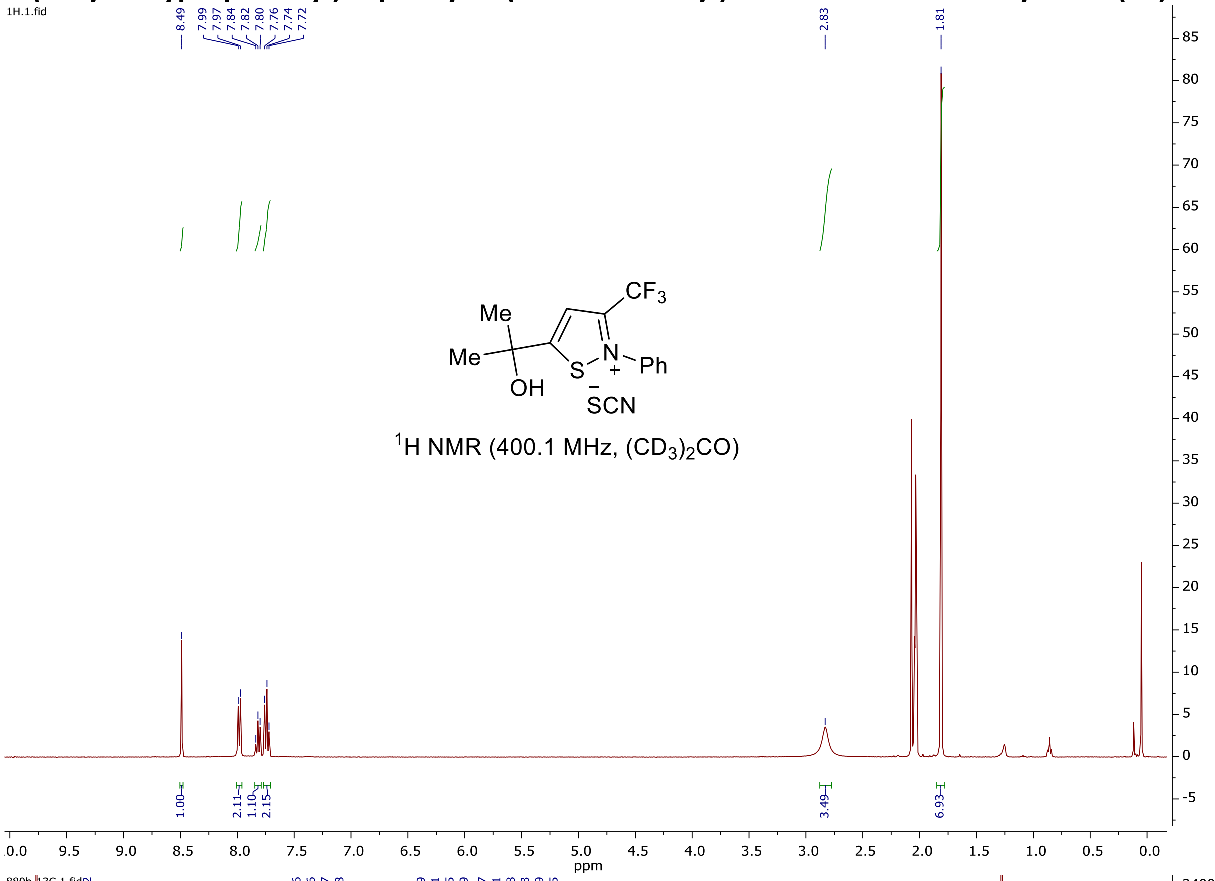

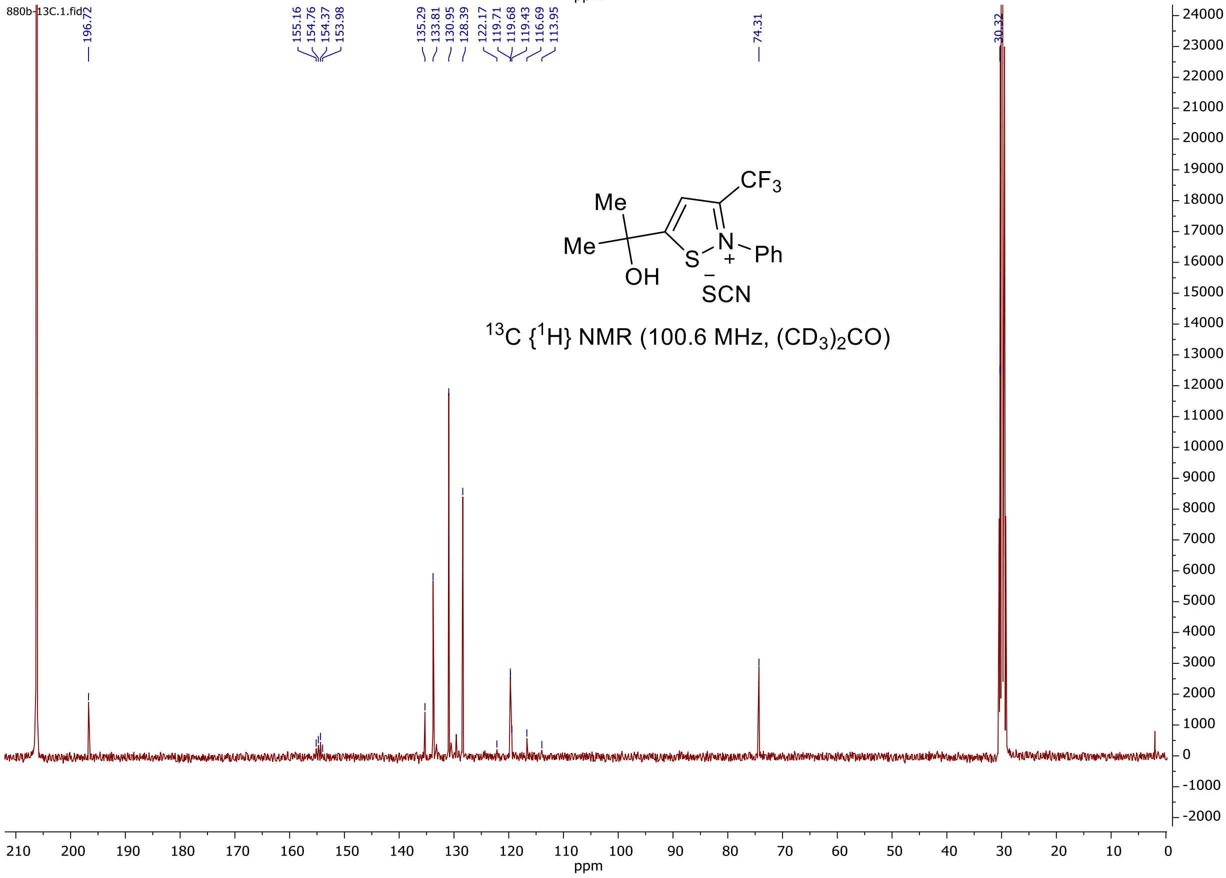

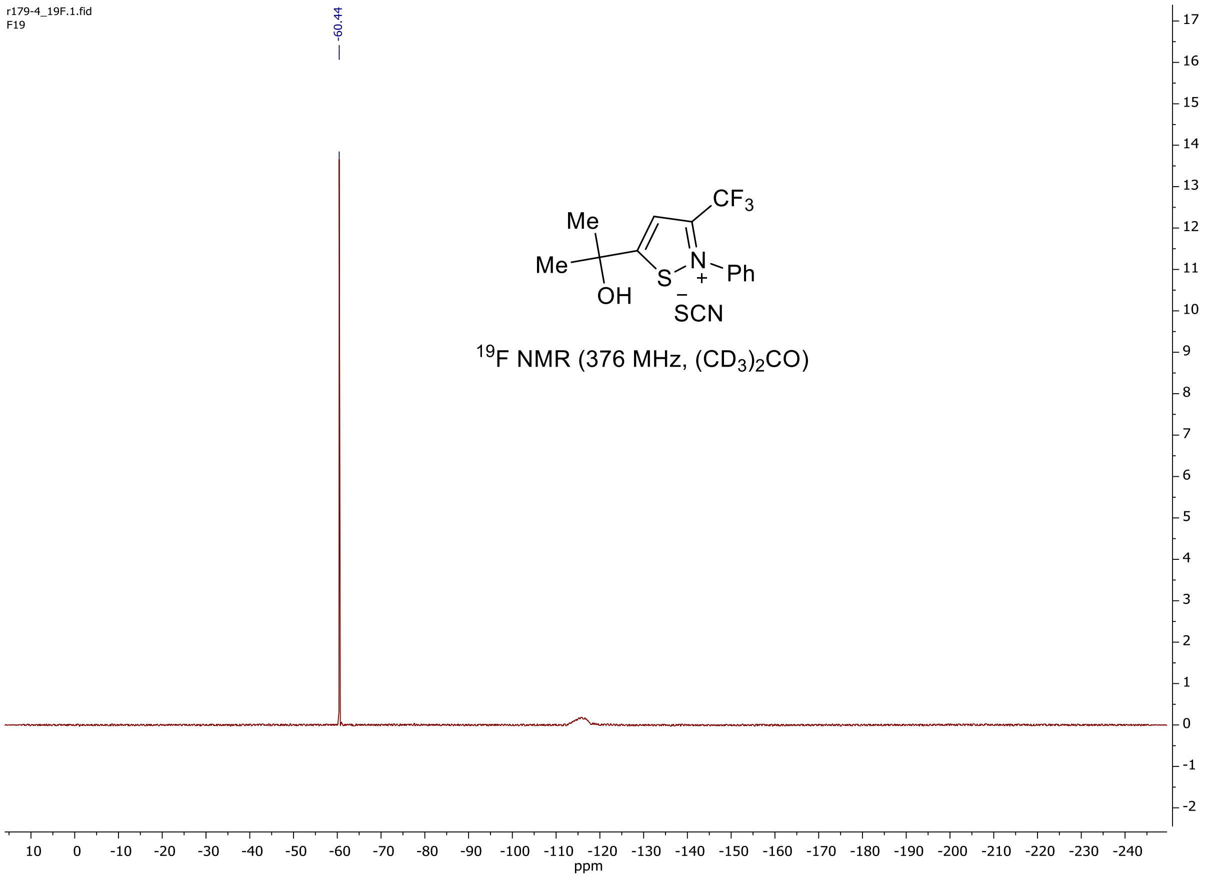

5-(2-Hydroxybutan-2-yl)-2-phenyl-3-(trifluoromethyl)isothiazol-2-ium thiocyanate (2b)

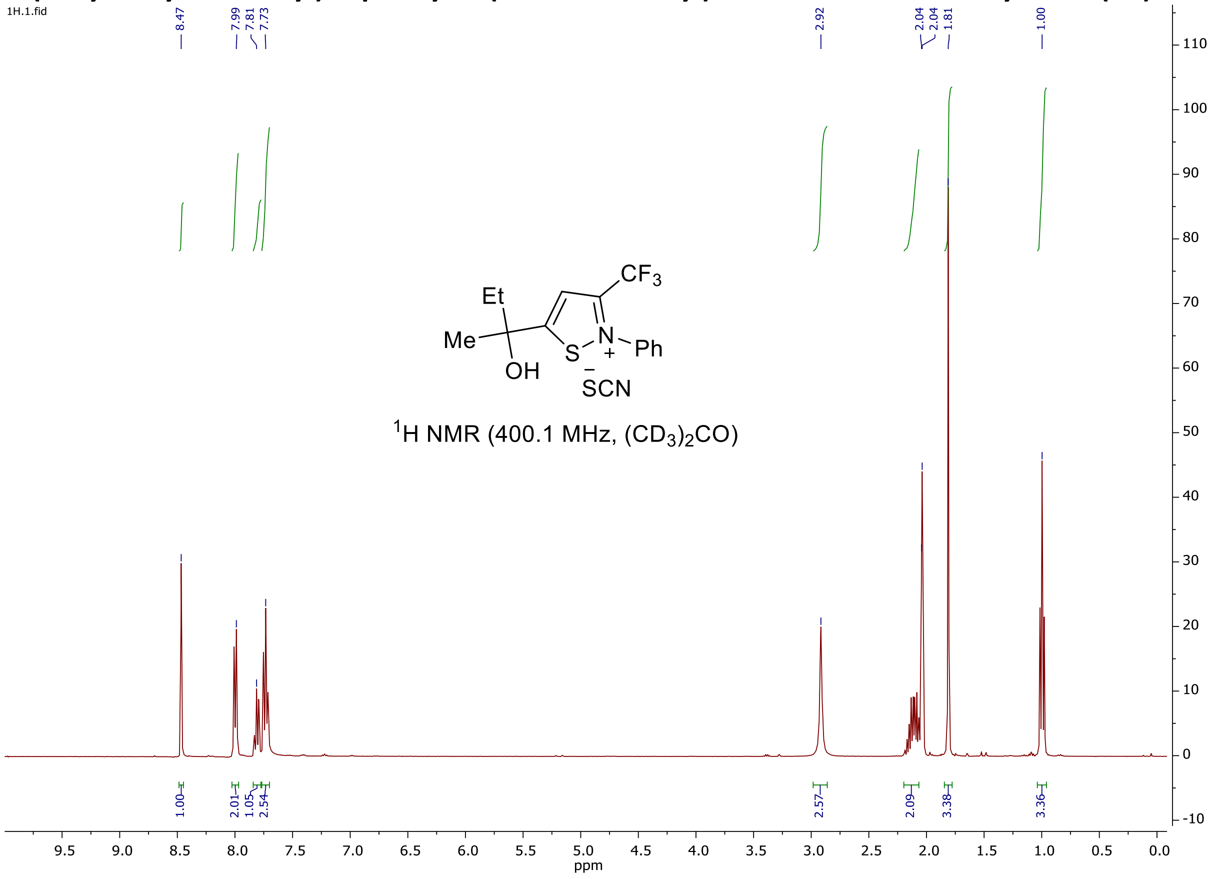

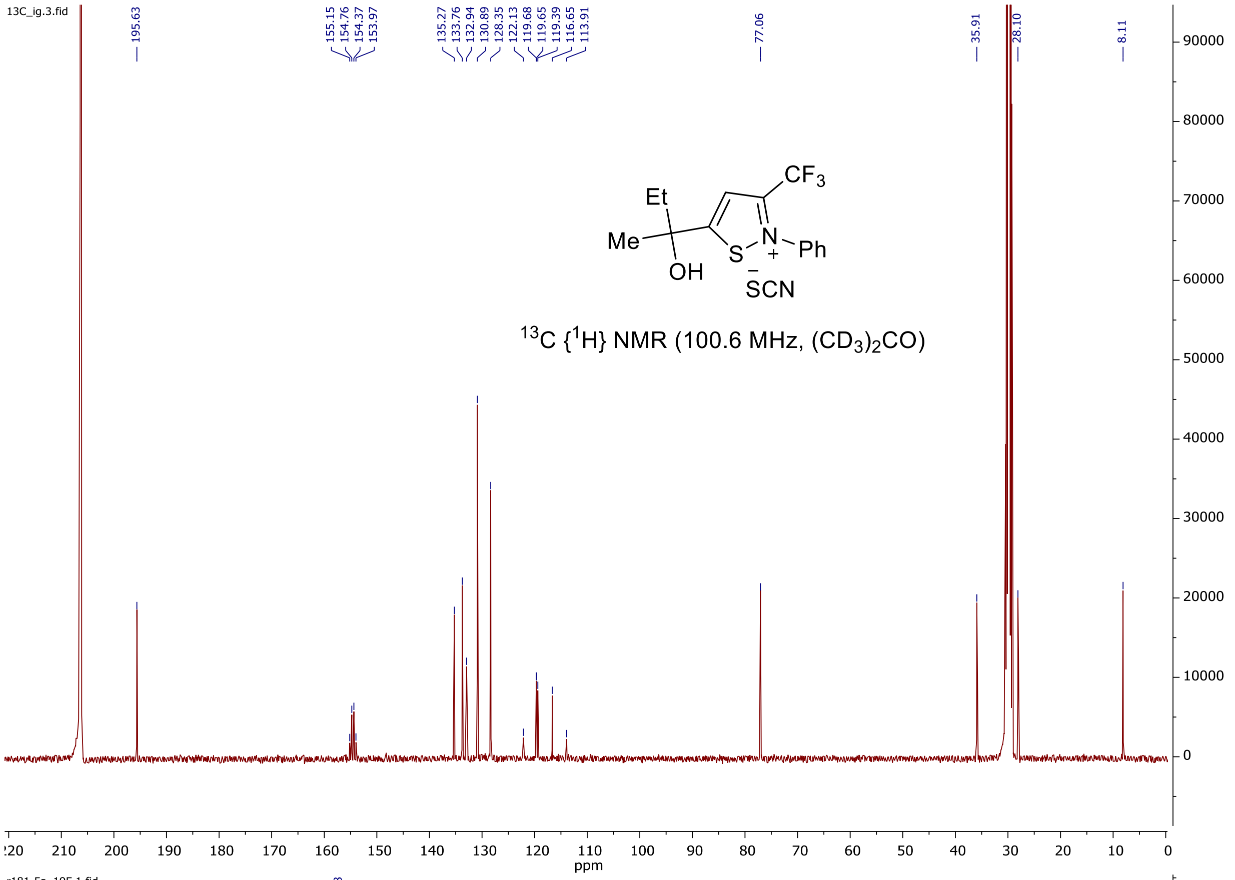
13C_ig.3.fid
195.63
155.15
154.76
154.37
153.97
135.27
133.76
132.94
130.89
128.35
122.13
119.68
119.65
119.39
116.65
113.91
77.06
35.91
28.10
8.11
CF3
Et
Me
N
Ph
S
OH
SCN
13C {1H} NMR (100.6 MHz, (CD3)2CO)
ppm

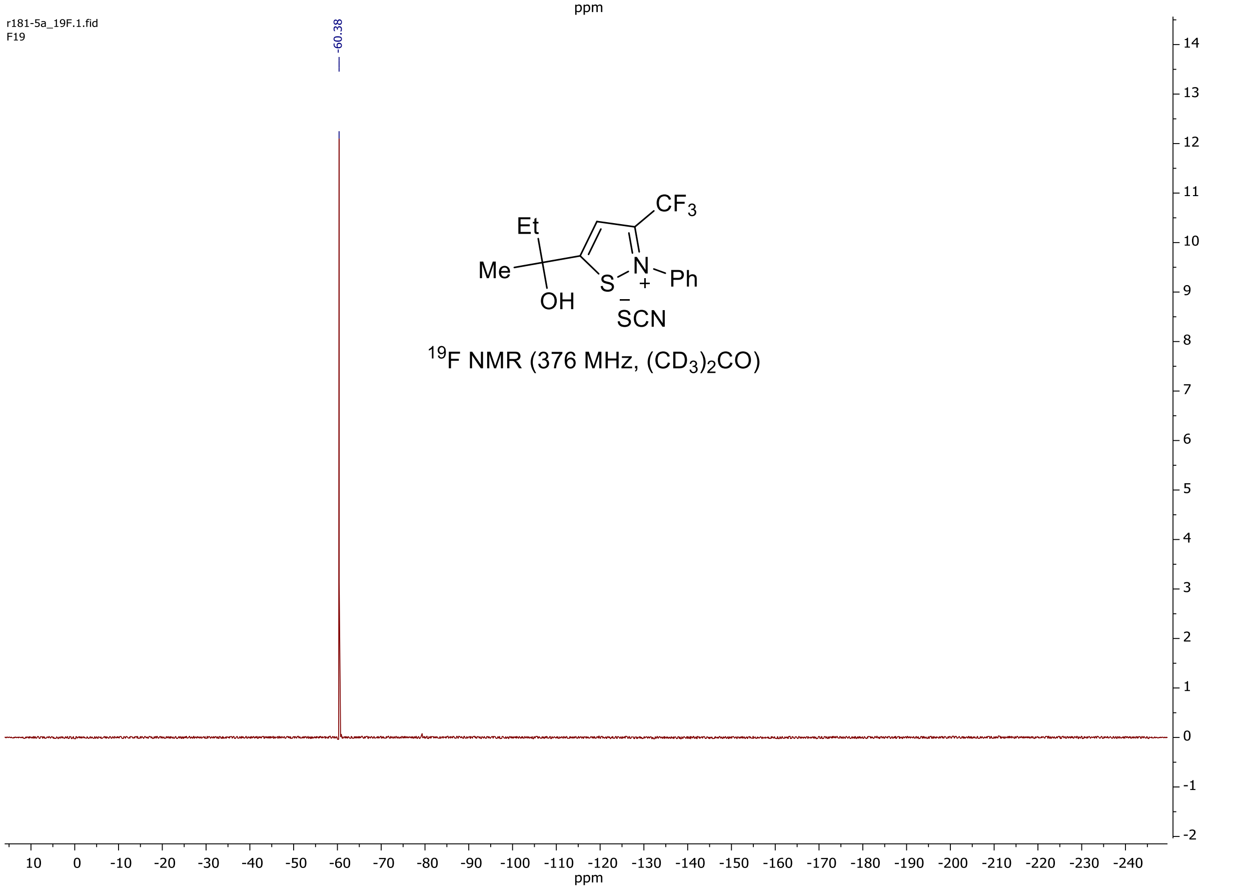
r181-5a_19F.1.fid
F19
-60.38
CF3
Et
Me
N
Ph
S
OH
SCN
19F NMR (376 MHz, (CD3)2CO)
ppm

5-(1-Hydroxycyclohexyl)-2-phenyl-3-(trifluoromethyl)isothiazol-2-ium thiocyanate (2c)

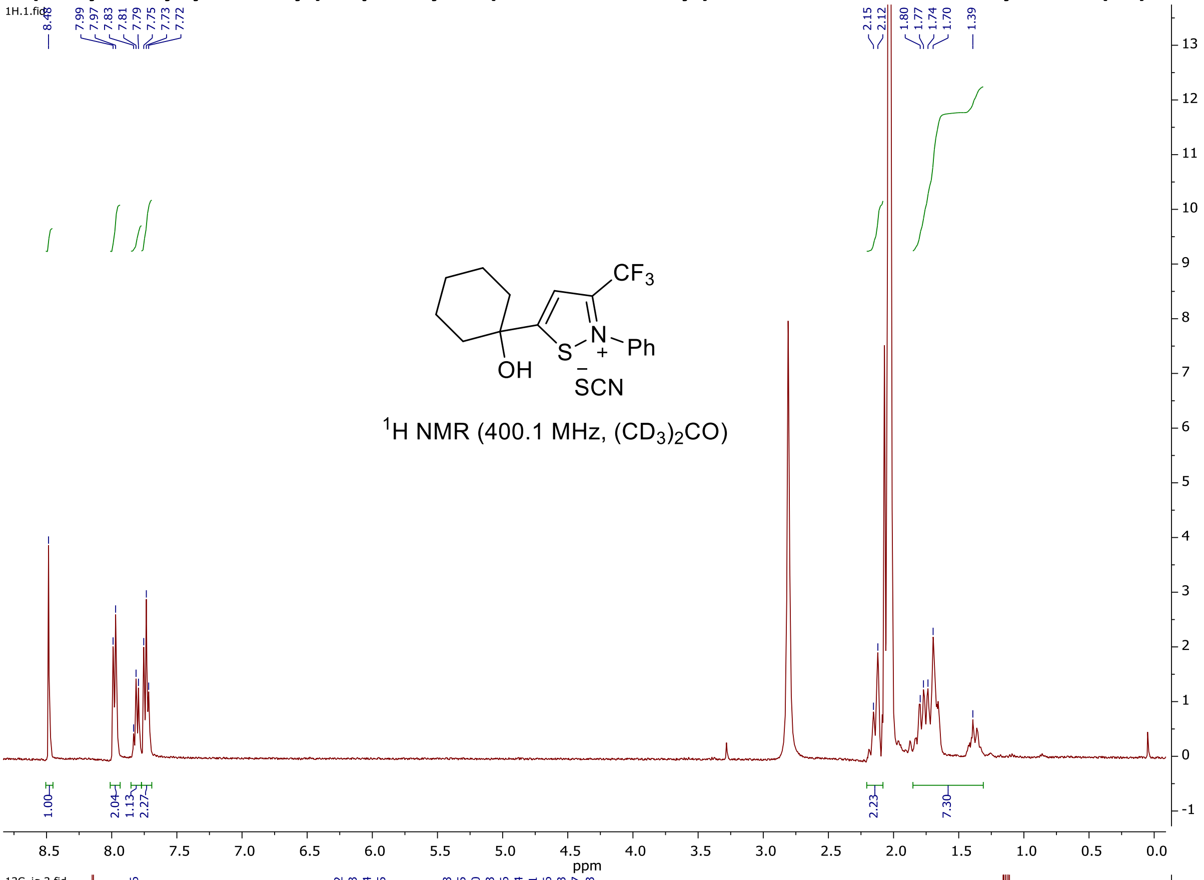

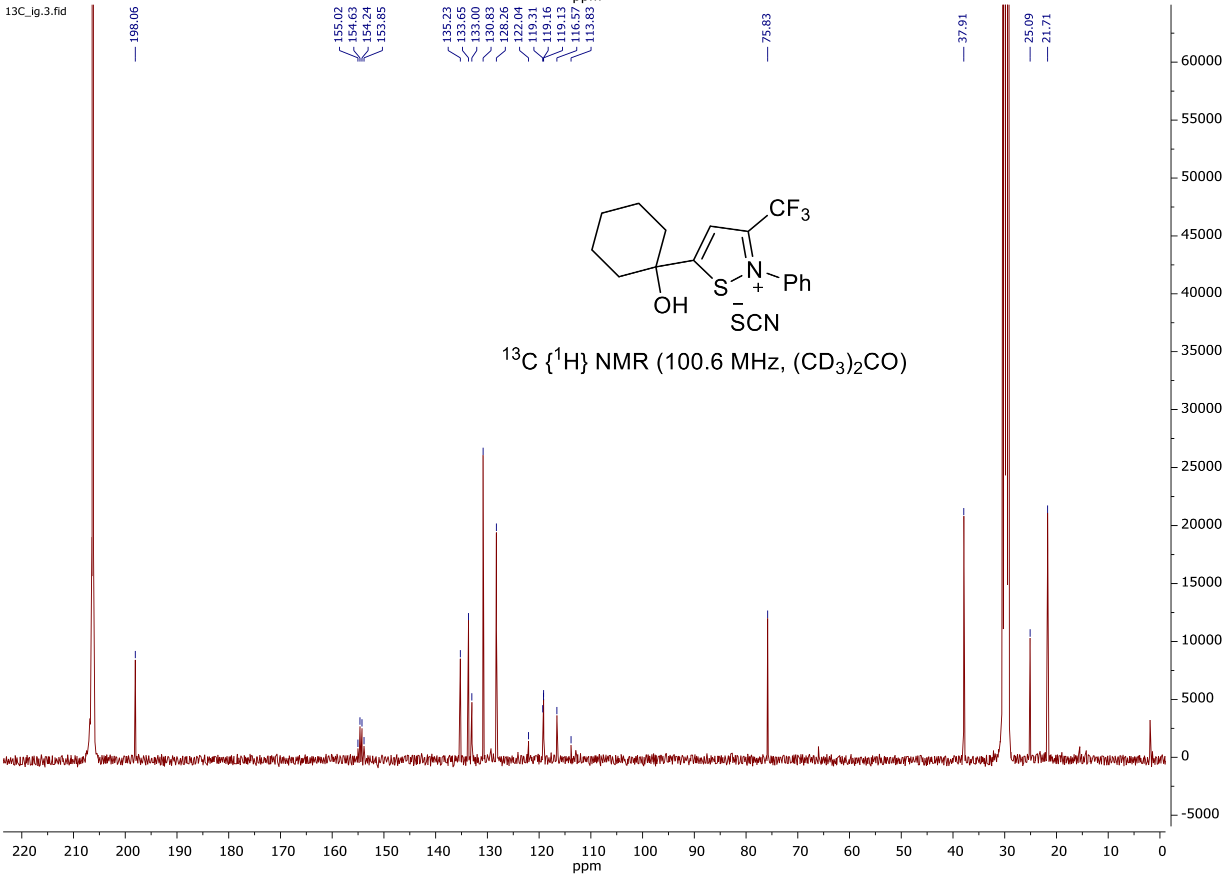

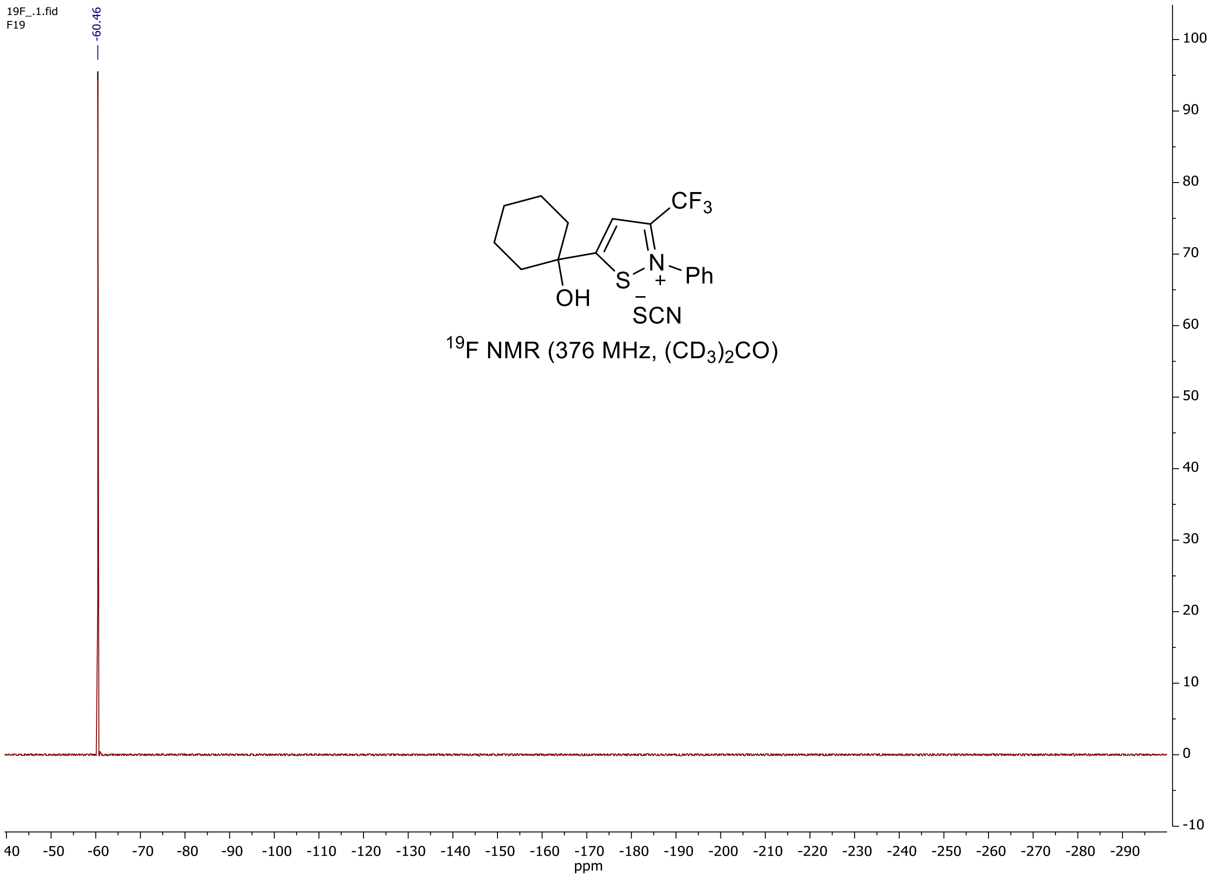

5-(1-Hydroxy-1-phenylethyl)-2-phenyl-3-(trifluoromethyl)isothiazol-2-ium thiocyanate (2d)

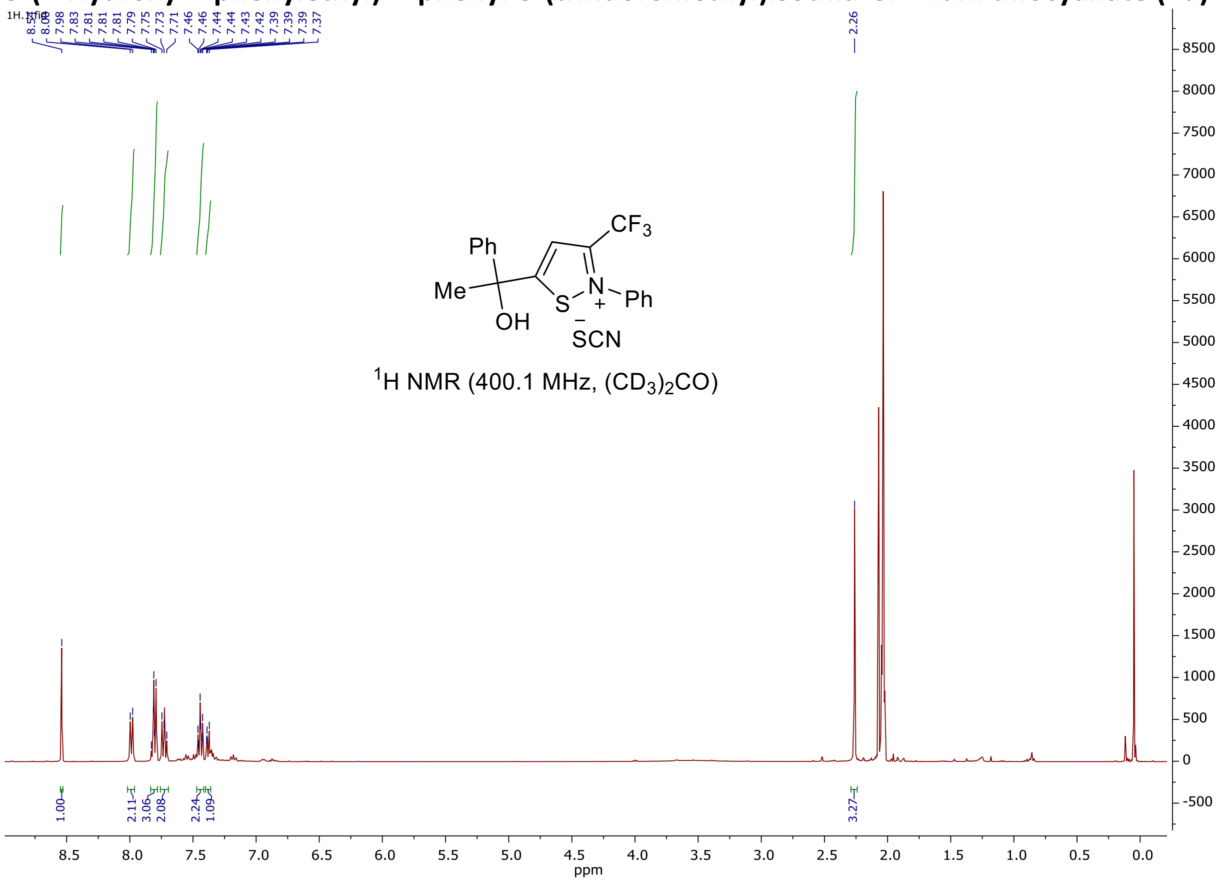

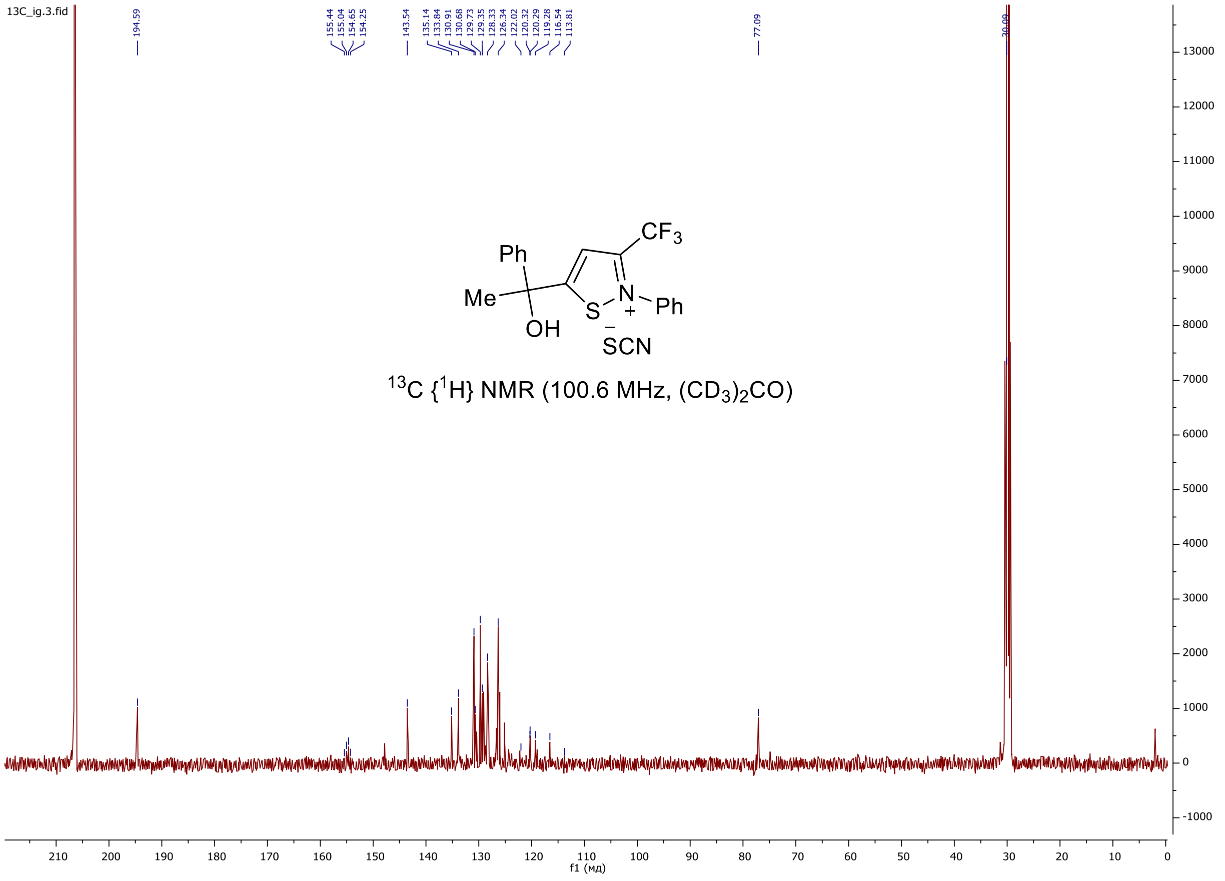

13C_ig.3.fid
194.59
155.44
155.04
154.65
154.25
143.54
135.14
133.84
130.91
130.68
129.73
129.35
128.33
126.34
122.02
120.32
120.29
119.28
116.54
113.81
77.09
30.09
Ph
Me
OH
CF3
N
+
Ph
S
−
SCN
13C {1H} NMR (100.6 MHz, (CD3)2CO)
f1 (мд)

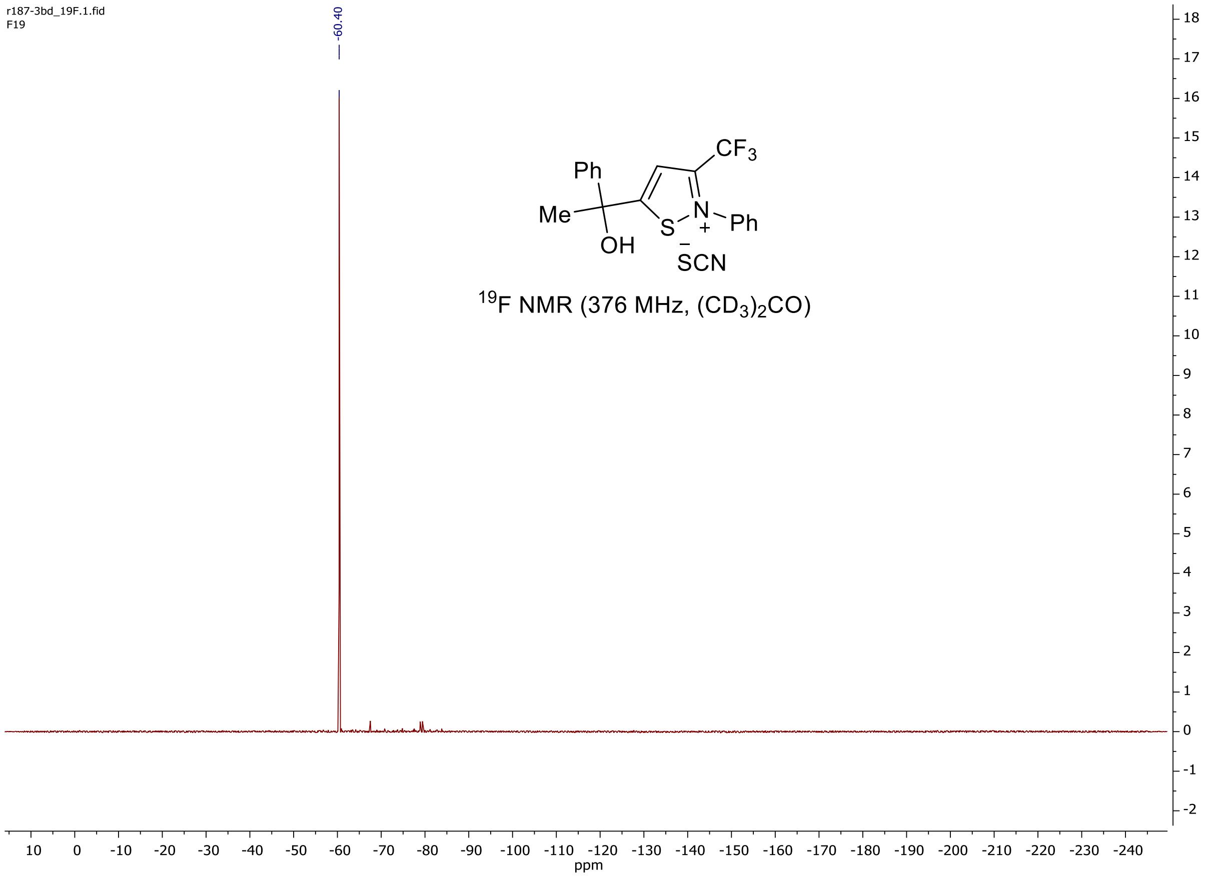

r187-3bd_19F.1.fid
F19
-60.40
Ph
Me
OH
CF3
N
+
Ph
S
−
SCN
19F NMR (376 MHz, (CD3)2CO)
ppm

5-(Hydroxydiphenylmethyl)-2-phenyl-3-(trifluoromethyl)isothiazol-2-ium thiocyanate (2e)

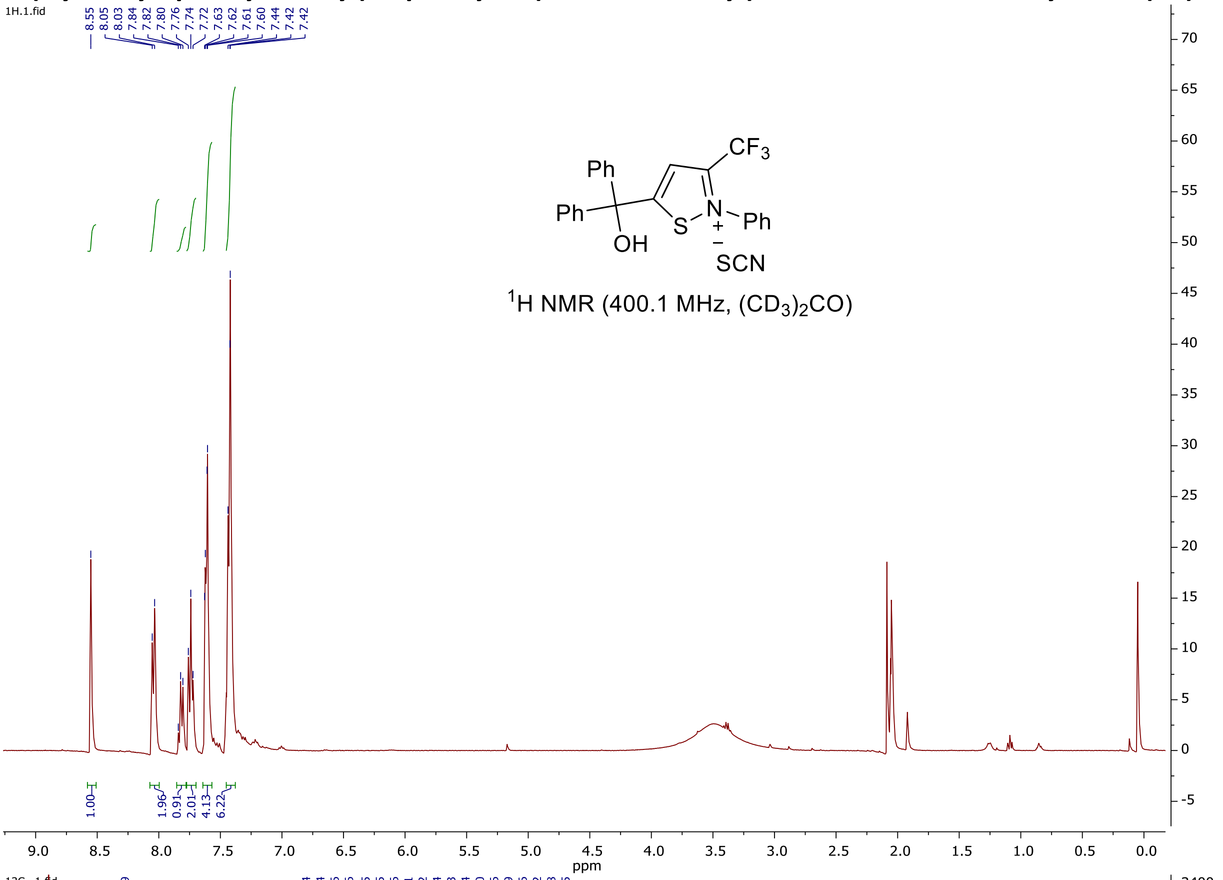

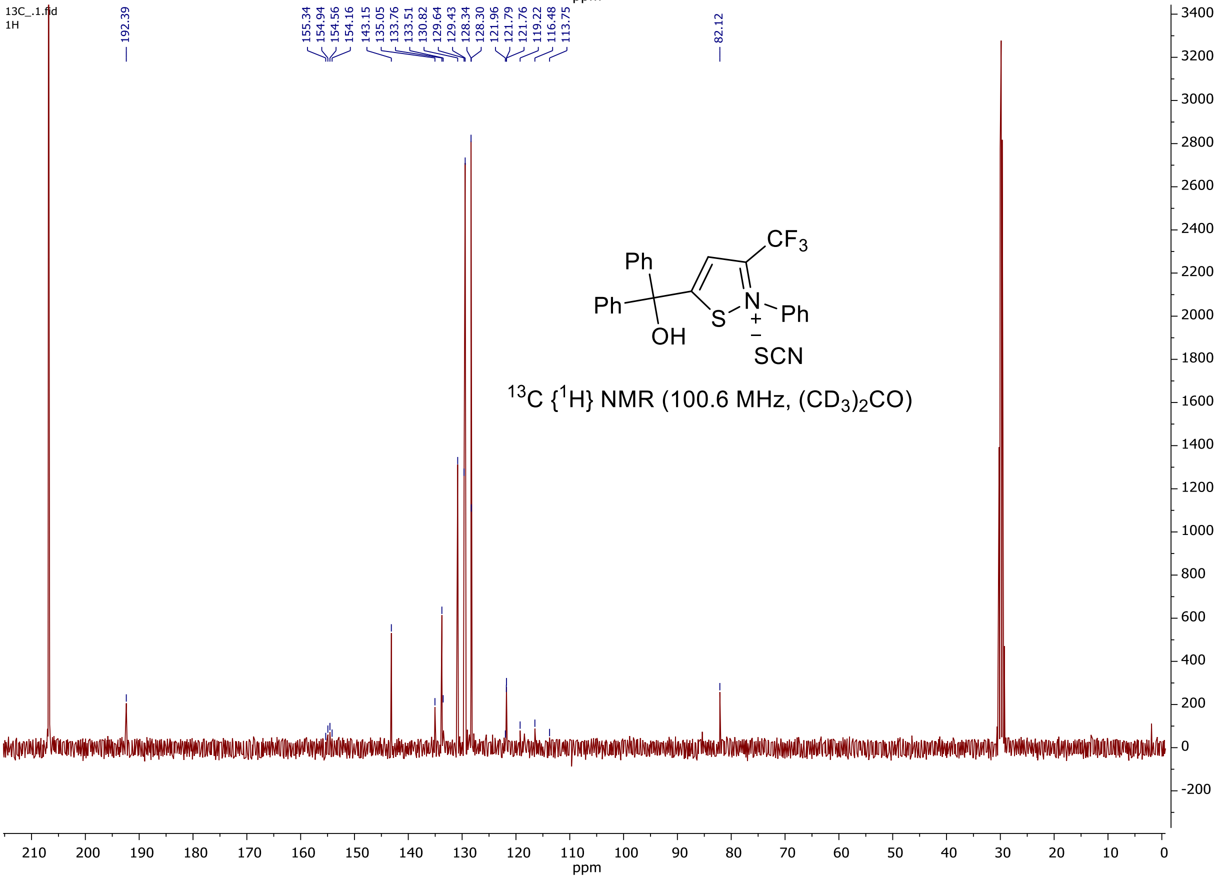

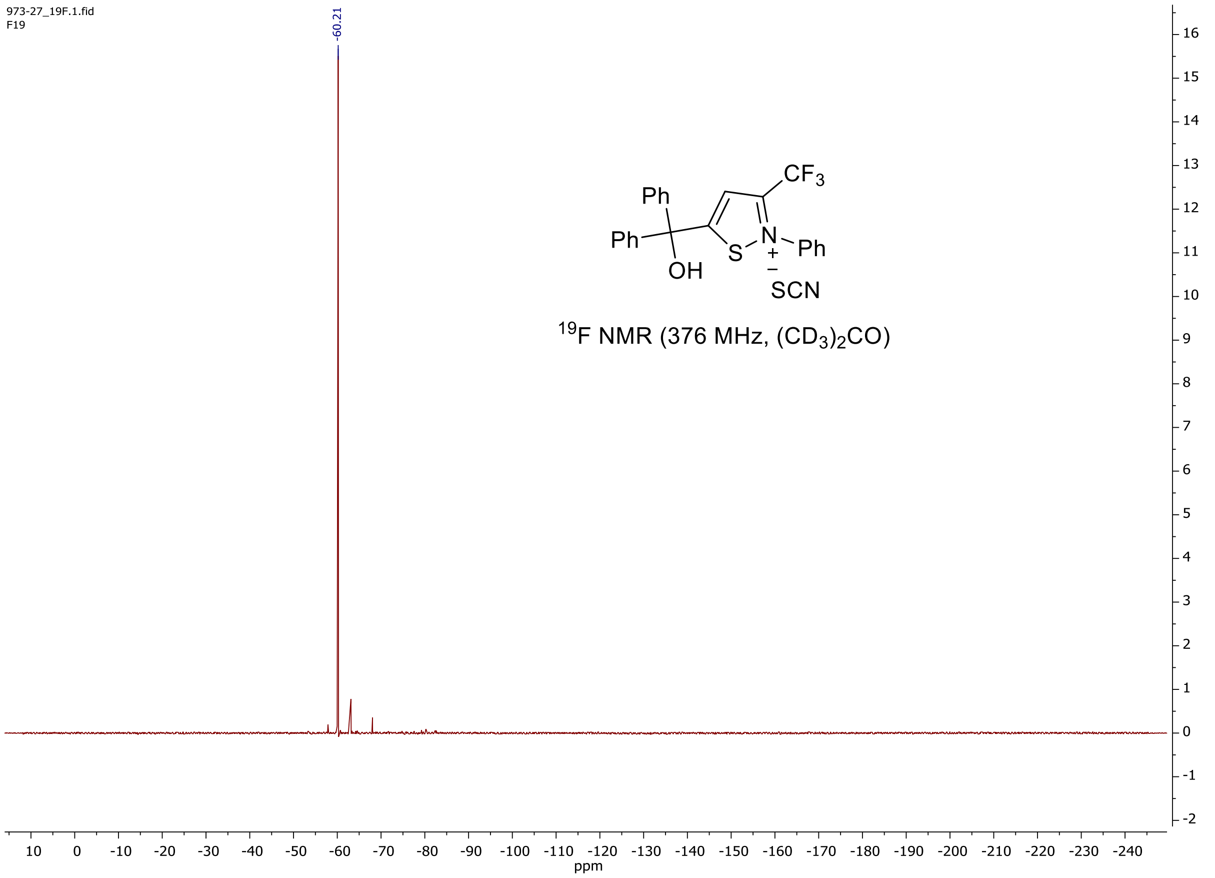

2-Butyl-5-(2-hydroxypropan-2-yl)-3-(trifluoromethyl)isothiazol-2-ium thiocyanate (2h)

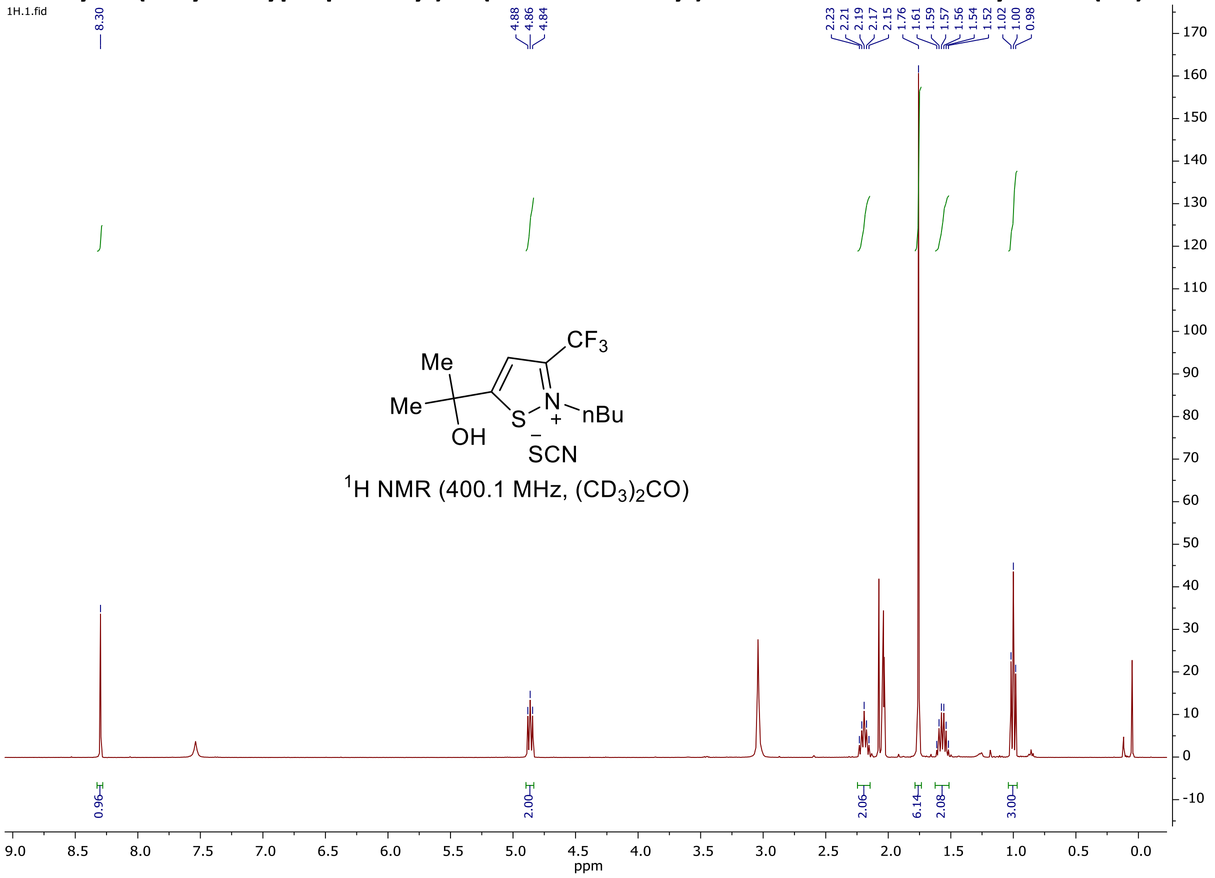

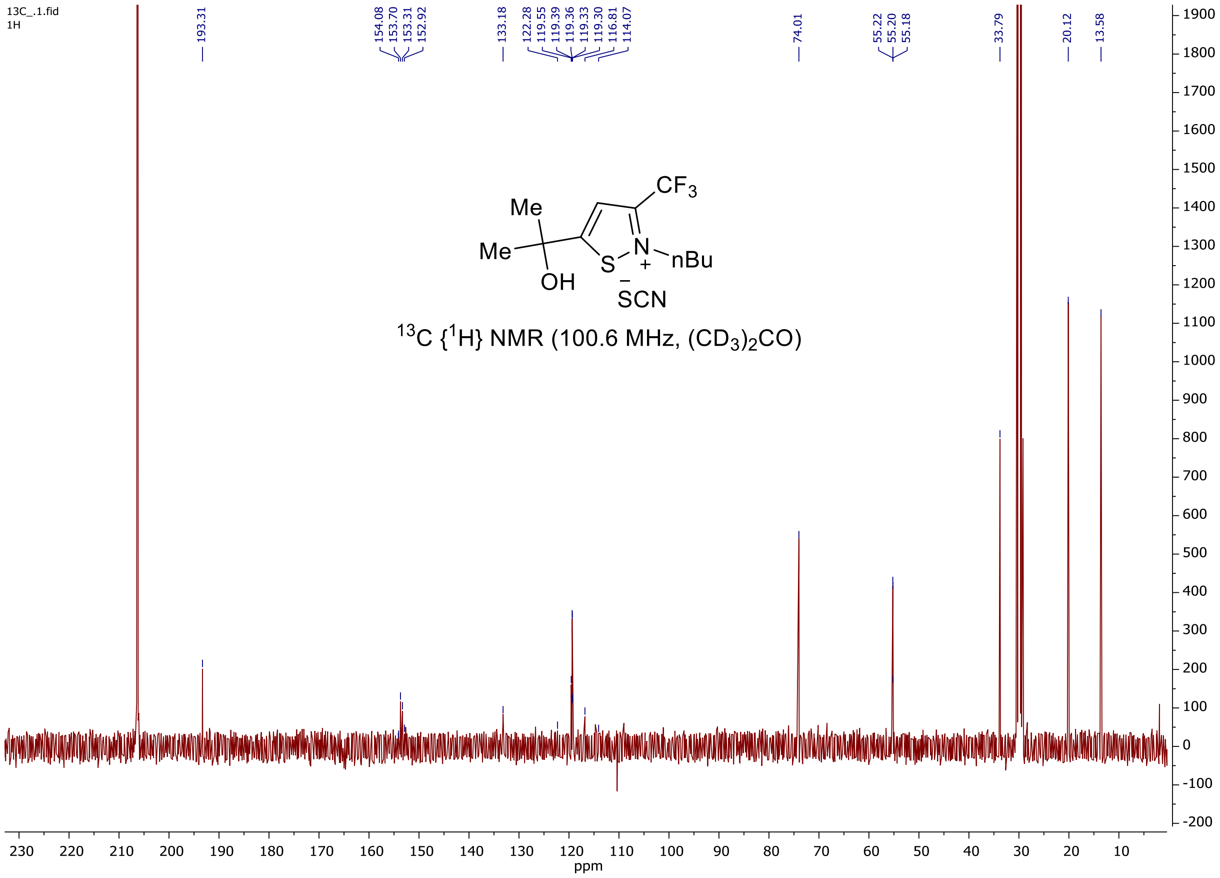
13C_.1.fid
1H
193.31
154.08
153.70
153.31
152.92
133.18
122.28
119.55
119.39
119.36
119.33
119.30
116.81
114.07
74.01
55.22
55.20
55.18
33.79
20.12
13.58
Me
Me
OH
CF3
N
+
nBu
S
−
SCN
13C {1H} NMR (100.6 MHz, (CD3)2CO)
ppm

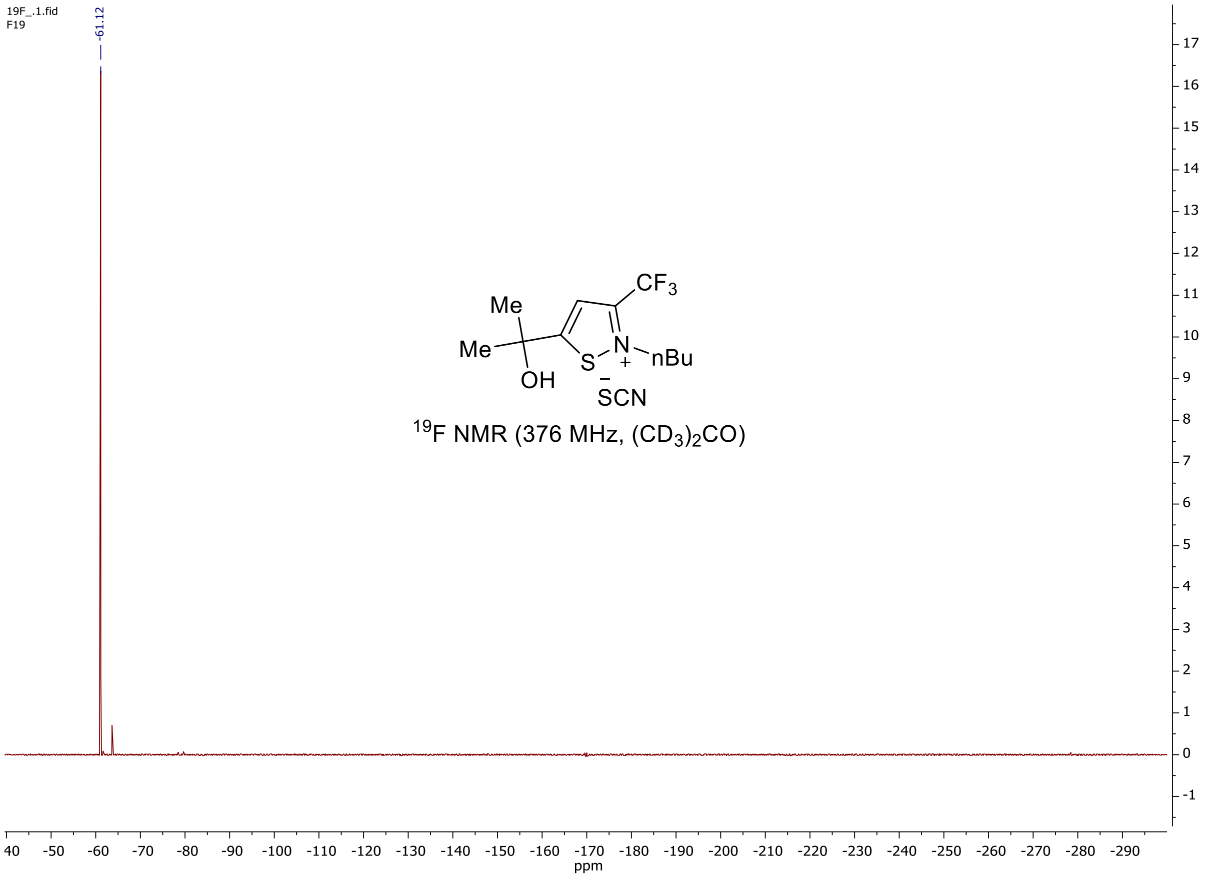
19F_.1.fid
F19
-61.12
Me
Me
OH
CF3
N
+
nBu
S
−
SCN
19F NMR (376 MHz, (CD3)2CO)
ppm

5-(2-Hydroxypropan-2-yl)-3-(perfluoropropyl)-2-phenylisothiazol-2-ium thiocyanate (2i)

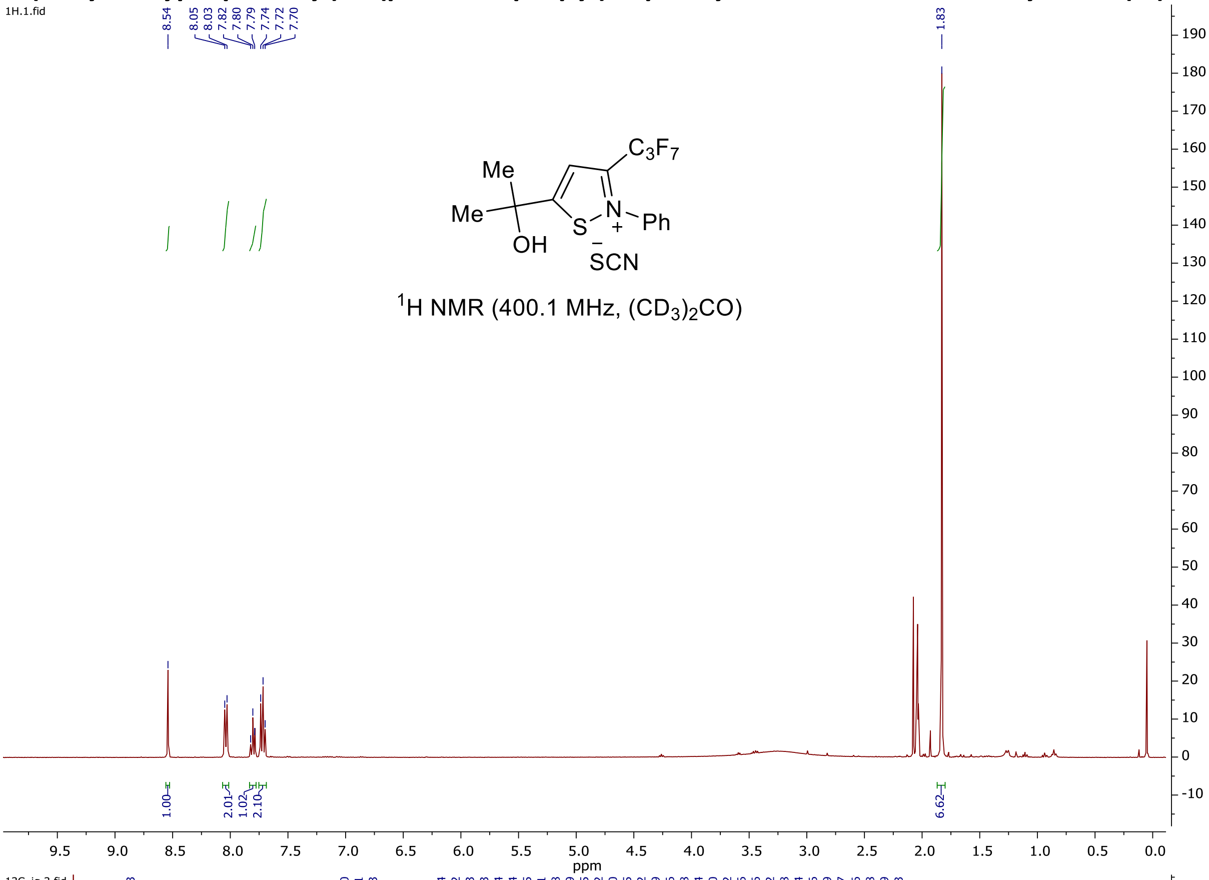

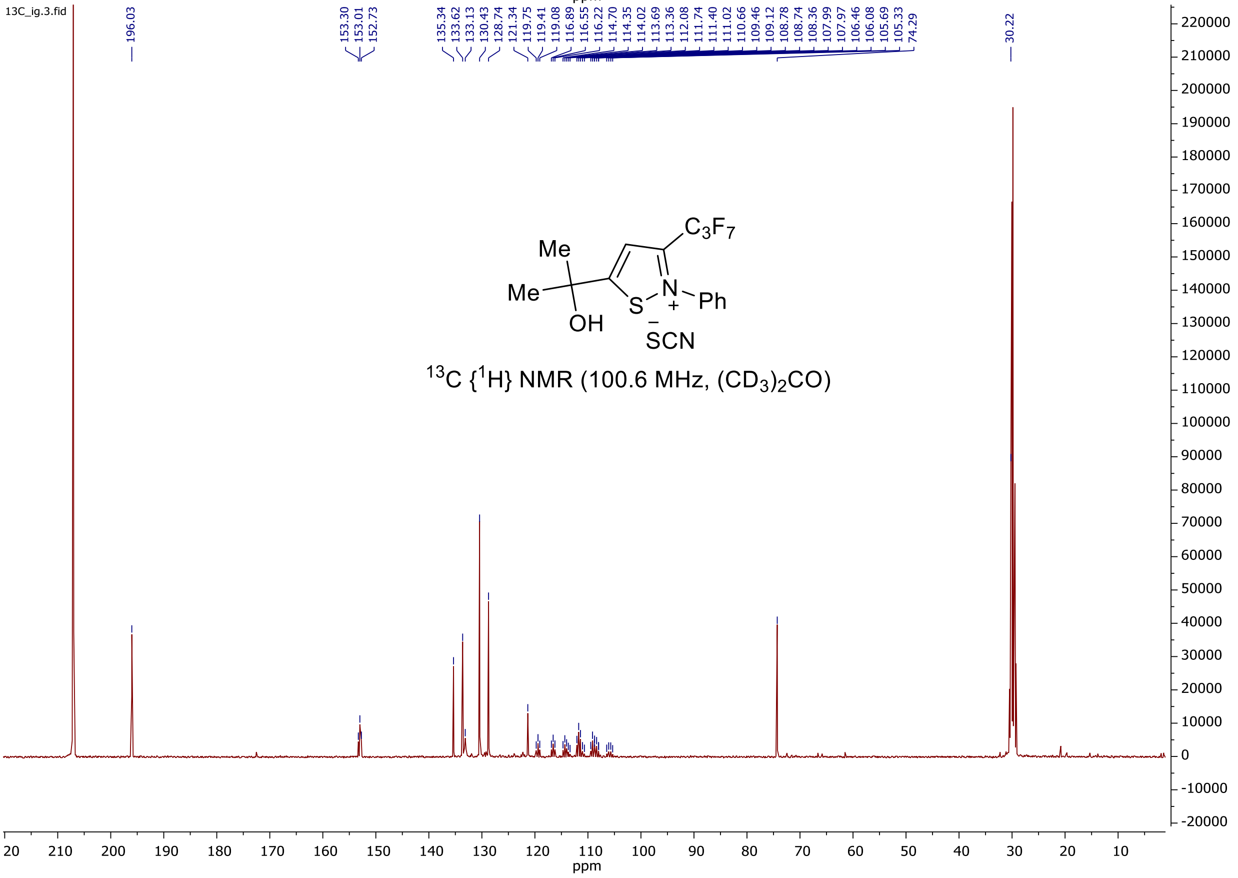

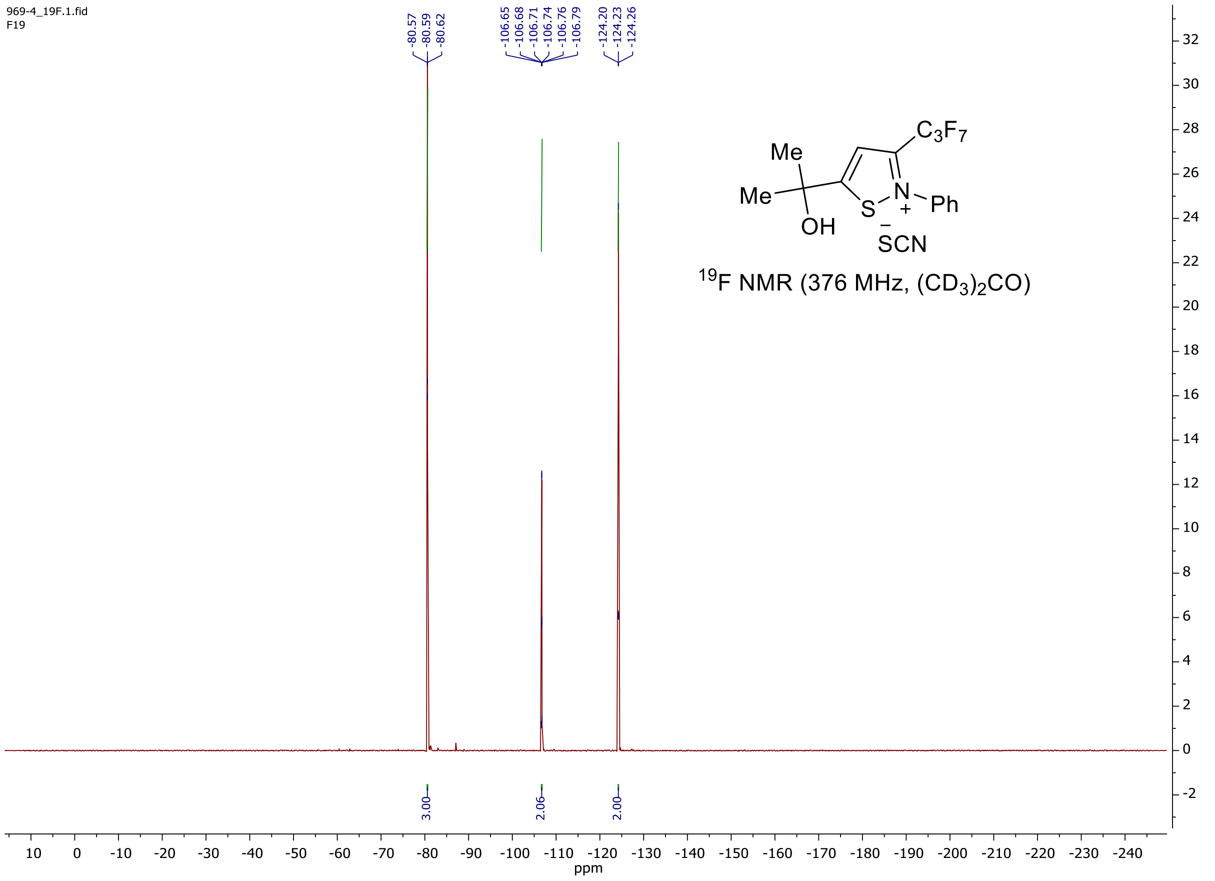

9. NMR spectra (^{1}H, ^{13}C, ^{19}F) of 4-thiocyanato-2,5-dihydrofuran-2-amines 3

5,5-Dimethyl-*N*-phenyl-4-thiocyanato-2-(trifluoromethyl)-2,5-dihydrofuran-2-amine (3a)

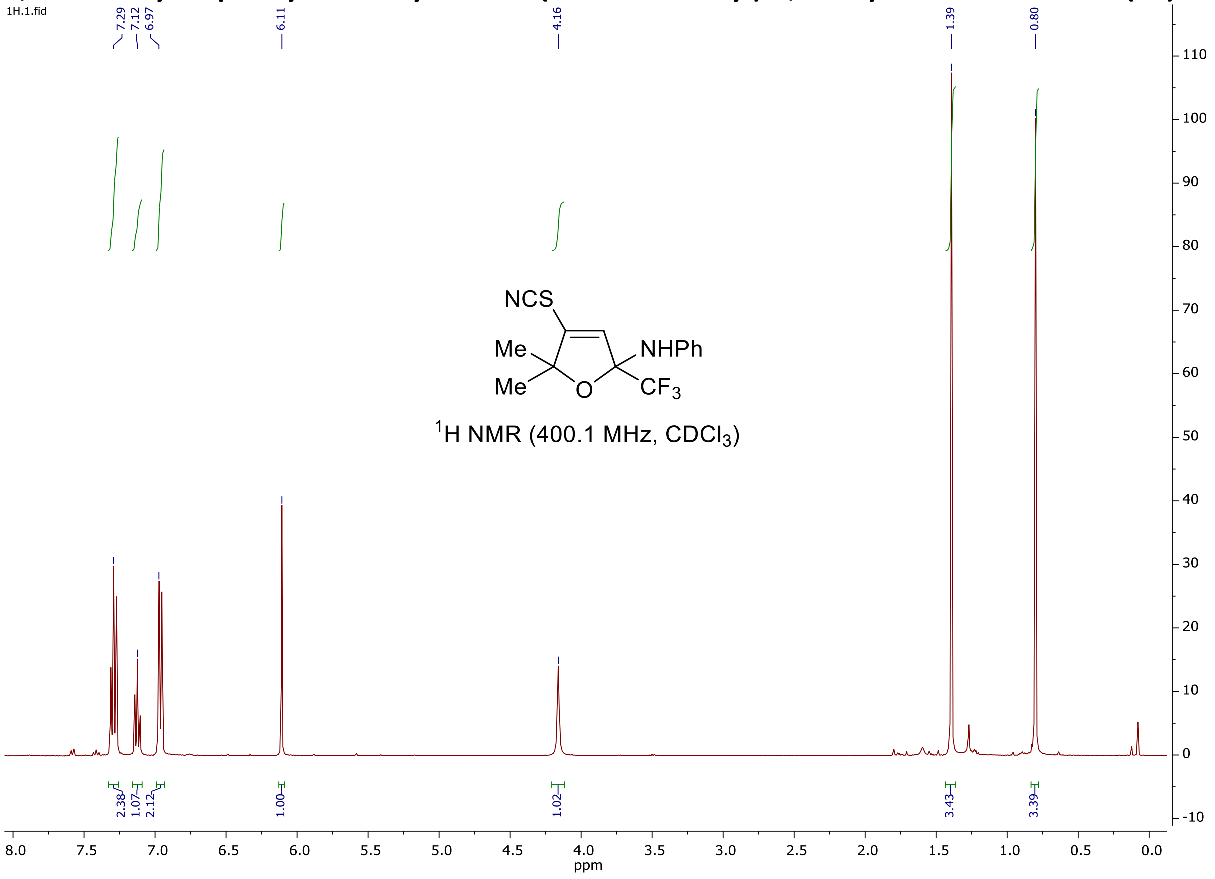

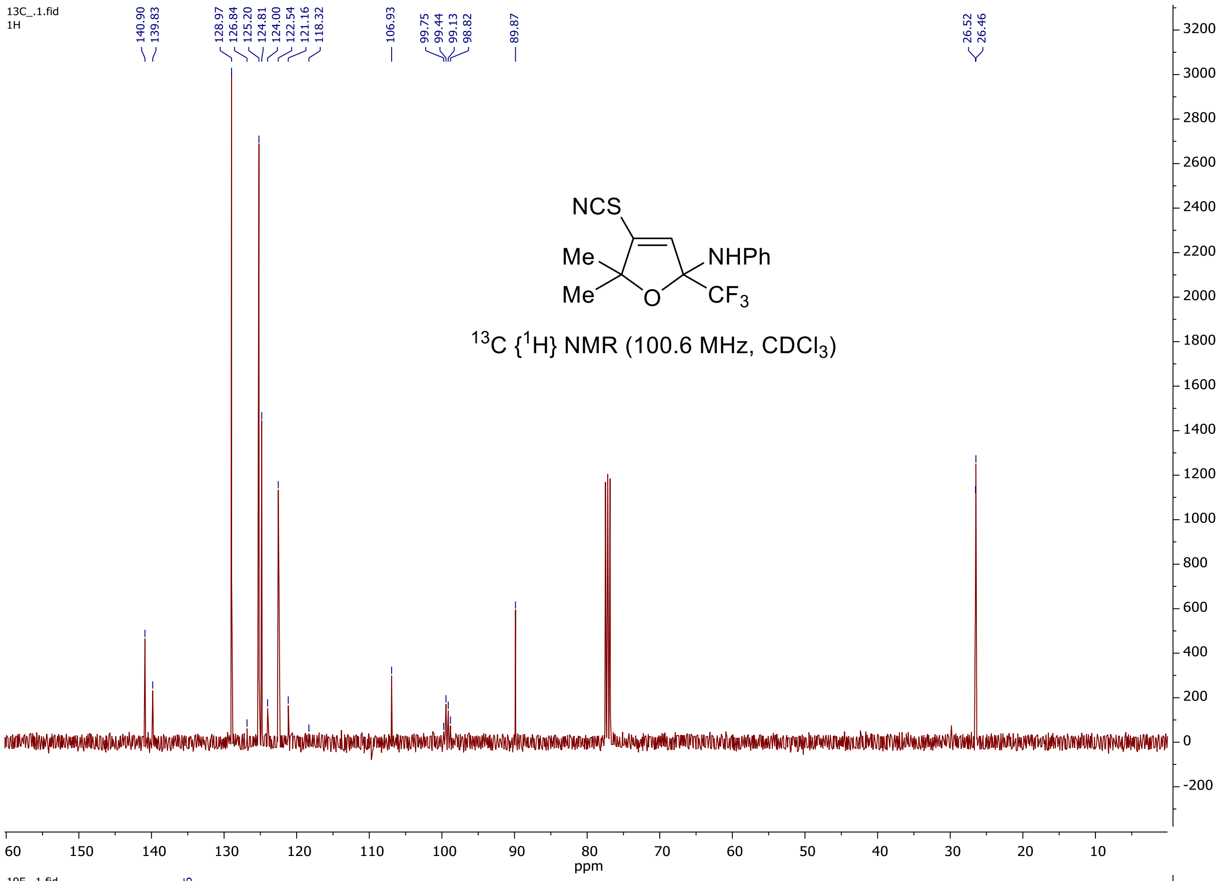
13C_.1.fid
1H
140.90
139.83
128.97
126.84
125.20
124.81
124.00
122.54
121.16
118.32
106.93
99.75
99.44
99.13
98.82
89.87
26.52
26.46
NCS
Me
Me
O
NHPh
CF3
13C {1H} NMR (100.6 MHz, CDCl3)
ppm

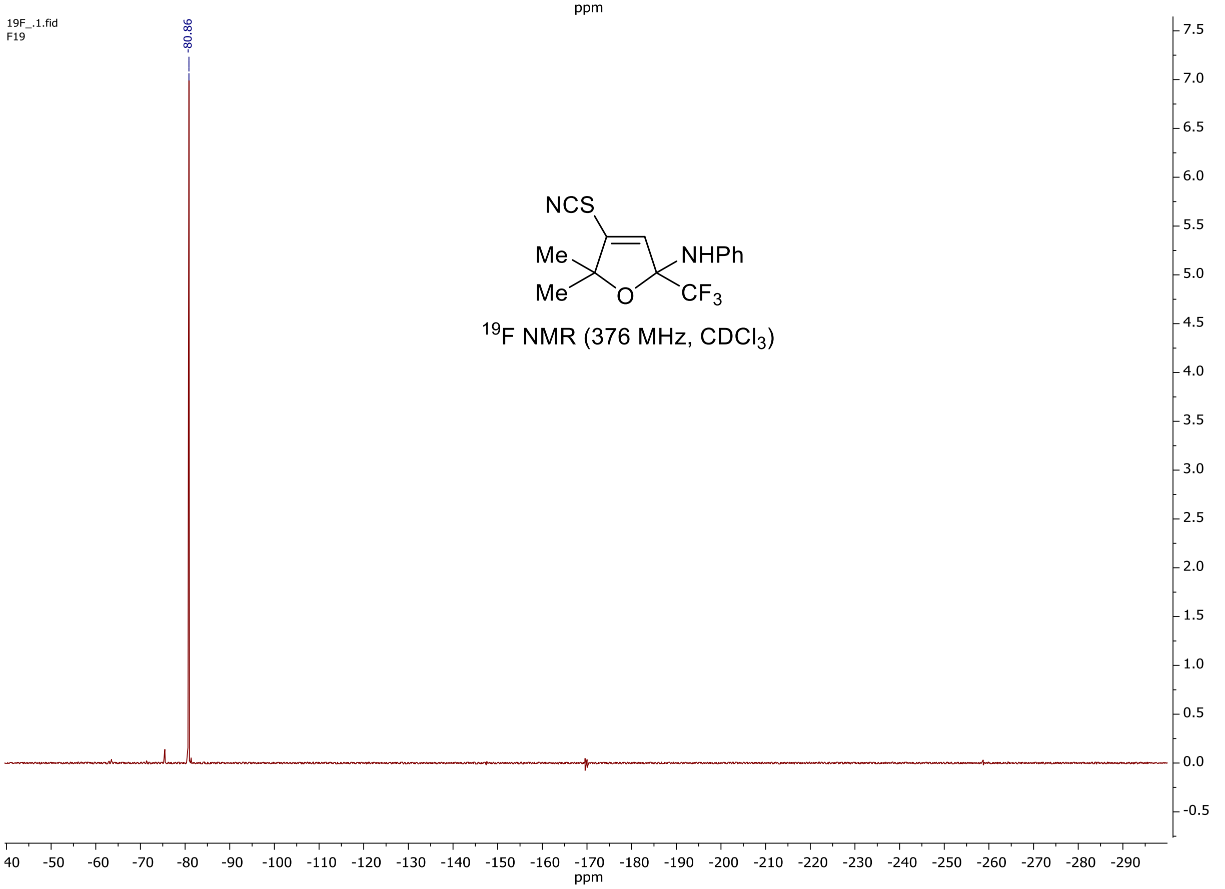
19F_.1.fid
F19
-80.86
NCS
Me
Me
O
NHPh
CF3
19F NMR (376 MHz, CDCl3)
ppm

5-Ethyl-5-methyl-*N*-phenyl-4-thiocyanato-2-(trifluoromethyl)-2,5-dihydrofuran-2-amine (3b)

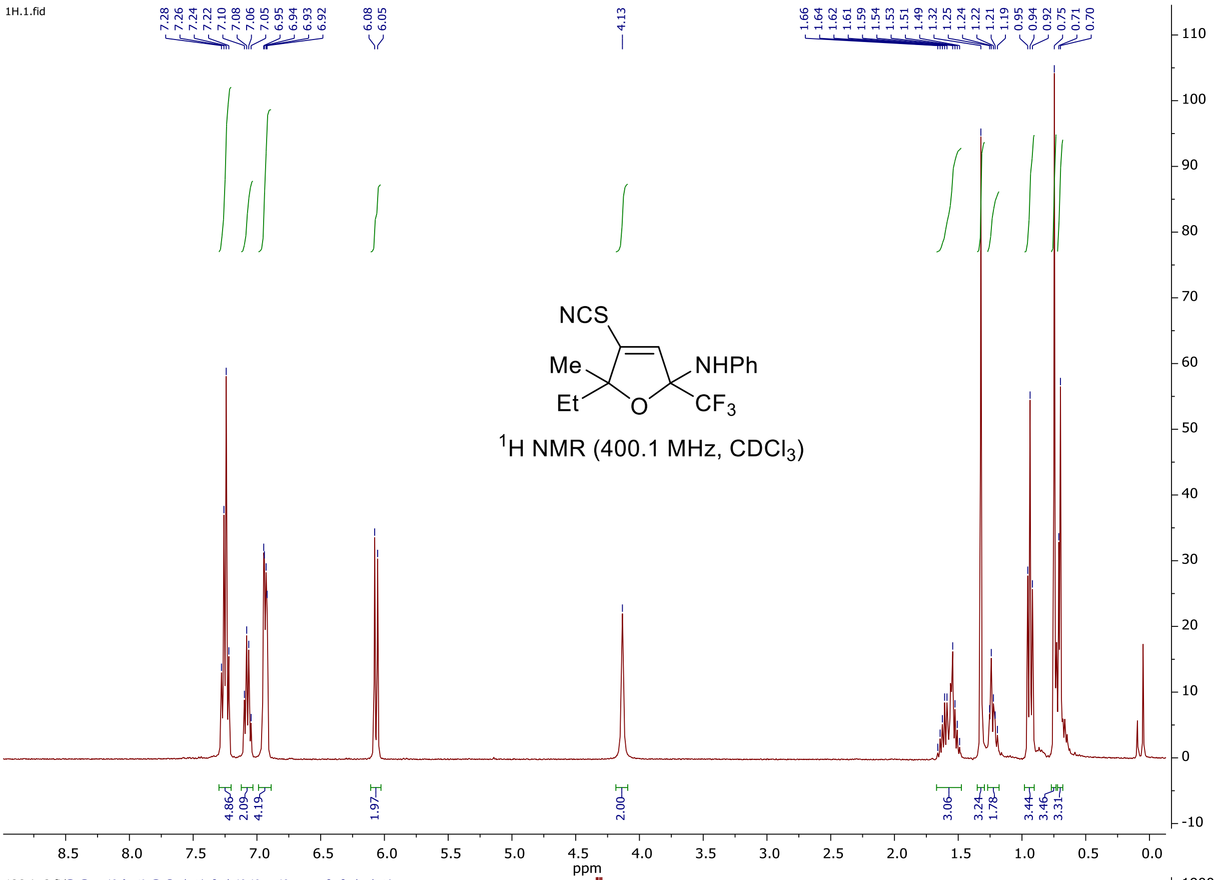

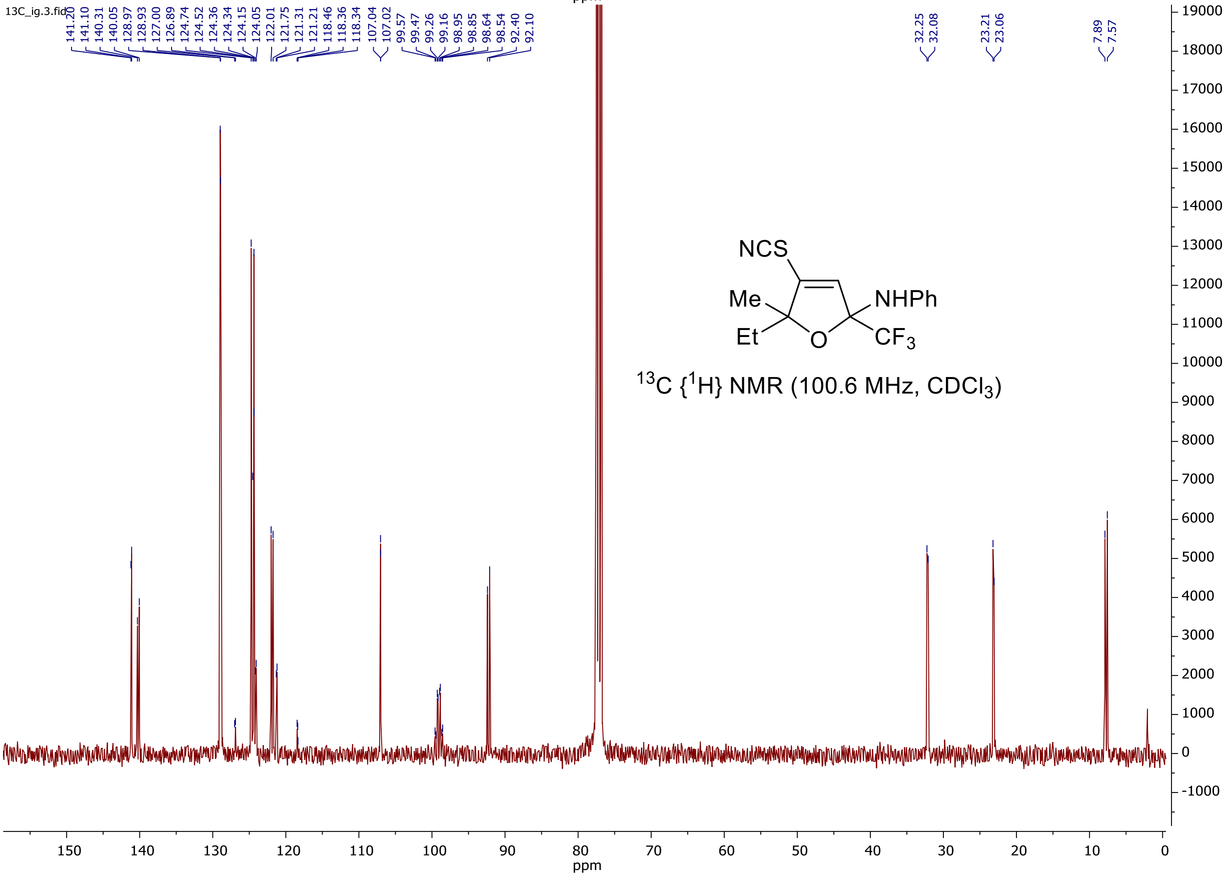

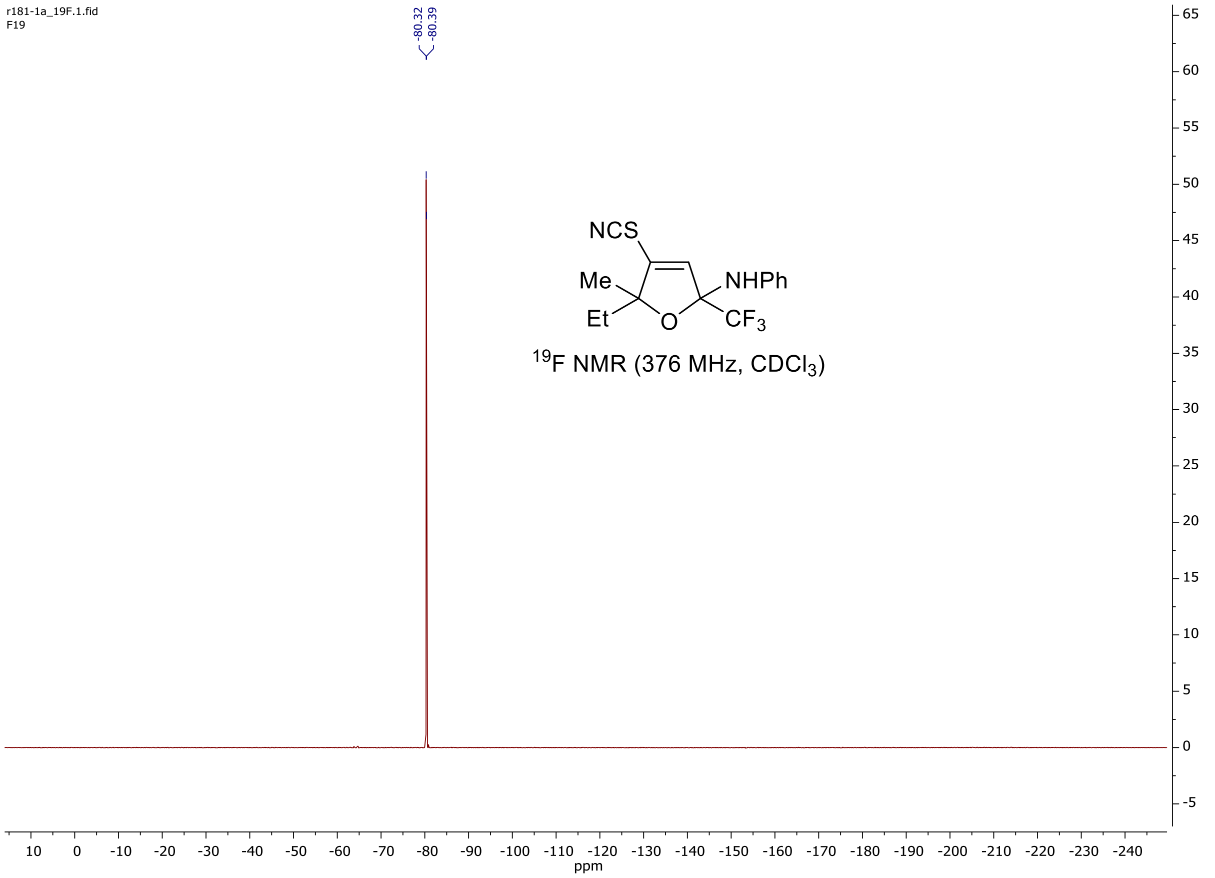

N-Phenyl-4-thiocyanato-2-(trifluoromethyl)-1-oxaspiro[4.5]dec-3-en-2-amine (3c)

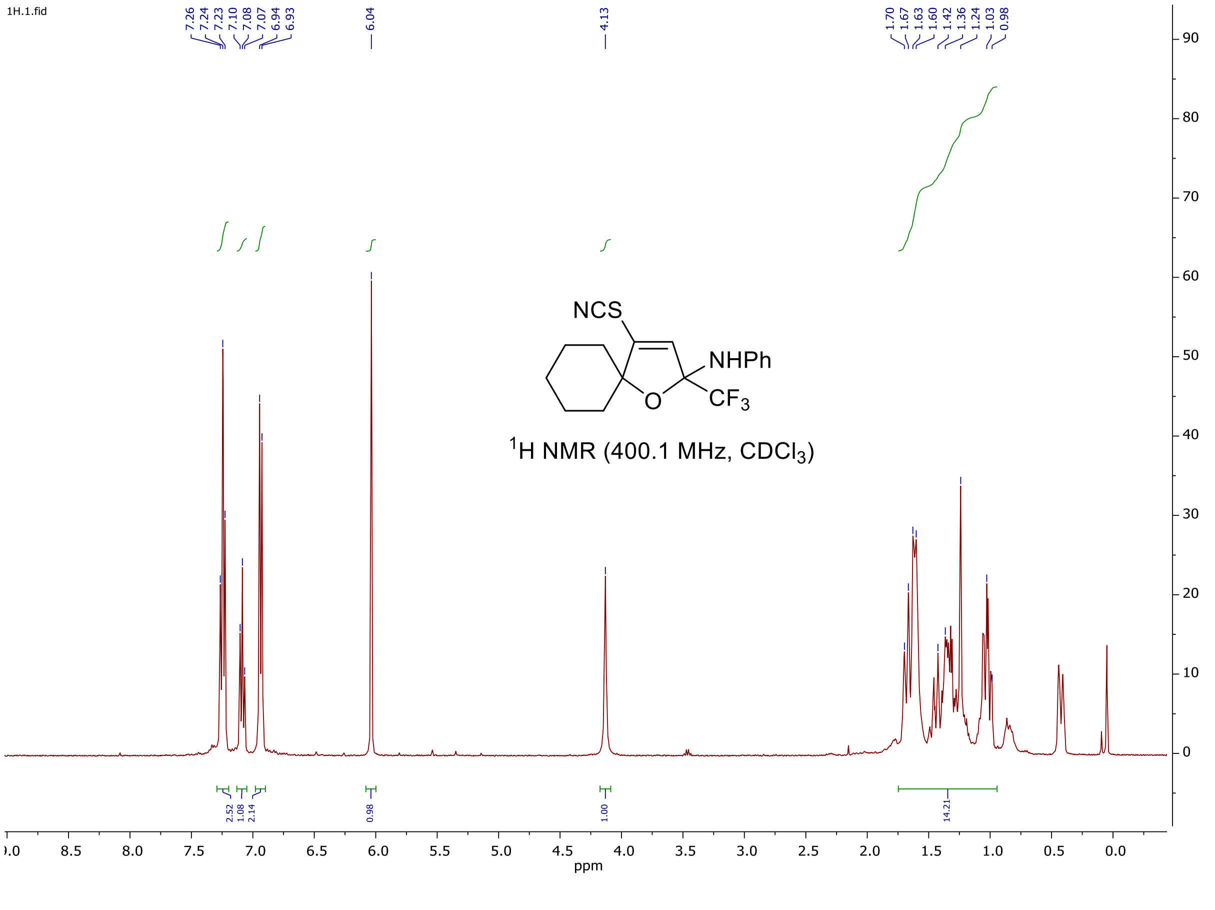

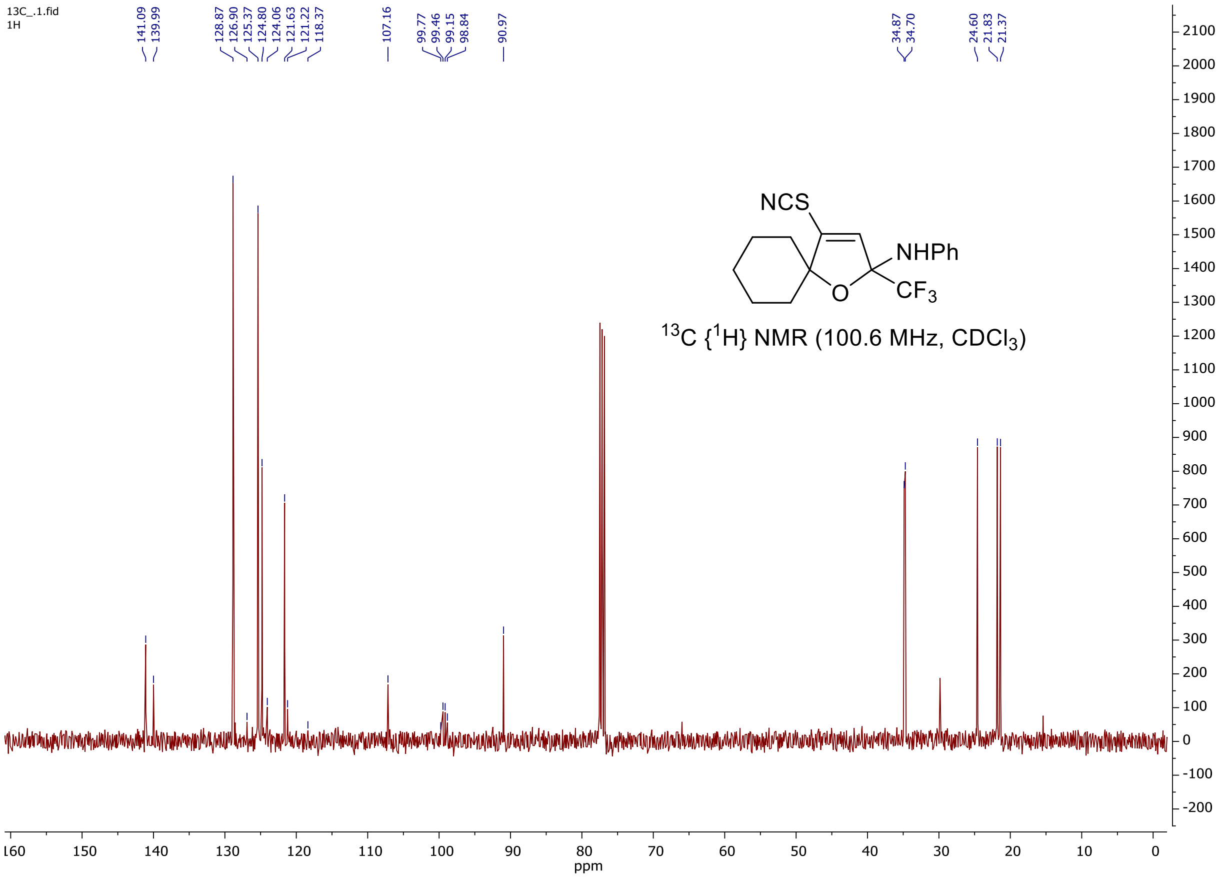
13C_.1.fid
1H
141.09
139.99
128.87
126.90
125.37
124.80
124.06
121.63
121.22
118.37
107.16
99.77
99.46
99.15
98.84
90.97
34.87
34.70
24.60
21.83
21.37
NCS
NHPh
O
CF3
13C {1H} NMR (100.6 MHz, CDCl3)
ppm

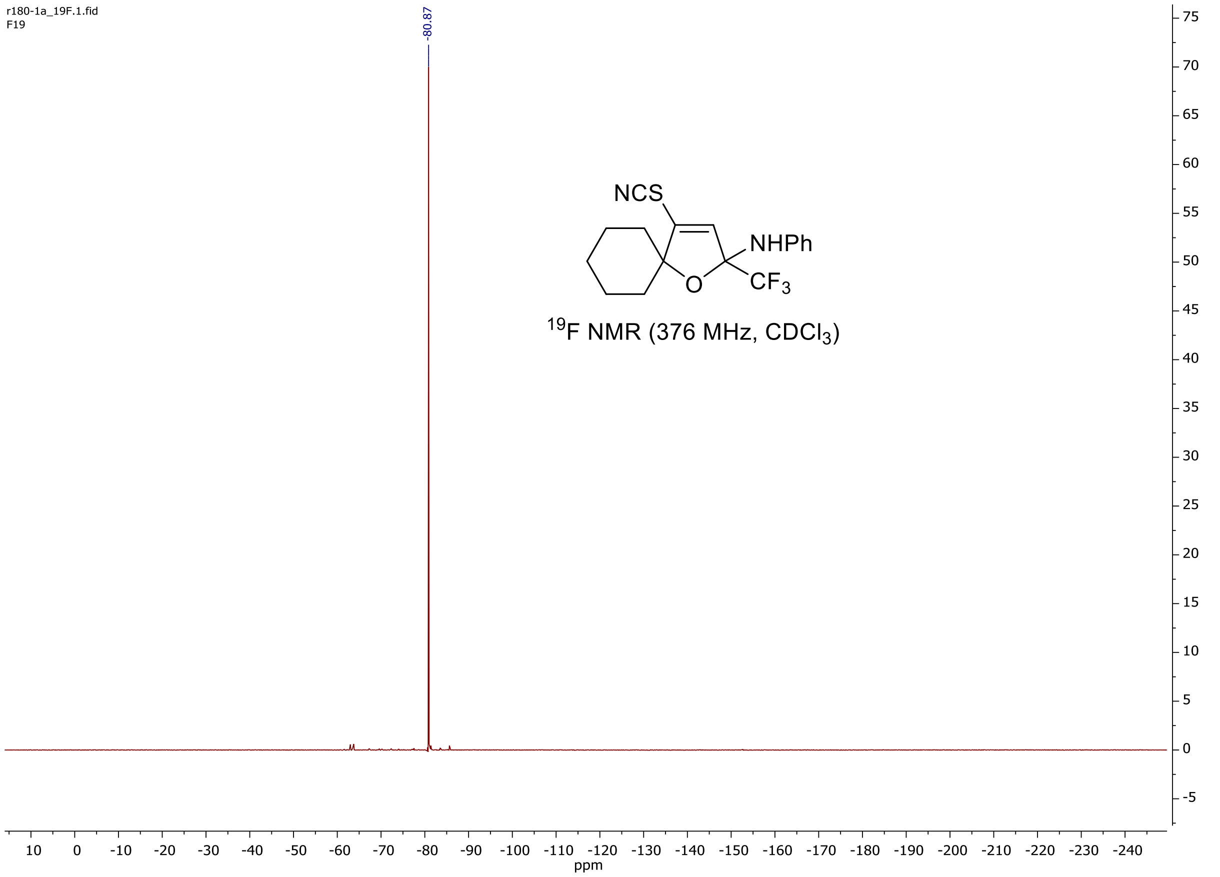
r180-1a_19F.1.fid
F19
-80.87
NCS
NHPh
O
CF3
19F NMR (376 MHz, CDCl3)
ppm

5-Methyl-*N*,5-diphenyl-4-thiocyanato-2-(trifluoromethyl)-2,5-dihydrofuran-2-amine (3d)

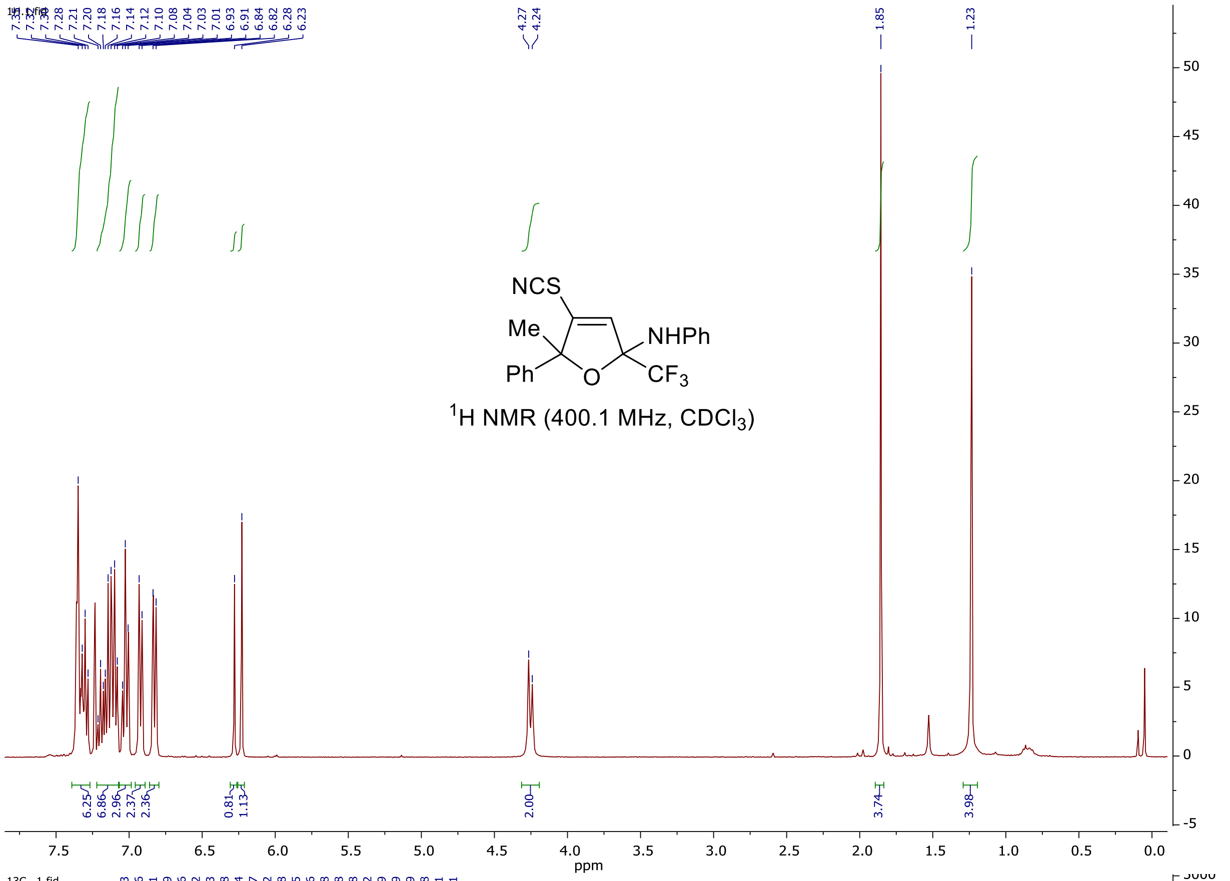

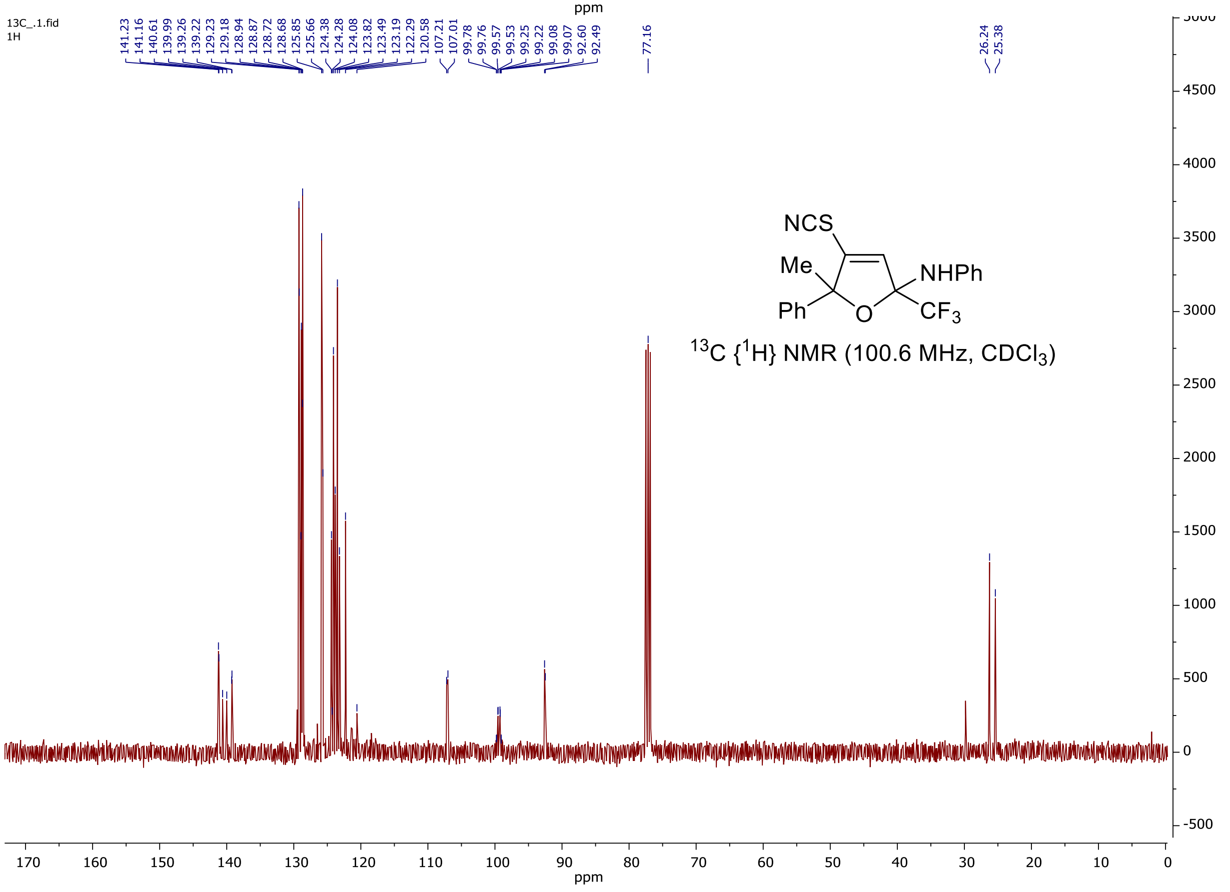

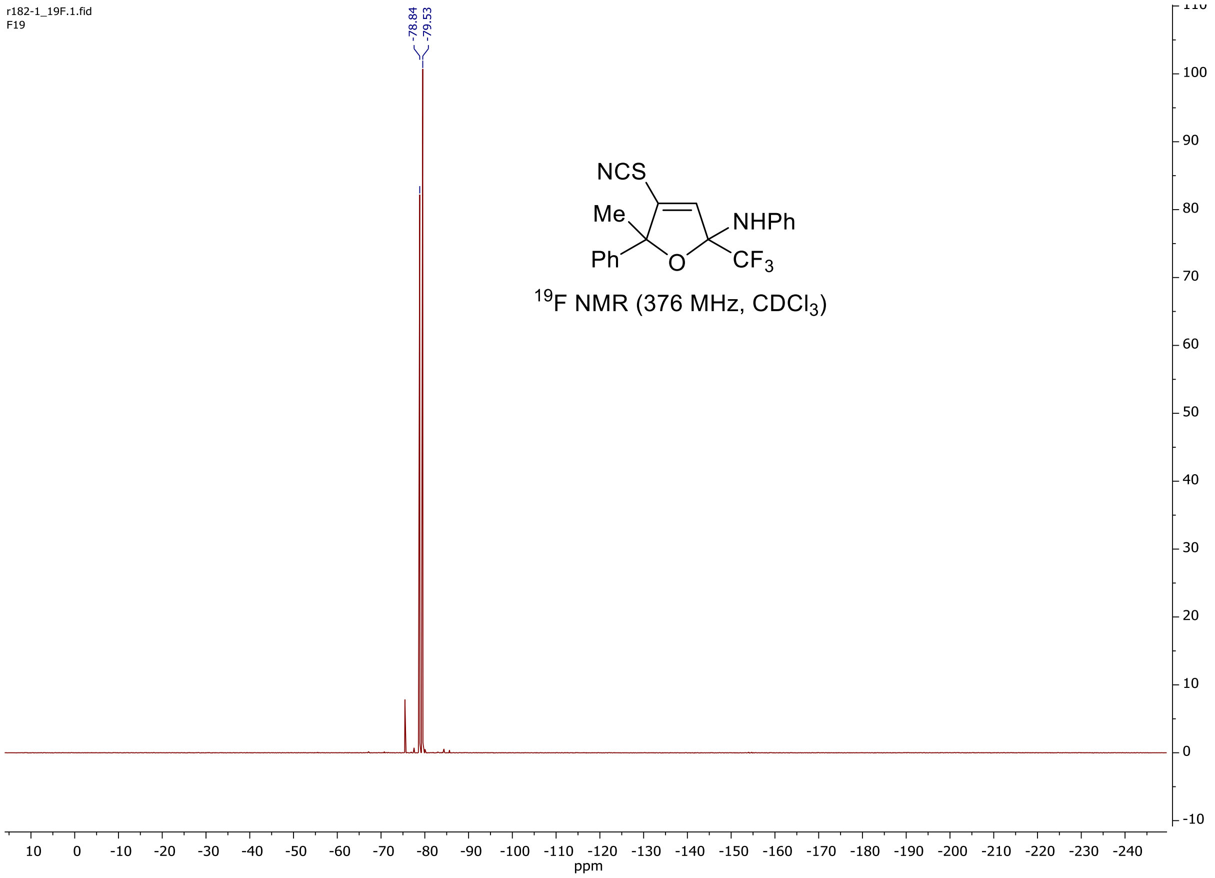

N,5,5-Triphenyl-4-thiocyanato-2-(trifluoromethyl)-2,5-dihydrofuran-2-amine (3e)

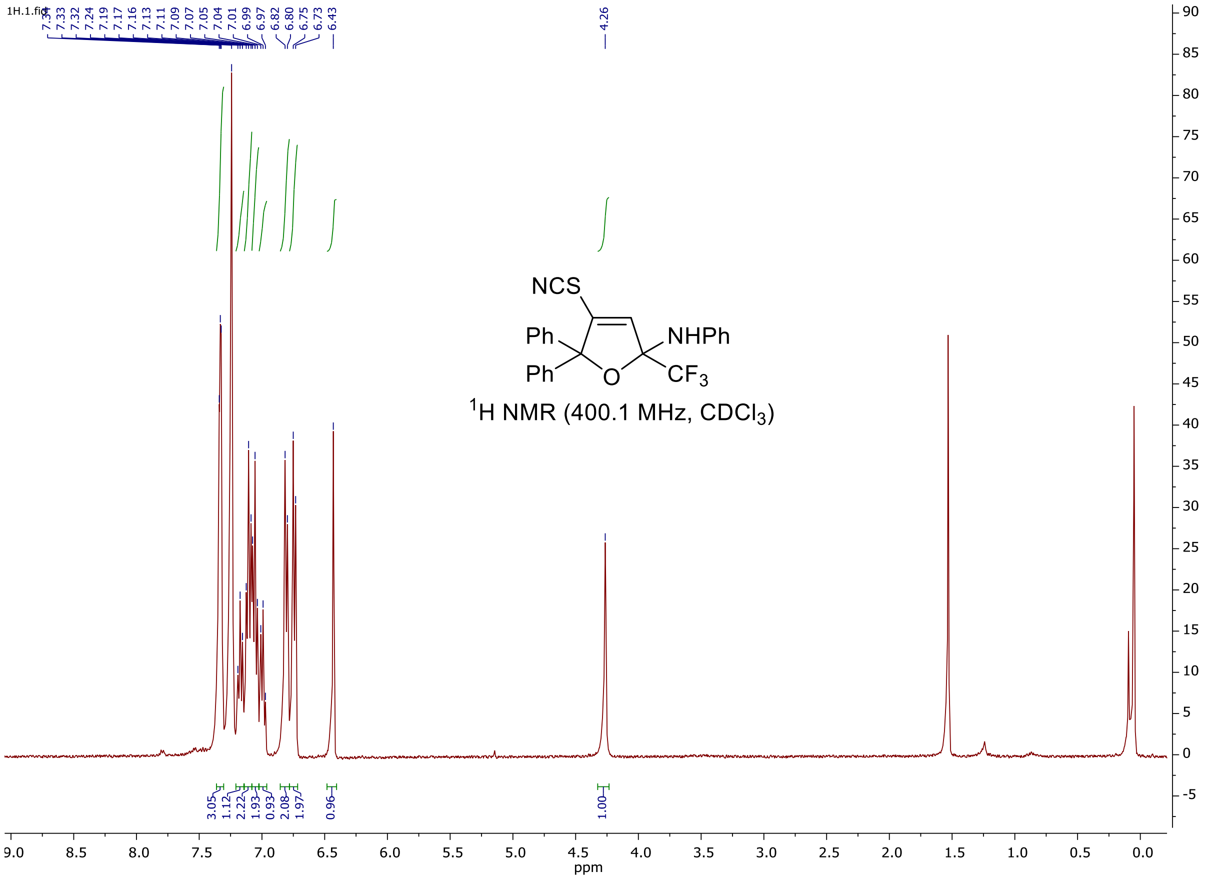

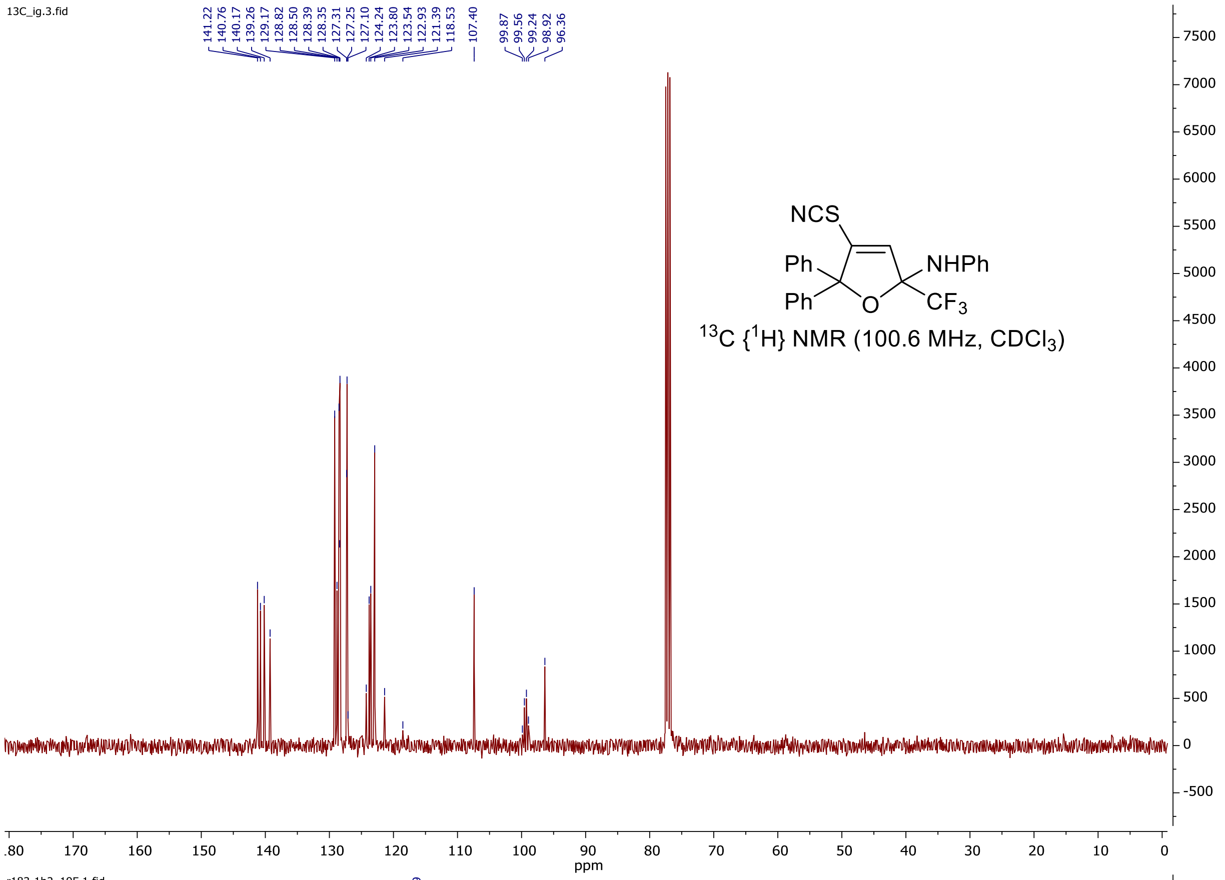
13C_ig.3.fid
141.22
140.76
140.17
139.26
129.17
128.82
128.50
128.39
128.35
127.31
127.25
127.10
124.24
123.80
123.54
122.93
121.39
118.53
107.40
99.87
99.56
99.24
98.92
96.36
NCS
Ph
NHPh
Ph
O
CF3
13C {1H} NMR (100.6 MHz, CDCl3)
ppm

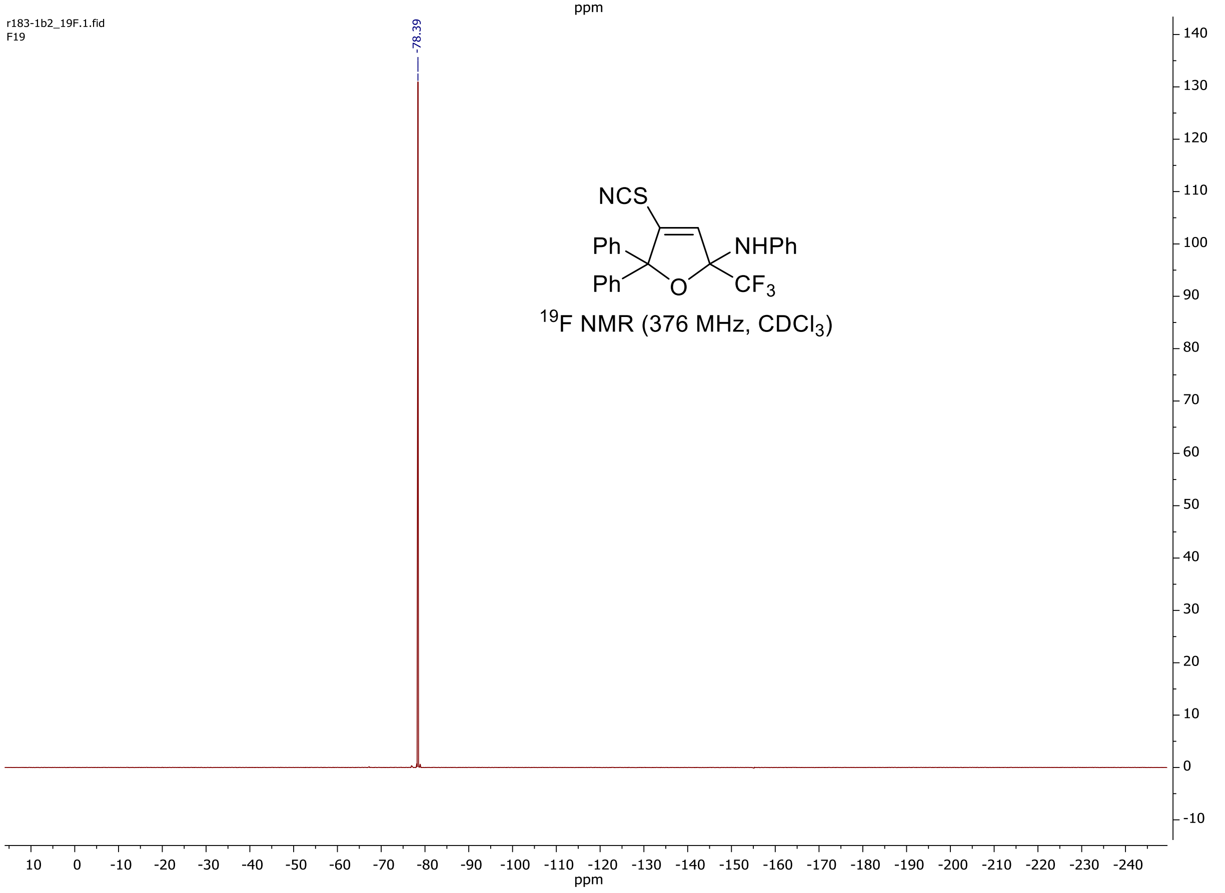
r183-1b2_19F.1.fid
F19
-78.39
NCS
Ph
NHPh
Ph
O
CF3
19F NMR (376 MHz, CDCl3)
ppm

5-Methyl-*N*-phenyl-4-thiocyanato-2-(trifluoromethyl)-2,5-dihydrofuran-2-amine (3f)

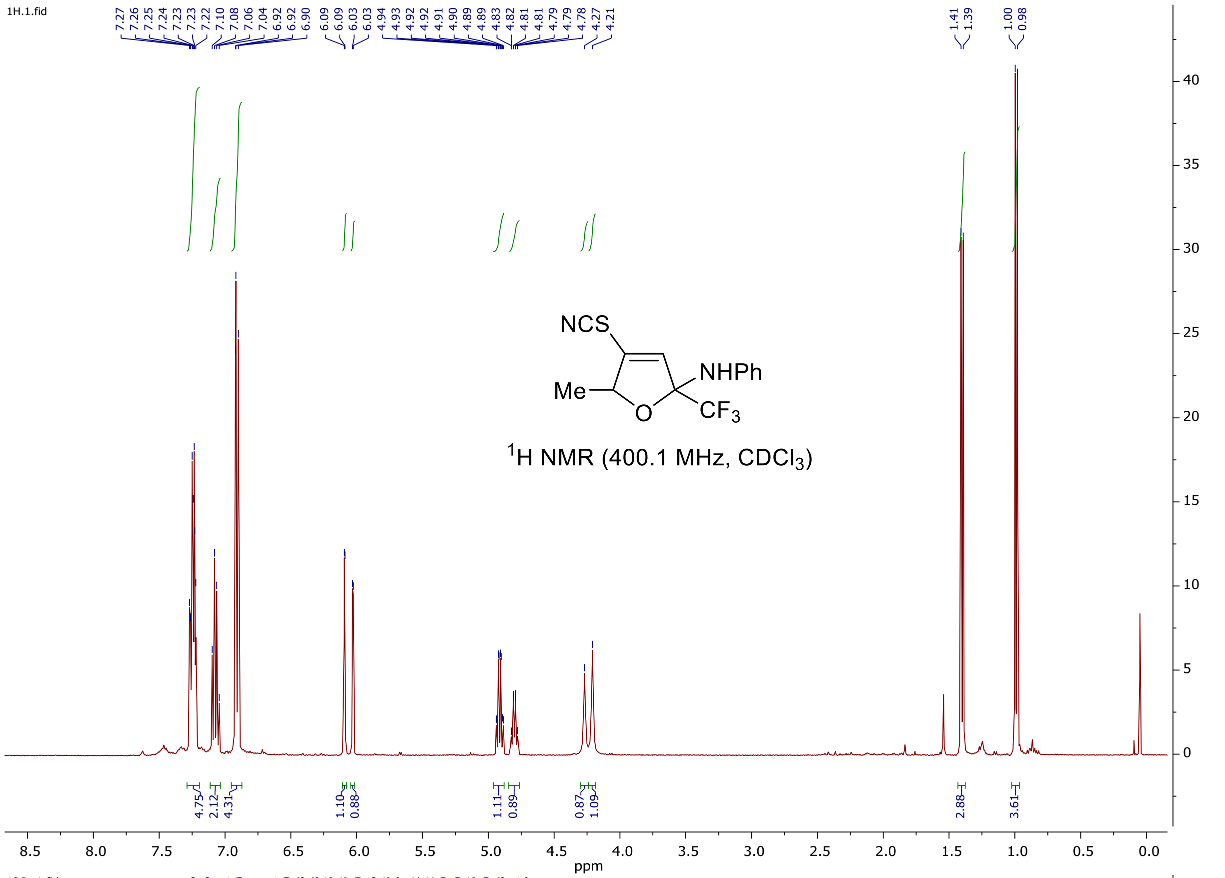

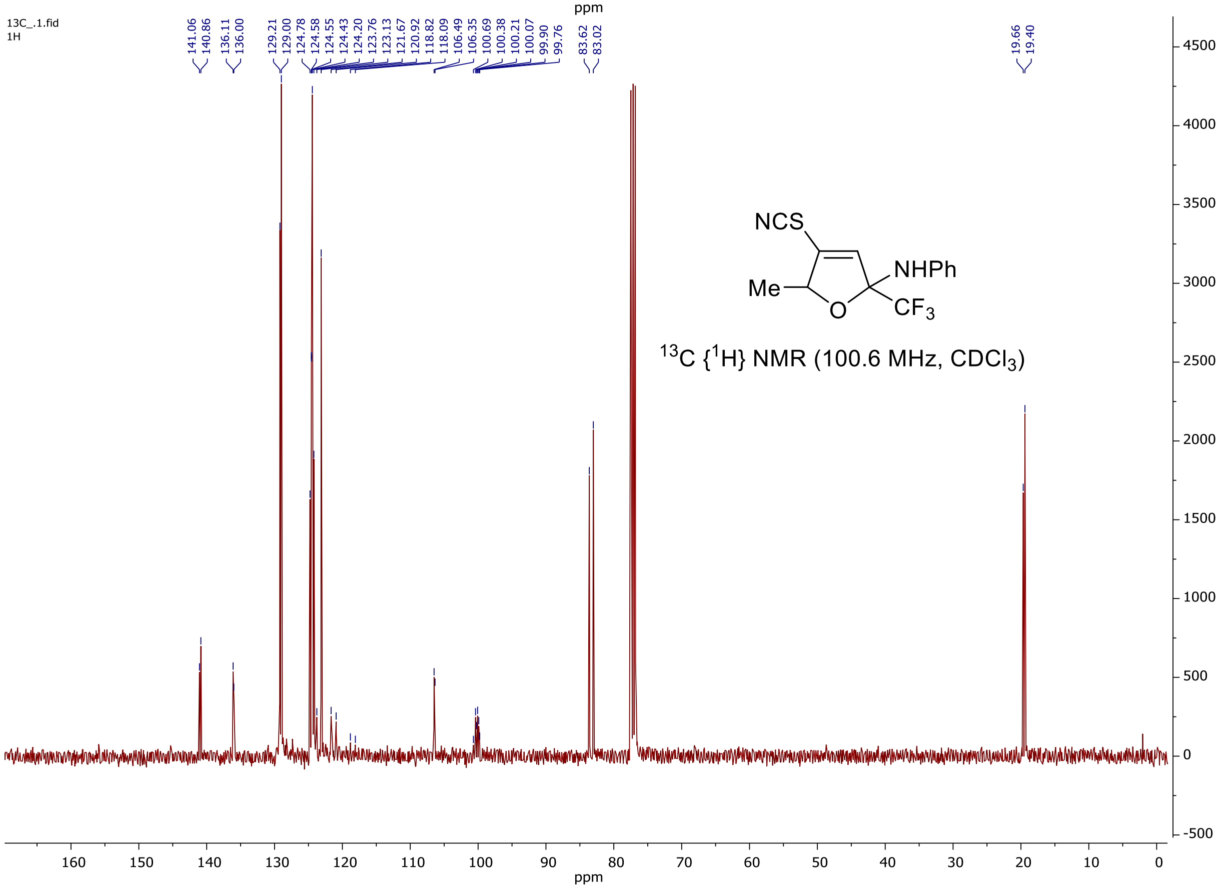

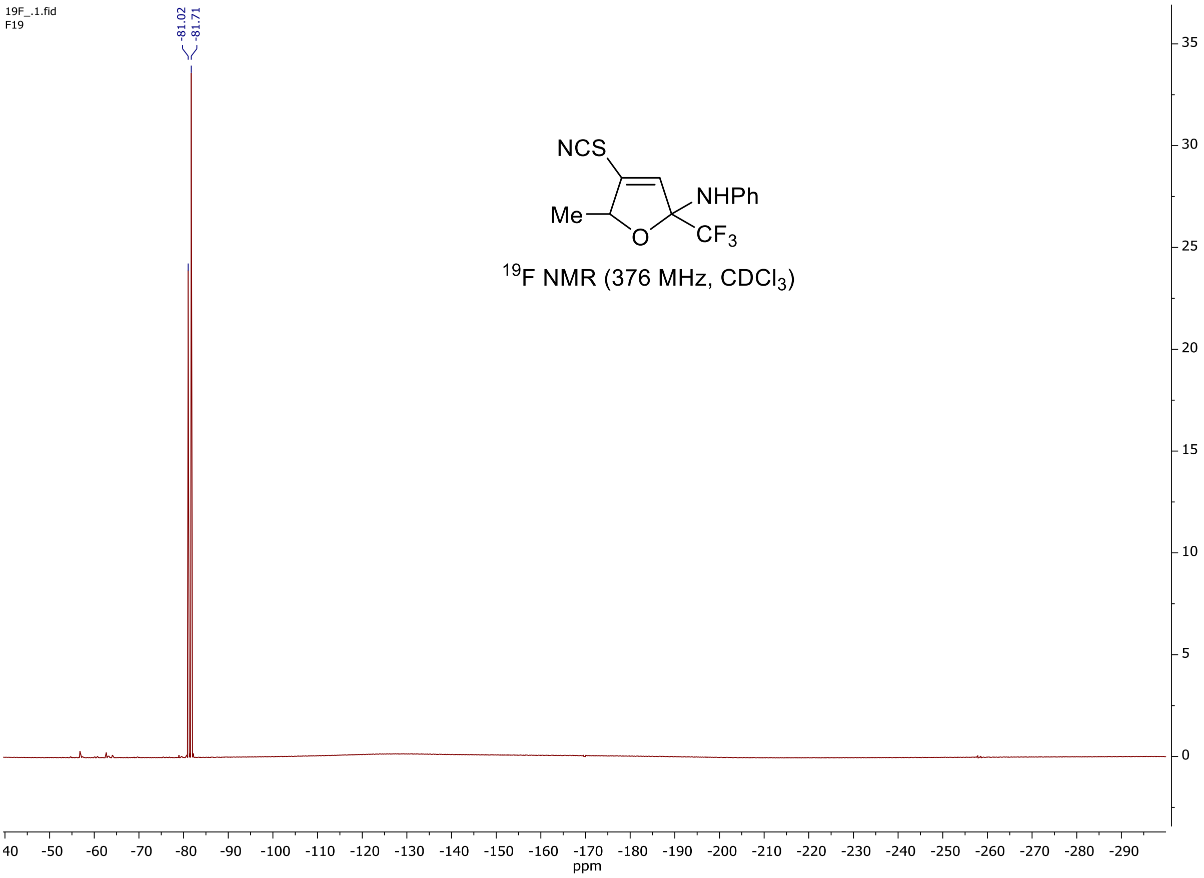

N-Phenyl-4-thiocyanato-2-(trifluoromethyl)-2,5-dihydrofuran-2-amine (3g)

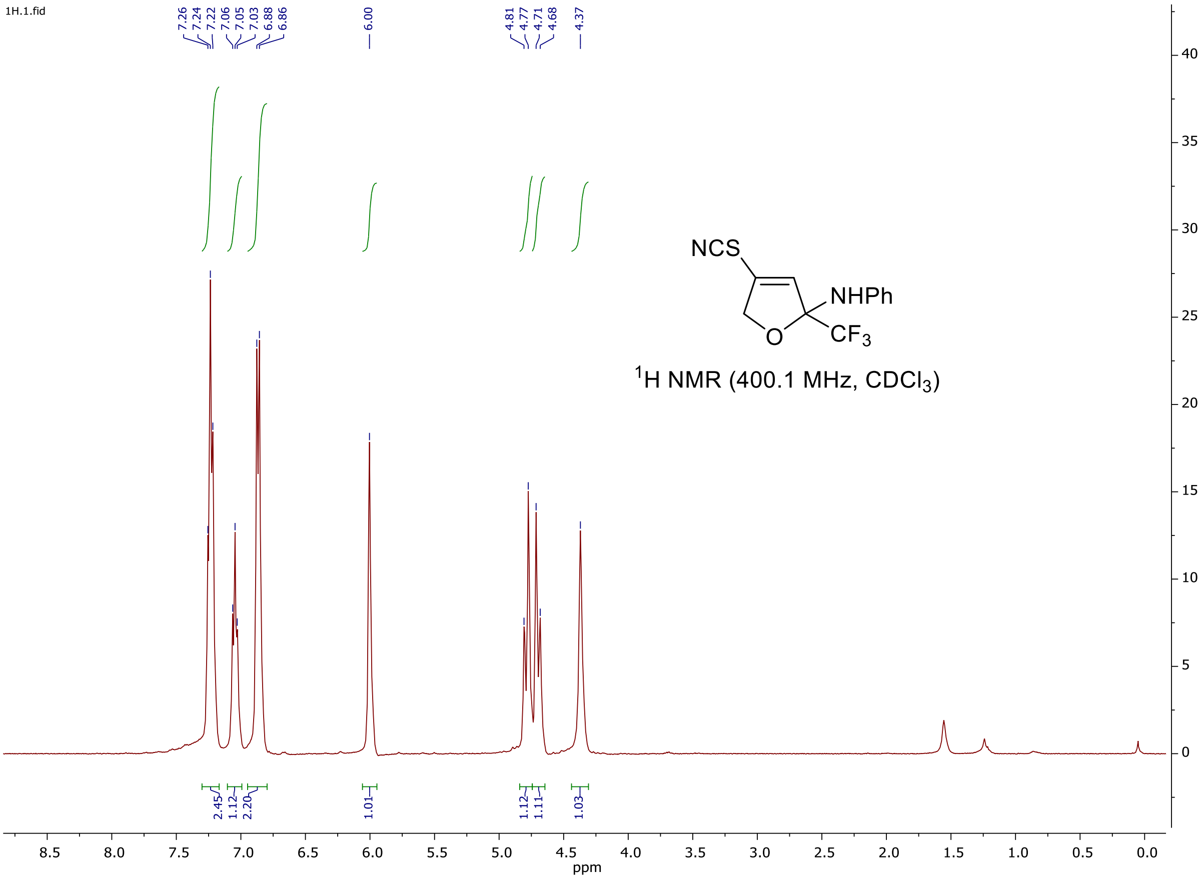

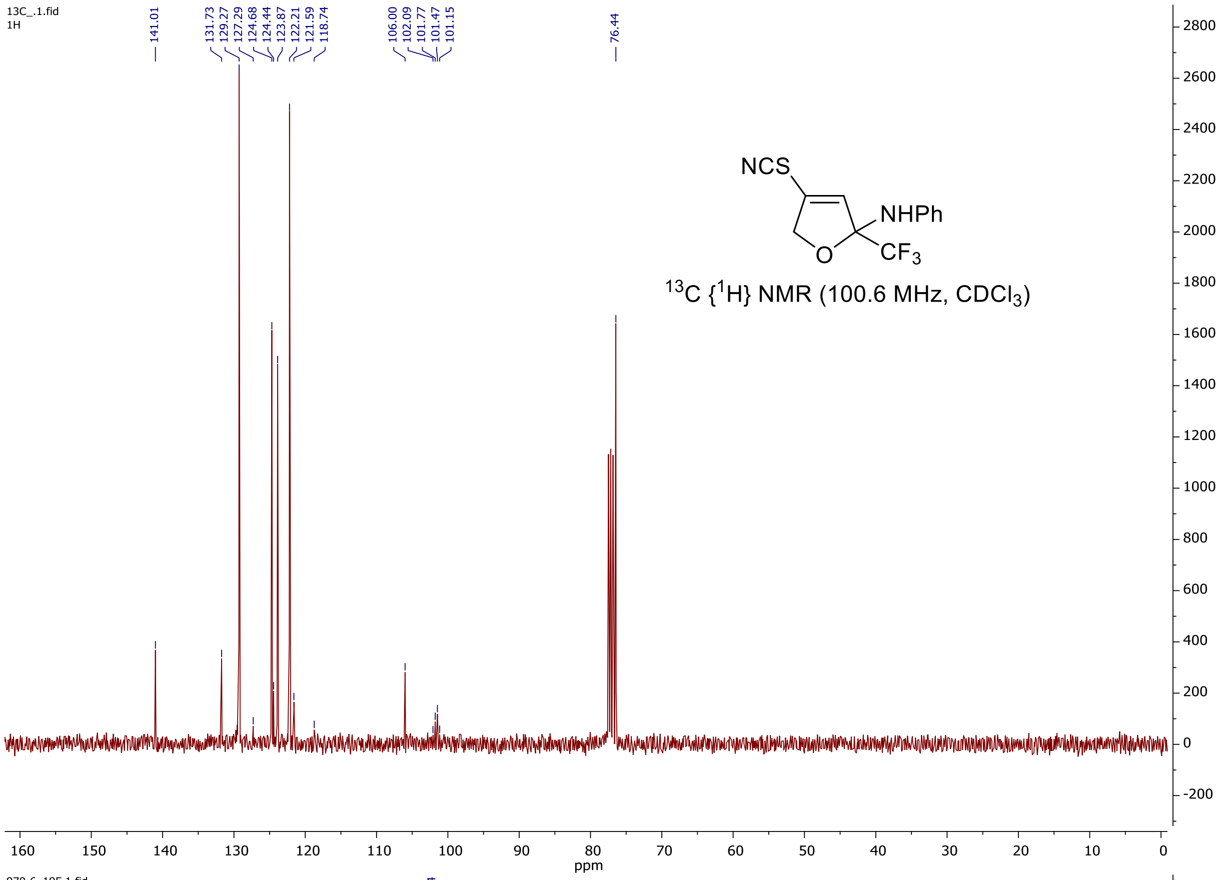
13C_.1.fid
1H
141.01
131.73
129.27
127.29
124.68
124.44
123.87
122.21
121.59
118.74
106.00
102.09
101.77
101.47
101.15
76.44
NCS
NHPh
O
CF3
13C {1H} NMR (100.6 MHz, CDCl3)
ppm

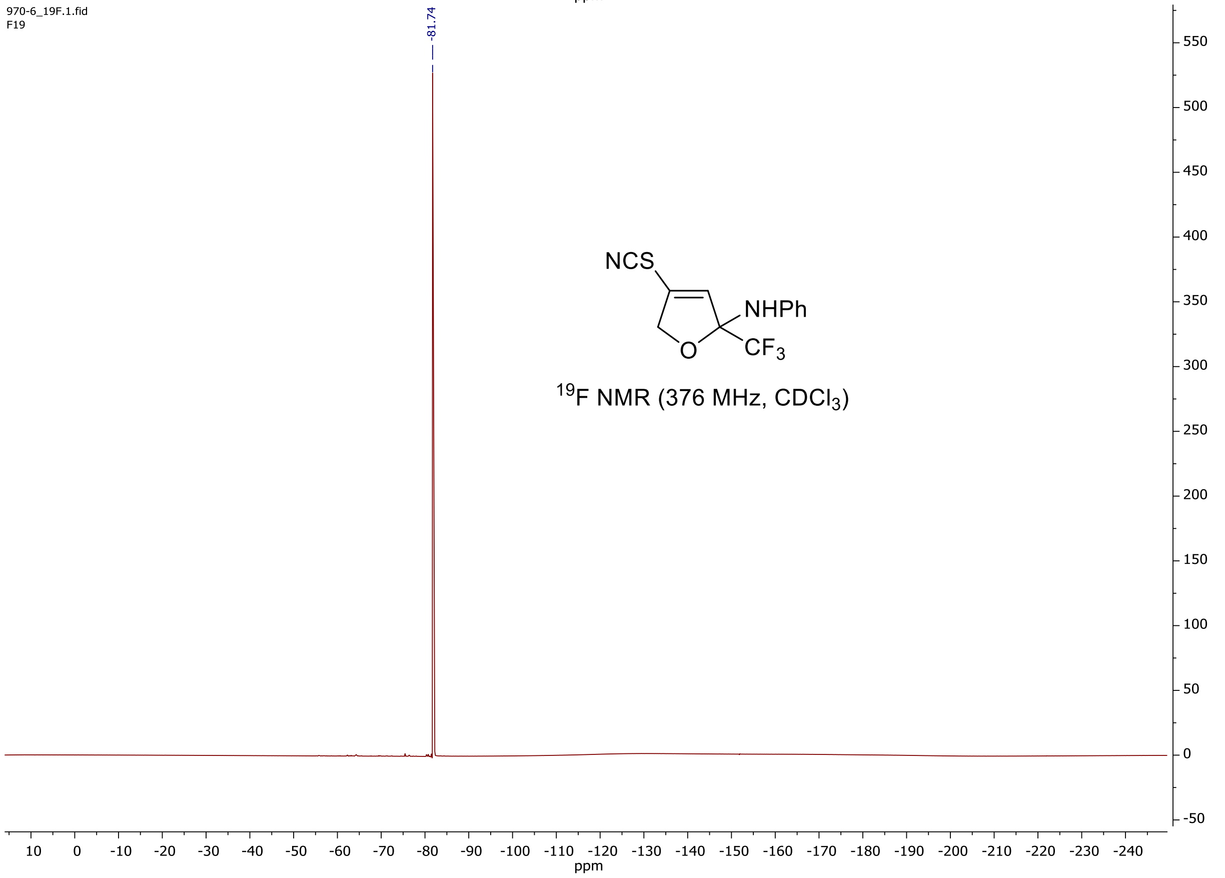
970-6_19F.1.fid
F19
-81.74
NCS
NHPh
O
CF3
19F NMR (376 MHz, CDCl3)
ppm

N-Butyl-5,5-dimethyl-4-thiocyanato-2-(trifluoromethyl)-2,5-dihydrofuran-2-amine (3h)

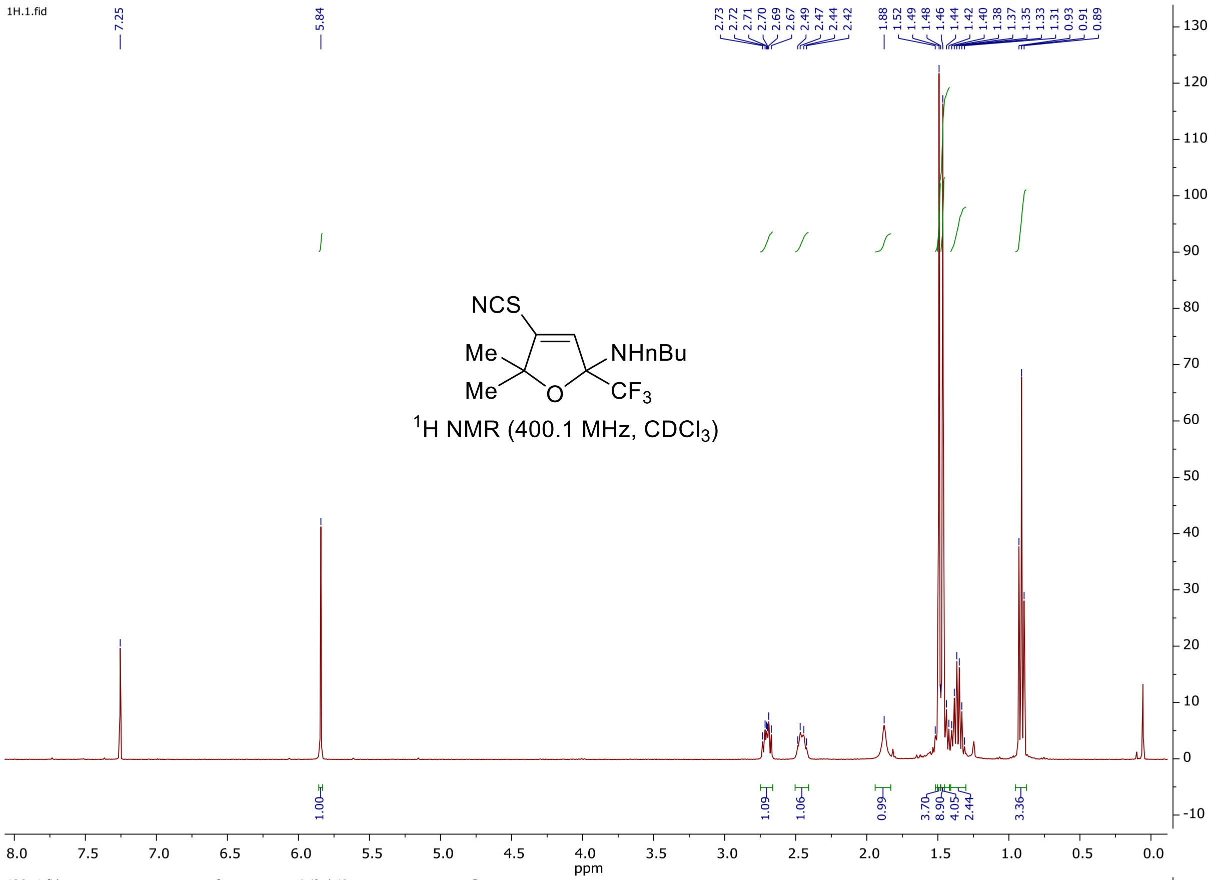

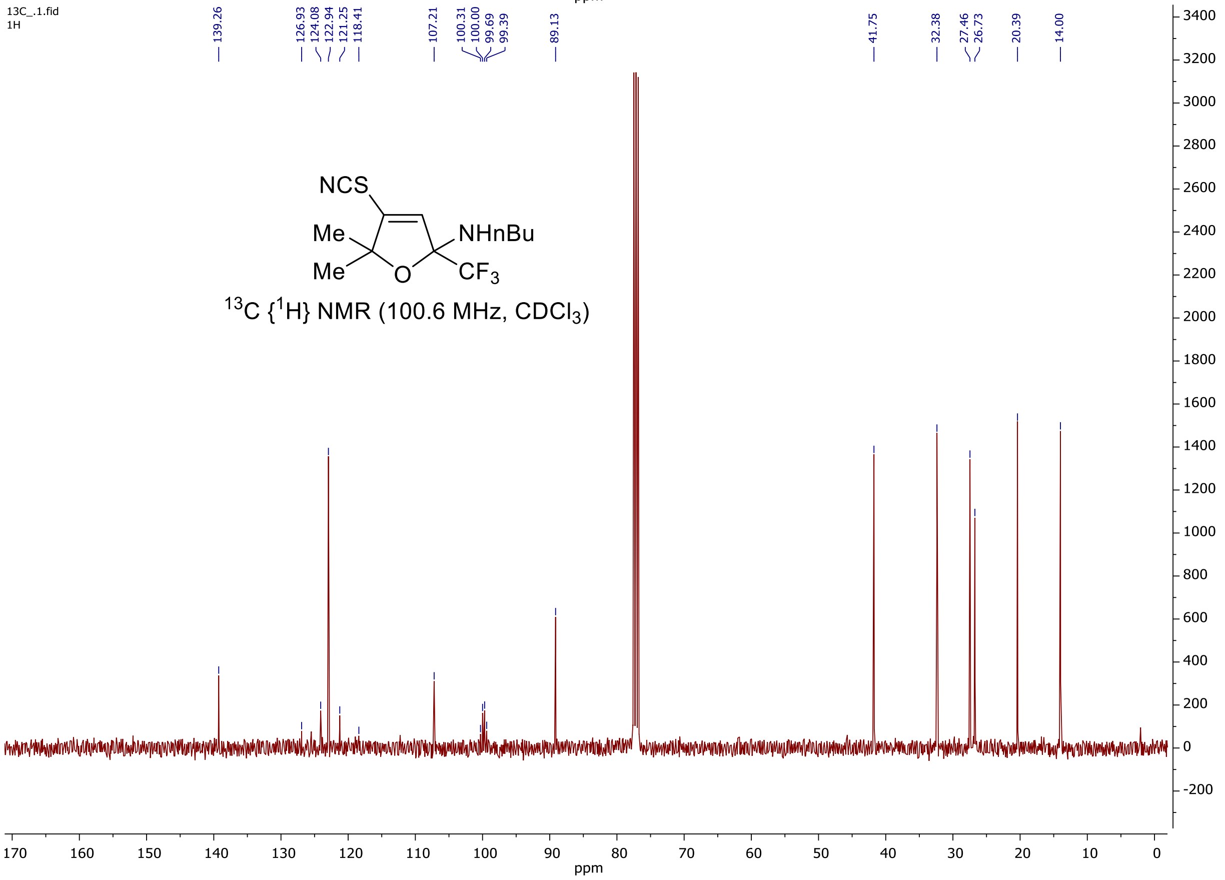

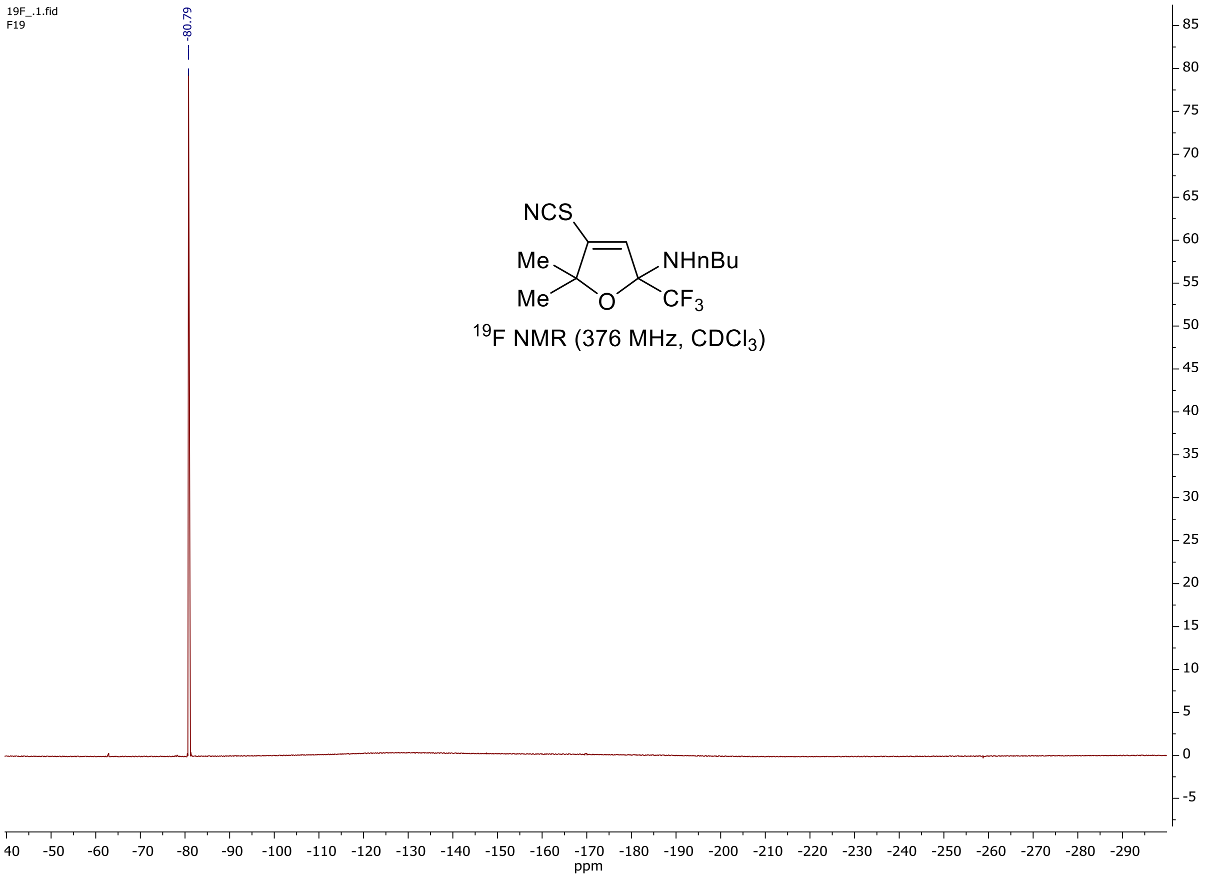

5,5-Dimethyl-2-(perfluoropropyl)-*N*-phenyl-4-thiocyanato-2,5-dihydrofuran-2-amine (3i)

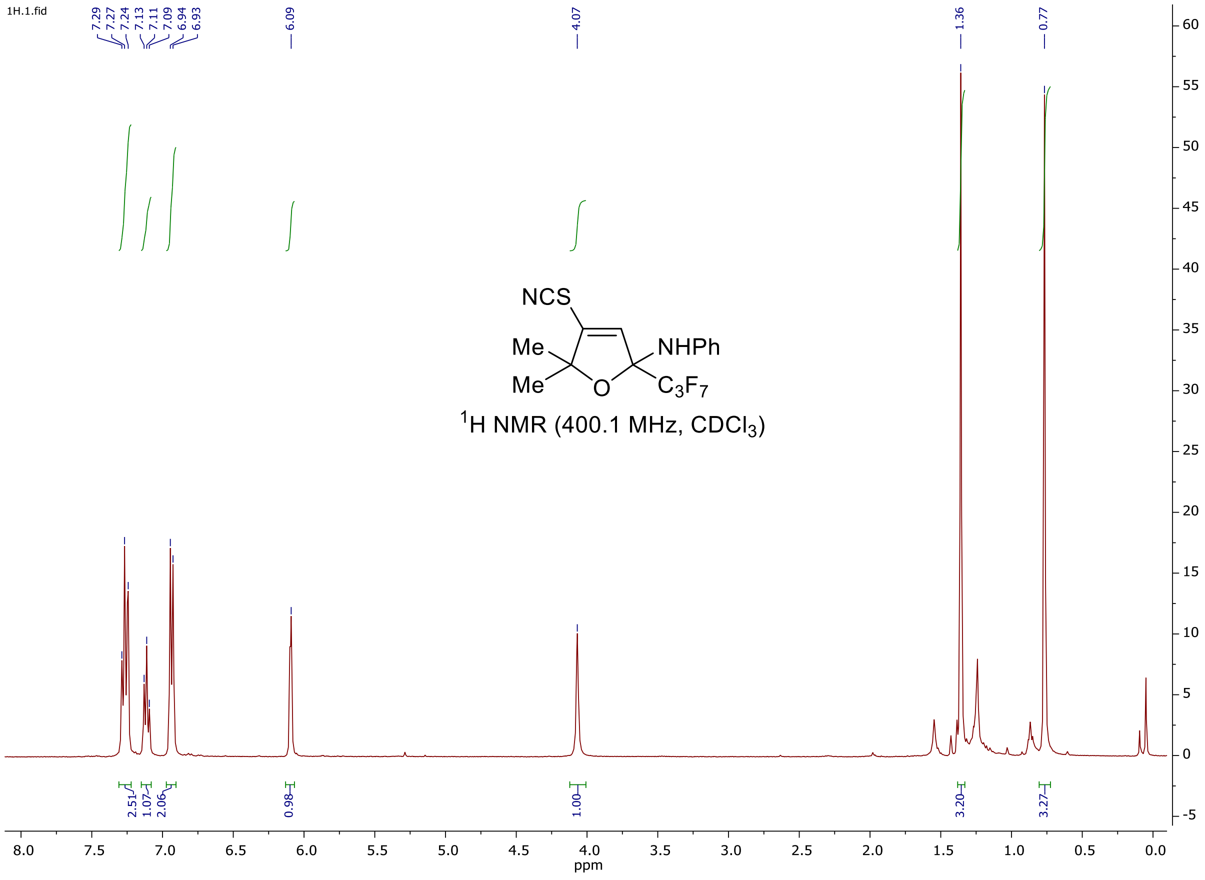

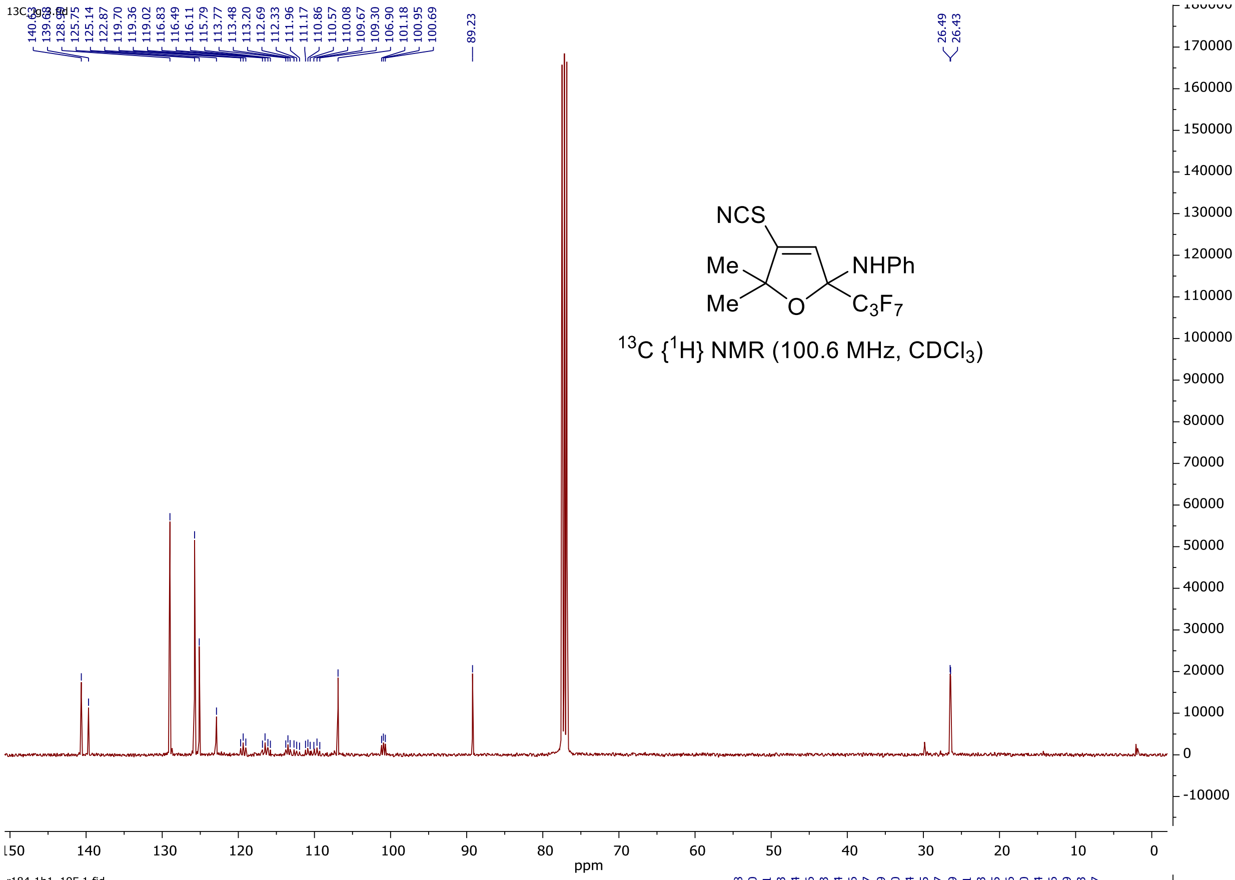
13C{1H} NMR (100.6 MHz, CDCl3)
NCS
Me
Me
O
NHPh
C3F7
ppm

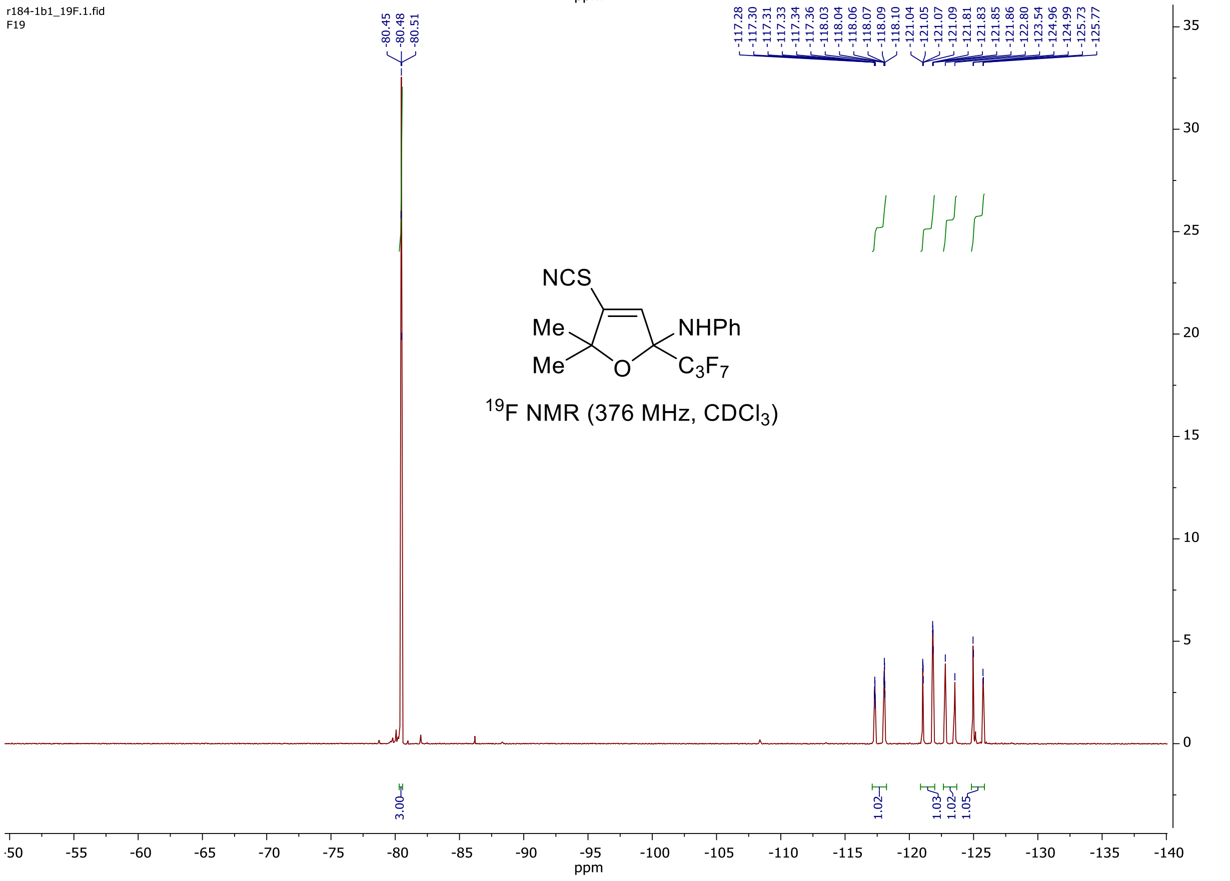
r184-1b1_19F.1.fid
F19
19F NMR (376 MHz, CDCl3)
NCS
Me
Me
O
NHPh
C3F7
ppm

10. NMR (^{1}H, ^{13}C) spectra of the 3-hydroxy-5-(2-hydroxypropan-2-yl)-2-phenyl-3-(trifluoromethyl)-2,3-dihydroisothiazole 1,1-dioxide (4)

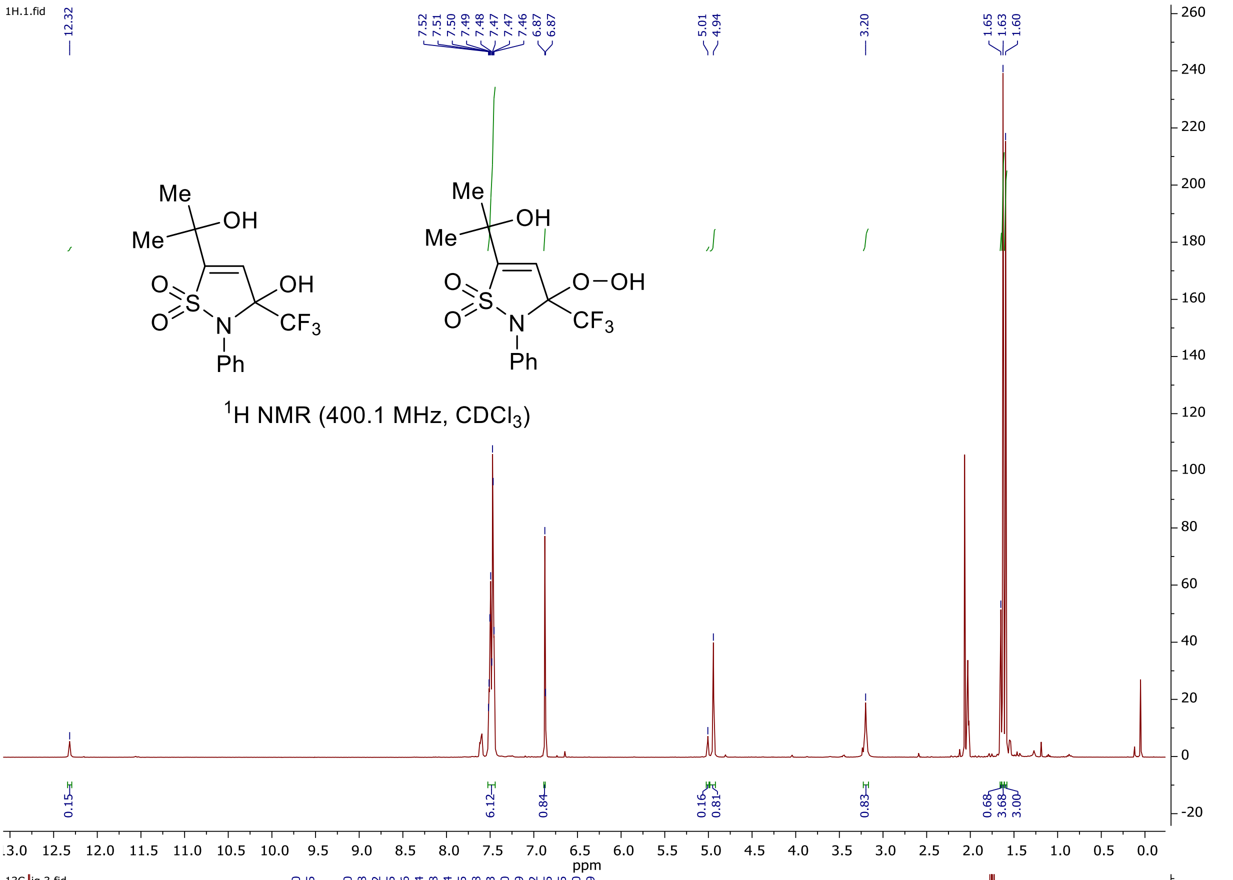

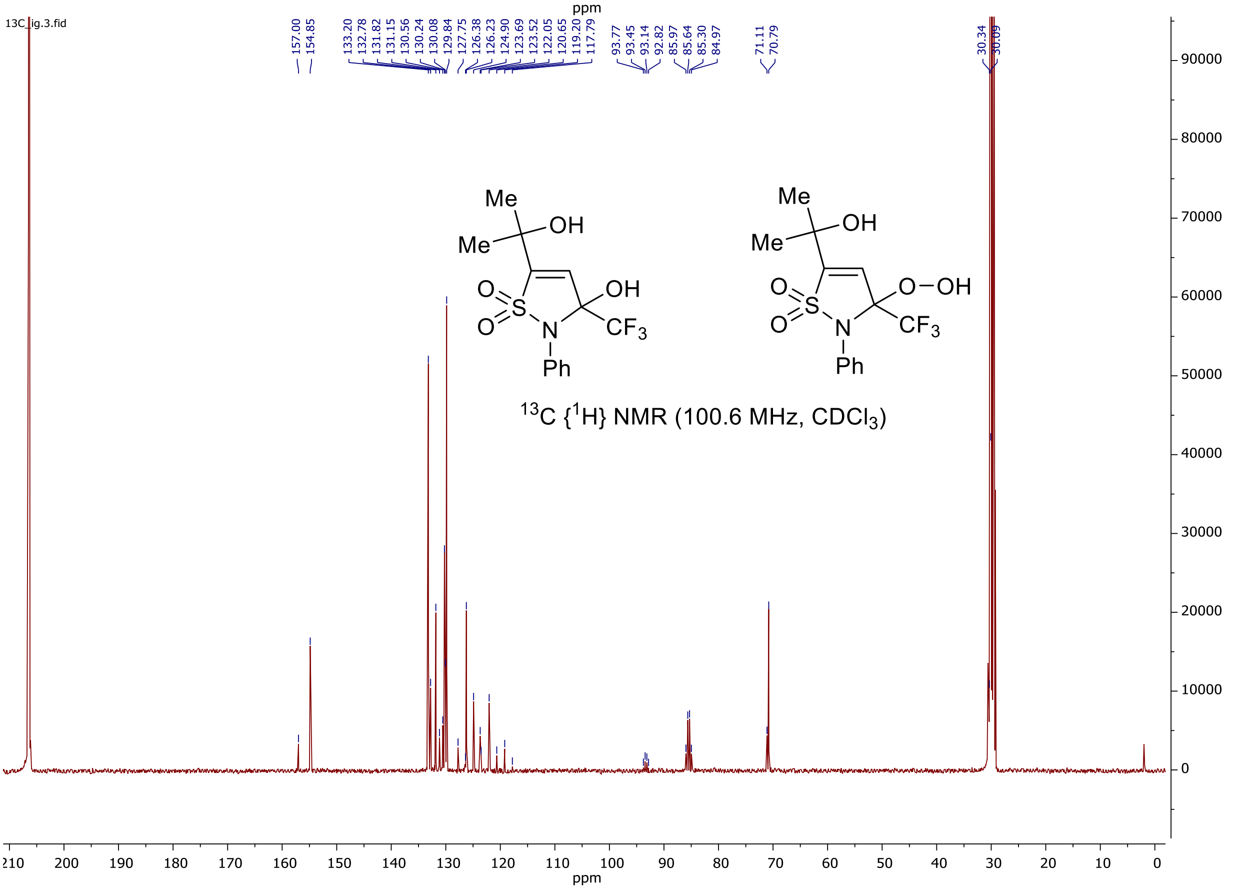

Supplement: File 1 — Full experimental details, characterization data and copies of NMR spectra for all new compounds. [file Beilstein_J_Org_Chem-21-2694-s001.pdf]
